# *Nepenthes* × *ventrata* Transcriptome Profiling Reveals a Similarity Between the Evolutionary Origins of Carnivorous Traps and Floral Organs

**DOI:** 10.3389/fpls.2021.643137

**Published:** 2021-05-28

**Authors:** Anna V. Shchennikova, Alexey V. Beletsky, Mikhail A. Filyushin, Maria A. Slugina, Eugeny V. Gruzdev, Andrey V. Mardanov, Elena Z. Kochieva, Nikolay V. Ravin

**Affiliations:** Institute of Bioengineering, Research Center of Biotechnology, The Russian Academy of Sciences, Moscow, Russia

**Keywords:** plant carnivory, *Nepenthes*, pitcher, transcriptome analysis, MADS-box family transcription factors

## Abstract

The emergence of the carnivory syndrome and traps in plants is one of the most intriguing questions in evolutionary biology. In the present study, we addressed it by comparative transcriptomics analysis of leaves and leaf-derived pitcher traps from a predatory plant *Nepenthes ventricosa* × *Nepenthes alata*. Pitchers were collected at three stages of development and a total of 12 transcriptomes were sequenced and assembled *de novo*. In comparison with leaves, pitchers at all developmental stages were found to be highly enriched with upregulated genes involved in stress response, specification of shoot apical meristem, biosynthesis of sucrose, wax/cutin, anthocyanins, and alkaloids, genes encoding digestive enzymes (proteases and oligosaccharide hydrolases), and flowering-related MADS-box genes. At the same time, photosynthesis-related genes in pitchers were transcriptionally downregulated. As the MADS-box genes are thought to be associated with the origin of flower organs from leaves, we suggest that *Nepenthes* species could have employed a similar pathway involving highly conserved MADS-domain transcription factors to develop a novel structure, pitcher-like trap, for capture and digestion of animal prey during the evolutionary transition to carnivory. The data obtained should clarify the molecular mechanisms of trap initiation and development and may contribute to solving the problem of its emergence in plants.

## Introduction

Predator plants attract arthropods that serve both as pollinators for sexual reproduction and as prey captured and digested in traps (active or passive); traps represent modified leaves developed to support plant survival in the nutrient-deficient environment (Jürgens et al., 2012; Pavlovič and Saganová, 2015; Bartlett, 2017). It is considered that carnivory evolved independently nine times; in particular, passive pitfall traps have at least six autonomous origins in different orders of flowering plants (Givnish, 2015). To date, the list of green predators contains more than 650 species representing 19 genera, 12 families, and five orders of the flowering plants, both monocots and eudicots (Pavlovič and Saganová, 2015). The most diverse lineage of carnivorous plants, Caryophyllales, includes one of the most famous genera of pitcher plants, *Nepenthes*, which comprises 160–180 species as well as numerous natural and cultivated hybrids, inhabiting humid and sunny lowlands or tropical mountains deficient in nitrogenous nutrients soils (Murphy et al., 2020). Because of the large number of *Nepenthes* species residing in diverse habitats, the genus has become a model for studying plant adaptive radiation and speciation (Thorogood et al., 2018).

*Nepenthes* species develop typical photosynthetic leaves whose tips form a tendril as an extension of the midrib, to which a pitcher-like pitfall trap is attached (Owen and Lennon, 1999). The trap has a leaf-like lid (operculum), which initially seals the growing pitcher until it is ripe and ready to capture the prey; it also prevents fluid dilution during rain in mature traps (Bauer et al., 2008). Once open, the pitcher attracts the prey with rim (peristome)-located nectaries forming a slippery zone covered with a thick wax layer to catch and prevent the escape of the prey which then falls into a lower digestive zone containing multicellular glands that secrete an acidic fluid saturated with hydrolytic enzymes (chitinases and proteases) and antifungal factors (Gaume and Forterre, 2007; Scholz et al., 2010; Pavlovic̀ and Mithöfer, 2019). The main pitcher characteristics attracting the prey are color patterns (UV fluorescence/visible wavebands), sweet fragrance of the secreted extrafloral nectar, and high CO_2_ levels (Kurup et al., 2013; Baby et al., 2017).

Most studies on *Nepenthes* focus on the morphophysiological and functional characteristics of the pitcher, including biochemical composition of the secreted fluid in the digestive zone and transcriptomic and proteomic changes in response to prey and during early pitcher opening (Gorb et al., 2004; Hatano and Hamada, 2008, 2012; Rottloff et al., 2016; Wan Zakaria et al., 2016, 2019; Baby et al., 2017; Zulkapli et al., 2017; Goh et al., 2020); however, the data regarding the evolutionary transition from the non-carnivorous to carnivorous status in Nepenthes species are scarce. Comparative transcriptomics of carnivorous and non-carnivorous leaves in *Cephalotus follicularis* identified genetic signatures associated with prey attraction, capture, digestion, and nutrient absorption underlying the developmental switch between carnivorous and non-carnivorous leaf state (Fukushima et al., 2017). Recent transcriptomic analysis of genes controlling pitcher development in *Nepenthes khasiana* has revealed that ASYMMETRIC LEAVES 1 (AS1) and ERECTA (ER) contribute to the formation of the tendril from the midrib, whereas REVOLUTA (REV) may be positively associated with the initiation and maturation of the pitcher (Dkhar and Pareek, 2019). The most intriguing question is identification of molecular pathways that directed the emergence of the carnivory syndrome, as there are examples of both convergent (across unrelated lineages) and divergent (within genera) evolution of the trap (Thorogood et al., 2018). The pitcher-like trap has diverse morphology adapted to trapping and digestion of various preys. This is especially characteristic for the species of the *Nepenthes* genus, which is suggested to have originated from a *Drosera*-like predecessor (Givnish, 2015; Murphy et al., 2020). A comparative analysis of the genome of three carnivorous Droseraceae species showed that the evolution of predation in this family could be facilitated by a whole-genome duplication (WGD) in their last common ancestor, followed by diversification of the multiplied genes, including recruitment of root-specific genes to developing traps, massive loss of genes involved in non-carnivorous nutrition, and expansion of gene families associated with the attracting, catching, digesting, and utilizing prey (Palfalvi et al., 2020). However, the molecular mechanism employed by carnivorous plants to develop such a novel structure is still unclear.

In this study, we aimed to identify transcriptional signatures of the transition from the leaf to the mature pitfall trap by performing comparative transcriptomics of *Nepenthes* × *ventrata* leaves and pitchers at three developmental stages. The results revealed activation of genes associated with defense response, shoot apical meristem (SAM) organization, anthocyanin biosynthesis, and flowering, including MADS-domain transcription factors (TFs), in pitchers. Considering that MADS-box genes are thought to have been involved in the evolution of plant flower organs, we suggest that the emergence of the pitcher structure was also due to highly conserved MADS-domain TFs, which usually determine the identity of floral meristems and organs in extant plants.

## Materials and Methods

### Plant Materials and Growth Conditions

*Nepenthes* × *ventrata* Hort. ex Fleming (Nepenthaceae Dumort.) is a natural hybrid between two *Nepenthes* clade 2 species: *N. ventricosa* Blanco (Insignes clade) and *N. alata* Blanco (Graciiflora clade) endemic to the northern forests of the Philippines (Murphy et al., 2020). Plants were obtained from a nursery garden and grown in a greenhouse under controlled conditions (16/8-h light-dark cycle with light intensity of 150–200 μmol m^–2^ s^–1^, 20/24°C night/day temperature cycle, and 75–90% humidity). Samples of mature leaves at the middle section and pitchers at three developmental stages: primordial pitcher (or early pitcher) (∼1 cm), unopened young pitcher (3–4 cm), and open mature pitcher (or late pitcher) (7–11 cm; the first day of opening; there are no insects inside) were collected from the upper part of plant; three biological replicates were analyzed.

### RNA Isolation, Library Preparation, and Transcriptome Sequencing

Total RNA was extracted from 300 mg of each of the 12 tissue samples using a modified CTAB-based technique (Filyushin et al., 2019) to facilitate extraction from wax-coated *Nepenthes* leaves and pitchers and treated with the RNase-free DNase Set (Qiagen, United States). RNA was assessed for quality using BioSpectrometer (Eppendorf, United States) and quantity using the Qubit^®^ RNA Assay Kit and Qubit^®^ 2.0 Fluorometer (Life Technologies, United States). According to the RNA integrity number (RIN) measured using Agilent 2100 Bioanalyzer (Agilent Technologies Inc., United States), all 12 RNA samples were of good quality for gene expression analysis by RNA-seq (RIN ∼8.4–9.7).

mRNA libraries were constructed using a NEBNext^®^ mRNA Library Prep Reagent Set for Illumina^®^ according to the manufacturer’s instructions (New England BioLabs, United States). Twelve barcoded libraries were sequenced by MyGene Co. (Moscow, Russia) on Illumina HiSeq2500 (Illumina, United States), generating a total of 387,930,413 single-end reads (200 bp) and the data were verified by additional in-house sequencing on Illumina MiSeq according to the manufacturer’s instructions (Illumina). In total, 23,277,185 paired-end reads (2 × 250 bp) were generated.

### *De novo* Transcriptome Assembly, Identification of Protein Coding Regions, and Annotation

Before assembly, adapter sequences were removed from the reads using cutadapt v1.17 (Martin, 2011), low quality reads were trimmed using sickle v1.33^1^, and paired overlapping reads were merged using FLASH v1.2.11. *De novo* transcriptome assembly was carried out using Trinity v2.6.5 (Haas et al., 2013) with default parameters, using all reads combined together.

Coding regions in the assembled transcripts were predicted by TransDecoder v 5.1.0^2^ within the Trinity software and the putative transcripts were screened for homology to known protein-coding plant genes using Diamond v.0.9 homology search in NCBI-NR database, and the completeness of the resulting assembly was assessed using BUSCO v.5.0.0 (Seppey et al., 2019). Potential contamination was assessed by checking the taxon of the best homolog for each predicted protein using a diamond search in the Uniref90 database which contains most of publicly available protein sequences clustered with 90% identity.

Relative transcription levels of protein-coding genes were calculated by mapping clean reads on the assembled transcripts using Trinity scripts with RSEM (Li and Dewey, 2011).

Functional annotation of the assembled transcripts was performed using NCBI-NR^3^, GO^4^, KEGG^5^, PlantTFDB^6^, MapMan Database^7^, and GeneMANIA^8^ (Warde-Farley et al., 2010). GO and KEGG terms were assigned using Trinotate v.3.1.1^9^ and KAAS annotation server (Moriya et al., 2007), respectively. GO and KEGG terms enrichment was analyzed with goseq v. 1.36.0 and clusterProfiler v.3.12.0, respectively, in R package (Young et al., 2010; Yu et al., 2012); terms with *p* < 0.05 were considered significantly enriched. UniProt data were used to characterize possible functions of translated products^10^. Volcano plots were produced using EnhancedVolcano v1.8.0 R package^11^. Venn diagrams were drawn using web server program^12^.

Differential expression was analyzed using Trinity scripts with the edgeR method; genes were considered differentially expressed if changes in the expression levels computed by EdgeR were not less than two-fold [| log_2_(FC)| ≥ 1], and false discovery rate (FDR) was ≤0.05.

Phylogenetic analysis was performed using the Fast Minimum Evolution method in NCBI^13^ and the Maximum Likelihood method based on the JTT matrix-based model in MEGA7.0 (Kumar et al., 2016).

### Validation of RNA-Seq Data

Real-time quantitative PCR results for 13 selected genes were validated with RT-qPCR. First-strand cDNA was synthesized with the Reverse Transcription System (Promega, Madison, WI, United States) using an oligo-dT and quantified by fluorimetry. RT-qPCR was performed with SYBR Green and ROX RT-PCR mixture (Syntol, Moscow, Russia), 3 ng cDNA, and 10 μM gene-specific primers in a CFX96 Real-Time PCR Detection System (Bio-Rad Laboratories, United States) at the following cycling conditions: initial denaturation at 95°C for 5 min, 40 cycles of denaturation at 95°C for 15 s and annealing/synthesis at 62°C for 50 s. As homologs of the two house-keeping genes, *Ubiquitin* (*UBQ*) and *Elongation Factor* (*ELF*) previously suggested for *N. khasiana* gene expression normalization (Dkhar and Pareek, 2019) did not show uniform expression, we normalized the data using two other reference genes, *UBQ-ligase Praja-2* and *Actin 7*. Experiments were carried out in three biological and three technical replicates and statistical analysis was performed using GraphPad Prism version 7.02 (GraphPad, San Diego, CA, United States^14^). P *≤* 0.05 was considered to indicate significant difference.

### Nucleotide Sequence Accession Numbers

The raw sequences obtained in this study have been deposited in the NCBI Sequence Read Archive under the accession numbers SRX5724495–SRX5724506.

## Results

### *Nepenthes* × *ventrata* Transcriptomes

**FIGURE 1:**
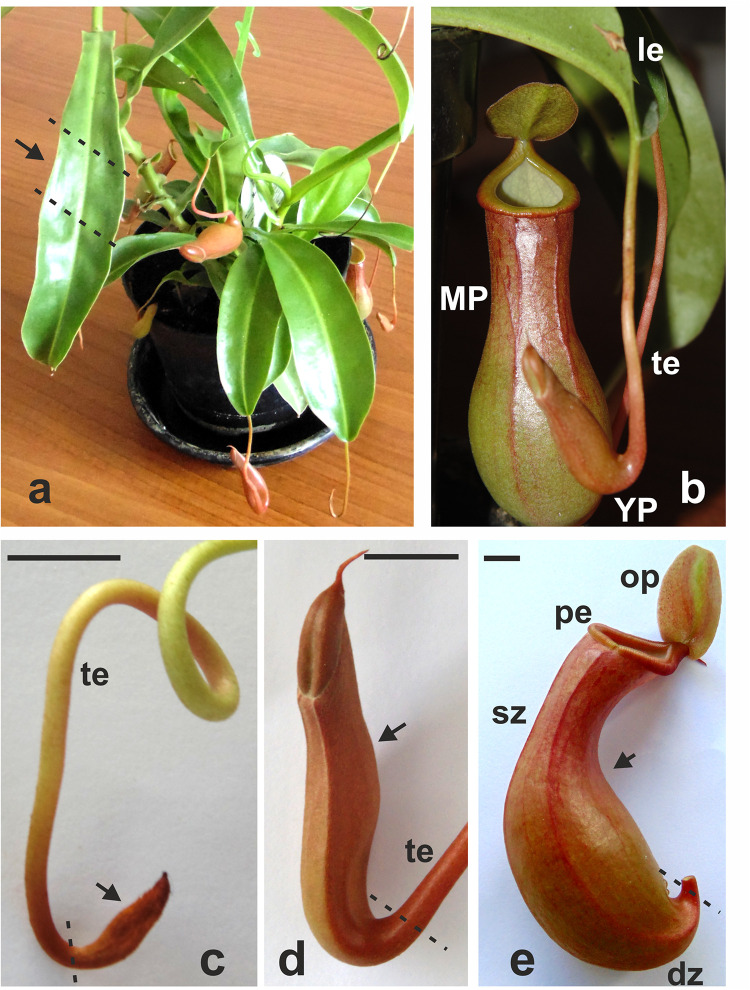
*Nepenthes* × *ventrata* whole plant **(a)**, the leaf with the tendril and the trap **(b)**, and early (primordial) **(c)**, young **(d)**, and late (mature open) **(e)** pitchers. Arrows indicates the part of the organ used for transcriptomics. dz, digestive zone; sz, slippery zone; pe, peristome (rim); op, operculum (lid); te, tendril; le, leaf; MP, late pitcher; YP, young pitcher. Dashed lines indicate organ parts taken for analysis. Scale bar = 1 cm.

Tissue samples of mature leaves and pitchers at three stages of development–early pitcher, young pitcher, and late pitcher (Figure 1) were analyzed by RNA-seq.

A total of 387,930,413 single-end reads (200 bp) and 23,277,185 paired-end reads (2 × 250 bp) were generated (Supplementary Table 1).

The reads of the four samples were combined and assembled. Overall, 245,999 transcripts (with size from 201 to 14,825 bp, total length 234.2 Mbp, and N50 size 1,602 bp) were generated. The completeness of the assembly was verified for 1,614 marker genes of the Embryophyta taxon using BUSCO, which revealed that 93.9 and 4.0% of the marker genes were complete and partial, respectively.

Annotation of the assembled transcripts using diamond search (cut-off *E*-value 10^–5^) against NCBI non-redundant database of plant protein-coding genes revealed that among 85,558 protein-coding sequences, 62,037 had significant homology to non-hypothetical plant proteins (Supplementary Table 2). The 85,558 sequences were used for further analysis.

We analyzed the taxonomy of the best homolog for each putative protein; only 135 proteins showed better homology with bacterial proteins. The total expression of these bacterial transcripts ranged from 0.10 to 0.24% depending on the samples; seven and one transcripts were identified as belonging to fungi and insects, respectively. Of the 53,400 proteins that matched in the Uniref90 database, 53,065 had plant proteins as the best homologs, which indicated an extremely low level of contamination.

Analysis with the Trinity software identified many groups of transcripts that may belong to the same gene or its isoforms, alternative splice variants, and paralogs, which could be explained by multiple paleopolyploidy events (at least seven genome duplications, according to Walker et al., 2017) in the evolutionary history of non-core Caryophyllales, including *Nepenthes*, as well as by the hybrid origin of *N.* × *ventrata*.

The *Nepenthes* × *ventrata* sequences showed the highest matches with *Vitis vinifera* (rosids; 12.38%), *Beta vulgaris* (11.12%), *Chenopodium quinoa* (10.77%), and *Spinacia oleracea* (Caryophyllales; 6.22%) sequences (Supplementary Table 3). According to the current taxonomy, *Nepenthes* (non-core Caryophyllales) should be closer to the asterid clade than to rosids (Yao et al., 2019); however, the top transcript homologs belonged primarily to rosids (60.52%), followed by Caryophyllales (28.17%) and asterids (6.36%) (Supplementary Table 3). This discrepancy may be attributed to a much higher number of assemblies for rosids (511) than for asterids and Caryophyllales (194 and 45, respectively) available in GenBank.

### Gene Ontology Annotation

Gene ontology classification of the transcripts according to the function of the translated products revealed that 4,827 of them belonged to Biological Process (BP), 2,752–to Molecular Function (MF), and 1,020–to Cellular Component (CC) categories representing 97 sub-aspects (Supplementary Tables 4, 5). The BP sub-aspects with the maximum number of transcripts were linked to stress response, cellular component organization, and biosynthetic processes (Supplementary Table 4). Most transcripts belonged to BP terms associated with general regulation of plant development, especially leaf development, senescence, and morphogenesis of leaves, and with photosynthesis, including carotenoid metabolism (Supplementary Table 5). The pitcher primordium, a red-colored organ with waxy inner walls, defines the efficiency of insect catching and processing; therefore, transcripts were classified in terms related to SAM development, biosynthesis of wax and anthocyanins, and plant-insect interactions. Of a particular interest are transcripts relevant to flowering initiation and formation of inflorescences and flowers, including reproductive organs, nectaries, pollen, ovules, and seeds (Supplementary Table 5).

The transcripts of the most enriched MF category were associated with utilization of ATP energy and the uptake and transfer of metal ions to specific locations, which is one of the basic requirements in photosynthetic plants (Yruela, 2013). The CC-classified transcripts were found to encode mainly nuclear, membrane, cytoplasmic, and plasma membrane proteins (Supplementary Table 5).

**FIGURE 2:**
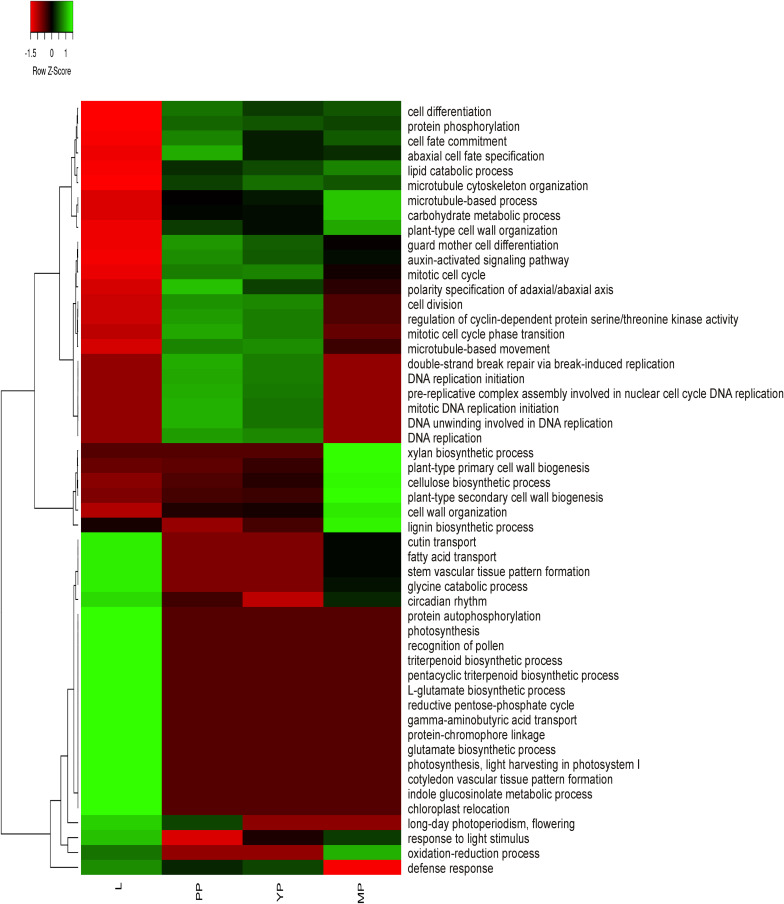
Heatmap based on relative number of ≥2 times upregulated transcripts in gene ontology (GO) terms in leaves (L) vs. early pitcher (PP) and in PP, young pitcher (YP), and late pitcher (MP) vs. leaves.

To obtain preliminary information about activation or suppression of various functions in leaves or pitchers, differentially expressed genes (DEGs) were identified based on the relative expression change of >2 times between the transcriptomes (Supplementary Table 6) and classified in GO terms (Supplementary Table 7). The most enriched BP/MF/CC terms in early and young pitchers vs. leaves were related to embryo development and active developmental and growth processes, whereas in late pitcher vs. leaves they were linked to plant-type cell wall biogenesis and organization, and in leaves vs. early/young/mature pitcher–to photosynthesis (Supplementary Table 7 and Supplementary Figure 1). Late pitcher was similar to leaves in the upregulation of genes involved in oxidation-reduction and downregulation of those related to DNA replication. Late pitcher differed from all other transcriptomes by the downregulation of defense response genes. Figure 2 shows transcriptional suppression of genes associated with photosynthesis and leaf developmental processes (early/young/mature pitcher), and induction of those involved in cell division, differentiation, and development of the growing (early/young pitcher) pitcher.

### Kyoto Encyclopedia of Genes and Genomes Annotation

Kyoto Encyclopedia of Genes and Genomes classification of transcripts in terms of KEGG PATHWAY (KP) and KEGG BRITE (KB; protein families) revealed 26,235 significant matches in the KP database, which were assigned to seven main classes (Supplementary Figure 2a), 45 sub-categories, and 401 pathways (Supplementary Table 8). Among the 26,235 matches, 15,204 were presented as 26 hierarchical classifications (incorporating many different types of relationships) in three sub-categories: “Genetic Information Processing,” “Signaling and Cellular Processes,” and “Protein families: Metabolism” (Supplementary Figure 2b and Supplementary Table 8). The presence of multi-transcript KP sub-categories “Environmental adaptation” and “Immune system” (Supplementary Figure 2c), as well as pathways “Plant hormone signal transduction,” “Plant-pathogen interaction,” and “MAPK signaling pathway–plant” (Supplementary Table 8) may reflect defense mechanisms and adaptation to nutrient deficiency in the carnivorous plant.

**FIGURE 3:**
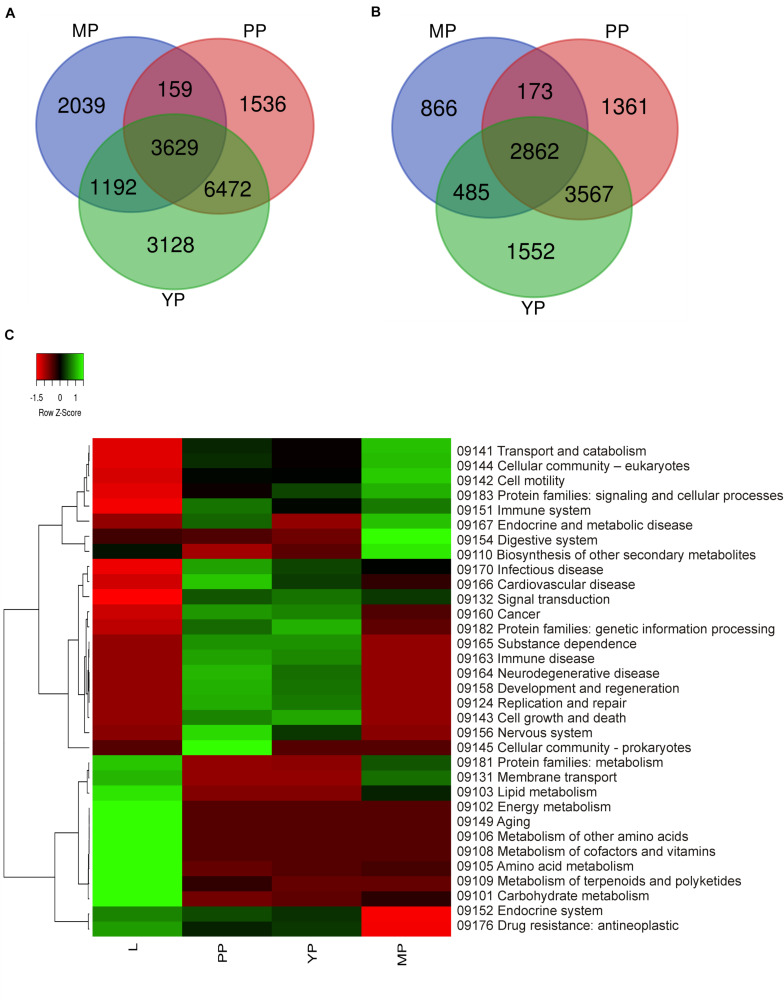
Comparison of differential expression of leaf and pitcher transcripts in Kyoto encyclopedia of genes and genomes (KEGG) terms. Number of transcripts upregulated **(A)** and downregulated **(B)** in early pitcher (PP), young pitcher (YP), and late pitcher (MP) vs. leaves (L) (Supplementary Table 8). Heatmap **(C)** based on relative expression of transcripts associated with KEGG terms (Supplementary Table 9) in leaves vs. early pitcher, and in PP, YP, and MP vs. leaves.

It should be mentioned that the plant data used by KEGG do not include characteristics of the carnivorous syndrome. Digestive fluid in *Nepenthes* pitcher contains proteases (such as nepenthesin), chitinases, glucanases, phosphatases, proteases, and pathogenesis-related proteins (Fukushima et al., 2017; Ravee et al., 2018; Pavlovic̀ and Mithöfer, 2019). In view of this, we briefly reviewed KP “Digestive system” and found that all our genes of interest were absent there and scattered throughout different other pathways such as “Amino sugar and nucleotide sugar metabolism,” “MAPK signaling pathway–plant,” “Plant hormone signal transduction,” “Plant-pathogen interaction,” etc. (Supplementary Table 8). Venn diagrams from KEGG gene ontology were used to visualize which tissues have similar gene expression and which are the most differentiated. Comparison of the number of activated and suppressed transcripts in KEGG categories showed the greatest similarity between early and young pitchers (Figures 3A,B).

Comparative analysis of KEGG terms enriched (by ≥2 times upregulated transcripts, Supplementary Table 6) in each transcriptome revealed that in leaves (vs. early pitcher), they were mostly associated with aging and primary and secondary metabolism, whereas in early and young pitcher (vs. leaves)–with genetic information processing, cell growth and death, development and regeneration, immune system, and signal transduction (Supplementary Table 9). Late pitcher was similar to leaves in the downregulation of genetic information processing, cell growth and development, immune system, and signal transduction, and to early/young pitcher–in the suppression of genes assigned to primary and secondary metabolism, and activation of transport and catabolism, immune system, and signaling (Supplementary Table 9 and Figure 3). Compared to the other transcriptomes, late pitcher showed transcriptional induction in the categories of “Digestive system” and “Biosynthesis of other secondary metabolites,” and reduction in the “Endocrine system” and “Drug resistance” (Supplementary Table 9 and Figure 3); the latter is possibly correlated with the downregulation of “Defense response” in GO enrichment analysis (Figure 2).

### Plant Transcription Factor Database Annotation

**FIGURE 4:**
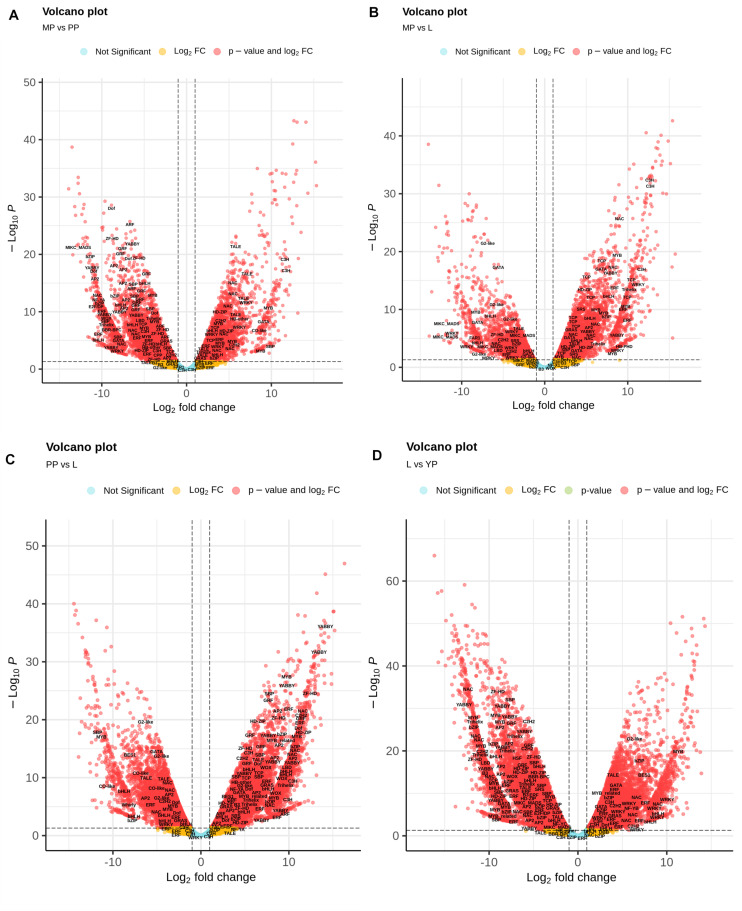
Volcano map analysis of DE transcripts in MP vs. PP **(A)**, in MP vs. L **(B)**, in PP vs. L **(C)**, and in L vs. YP **(D)**. TF-coding DETs are indicated.

*Nepenthes* transcripts encoding TFs were additionally characterized using plant-specific PlantTFDB. In total, 3,298 TF-encoding transcripts were classified into 48 families, among which the most represented (≥20 members) were MYB/MYB-related, bHLH, AP2/ERF, C2H2, C3H, bZIP, NAC, WRKY, G2-like, HD-ZIP, B3/ARF, GRAS, Dof, and MADS-domain protein families (Supplementary Table 10). Volcano plots were used to visualize the differences in expression levels between the transcripts, including TF-coding transcripts (Figure 4). Analysis of TF differential expression revealed similarity of the regulatory machinery between leaves and late pitcher and between early and young pitchers (Figure 4 and Supplementary Table 10).

### MapMan Annotation

**FIGURE 5:**
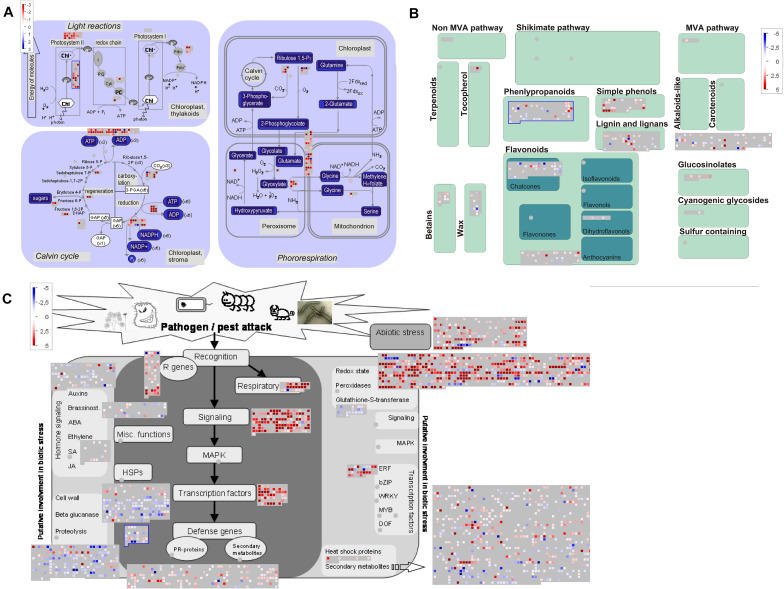
MapMan-based scheme showing differential expression of transcripts associated with photosynthesis **(A)**, secondary metabolism **(B)**, and stress responses **(C)** in PP compared to leaves. The activity of each pathway is presented as blocks, in which cubes represent individual transcripts and their colors reflect the difference in expression levels between the two transcriptomes. Transcripts upregulated in PP vs. leaves are indicated by blue in panel **(A)** and red in panel **(B,C)**.

Next, we used MapMan to facilitate comparison of upregulated transcripts (Supplementary Table 6) in early, young, and late pitchers vs. leaves, late and young pitchers vs. early pitcher, and late pitcher vs. young pitchers. Secondary metabolism patterns were similar between early pitcher and young pitchers and between leaves and late pitcher. Compared to leaves and late pitcher, early pitcher and young pitchers were found to have several secondary metabolism pathways upregulated, including biosynthesis of phenylpropanoids, anthocyanins, and alkaloids (Figure 5 and Supplementary Figure 3). Compared to leaves, photosynthesis was sharply reduced in pitchers at the early developmental stages (early and young pitchers) (Figure 5) but not at the late stage (mature pitcher) (Supplementary Figure 4). Genes related to biotic and abiotic stress responses were upregulated in pitchers at all developmental stages compared to leaves (Figure 5 and Supplementary Figure 5).

Overall, these results indicate a loss of leaf identity, i.e., photosynthetic activity, in the pitcher, and the upregulation of defense-related and metabolic processes during its development.

### Differentially Expressed Genes in Pitcher Initiation, Growth, and Maturation

Next, we selected DEGs upregulated >4 times and annotated them, using *Arabidopsis thaliana* UniProt data for the pair-wise comparisons of early, young or late pitchers with leaves, and leaves with early pitcher (Supplementary Table 11). The results revealed significant upregulation of transcripts related to flowering initiation and flower development, biosynthesis of secondary metabolites such as anthocyanins, terpenes, jasmonate, and wax, as well as stomatal development and movement, water transport, immunity, and stress response (Table 1).

**TABLE 1:** Selected differentially expressed genes (DEGs) upregulated > 4 times in leaves (vs. early pitcher), and early (PP), young (YP), or late (MP) pitchers vs. leaves.

**Proteins encoded by DEGs (function–according to UniProt)**	**L**	**PP**	**YP**	**MP**
**Negative regulation of vegetative-to-reproductive phase transition and flowering**				
TFIID subunit 14b (GAS 41-like)	**↑**			**↑**
EARLY BOLTING IN SHORT DAYS	**↑**	**↑**		
SPLAYED; RED AND FAR-RED INSENSITIVE 2	**↑**			
MADS-box protein SHORT VEGETATIVE PHASE (SVP)		**↑**	**↑**	**↑**
EARLY FLOWERING 1; EARLY FLOWERING MYB; and Homeobox protein ATH1		**↑**	**↑**	
**Prevention of the early vegetative-to-reproductive phase transition**				
CHROMATIN REMODELING 11			**↑**	
Homeobox-DDT domain protein RINGLET 3		**↑**		
Homeobox-DDT domain protein RINGLET 1	**↑**			
**Flowering promotion**				
AG-like MADS-box protein AGL19 (SOC1-like) and VASCULAR PLANT ONE-ZINC FINGER 1	**↑**			
AGL24-like; AtHB-31; VERNALIZATION 5; APETALA 2; FY; and FD		**↑**	**↑**	
AGL42-like; Flowering locus K; and FCA		**↑**		
**Flower, fruit, and seed development**				
AGAMOUS-like (AG)		**↑**	**↑**	**↑**
SEPALLATA 2-like (SEP2) and DEFICIENS-like (DEF, AP3)		**↑**		**↑**
FRUITFULL-like (FUL)/CAULIFLOWER (CAL, AP1)				**↑**
AGL6-like and AGL104-like		**↑**		
AG-like SEP1-like; SEP3-like; AGL61-like (DIANA); AGL37-like (PHERES1); AGL80-like; CMB1-like; JOINTLESS-like (J) AINTEGUMENTA (ANT); Auxin response factor 8 (FRUIT WITHOUT FERTILIZATION); and SPATULA (bHLH024)		**↑**	**↑**	
AGL65-like and AGL62-like			**↑**	
AGL11-like (SEEDSTICK and STK)	**↑**			
**Anthocyanin biosynthesis and accumulation**				
MYB1 (MYB113) and Anthocyanidin 5,3-*O*-glucosyltransferase (EC 2.4.1.)			**↑**	**↑**
LNK1 and Flavone 3’-*O*-methyltransferase 1 (EC 2.1.1.42)				**↑**
MYB3; BHLH42; GLABRA 3 (bHLH 001); ANTHOCYANINLESS 2; Flavonol synthase/flavanone 3-hydroxylase (EC 1.14.11.9) (EC 1.14.20.6) (FLS) and Anthocyanidin reductase [(2S)-flavan-3-ol-forming] (ANR) (EC 1.3.1.112)		**↑**	**↑**	
Chalcone-flavonone isomerase 3 (EC 5.5.1.6) (CHI)		**↑**		
Flavonoid 3’,5’-hydroxylase CYP75B138 (EC 1.14.14.81) (F3’5’H)	**↑**	**↑**	**↑**	**↑**
2-oxoglutarate-dependent dioxygenase At5g05600 (EC 1.14.11.) (ANS)	**↑**	**↑**	**↑**	
Chalcone synthase 2 (EC 2.3.1.74) (CHS); Naringenin,2-oxoglutarate 3-dioxygenase (EC 1.14.11.9) (FHT) (Flavanone-3-hydroxylase) (F3H); 2-oxoglutarate-dependent dioxygenase ANS (EC 1.14.11.-) (Anthocyanidin synthase); Dihydroflavonol 4-reductase (DFR) (EC 1.1.1.219) / (Flavanone 4-reductase) (FNR) (EC 1.1.1.234); Flavonoid 3’,5’-hydroxylase 1 (F3’5’H) (EC 1.14.14.81); Flavonol 3-*O*-glucosyltransferase UGT89B1 (EC 2.4.1.91); Leucoanthocyanidin reductase (EC 1.17.1.3); and Anthocyanidin 3-*O*-glucosyltransferase (EC 2.4.1.115) (UFGT)		**↑**	**↑**	**↑**
**Digestive enzymes**				
Aspartic proteinase nepenthesin-2 (EC 3.4.23.12)	**↑**			**↑**
Aspartic proteinases nepenthesin-1 (EC 3.4.23.-) and PCS1; Chitinase-like protein 1 (AtCTL1)		**↑**	**↑**	**↑**
Aspartyl protease family protein At5g10770 and Cysteine protease RD21A			**↑**	**↑**
Aspartic proteinase A2 (Aspartic protease 57); Aspartic proteinase-like protein 2; and Cysteine proteases RD19A, RD19B (EC 3.4.22.-)				**↑**
**Organization of SAM during embryogenesis and organ separation**				
WUSCHEL-related homeobox (WOX) 1; WOX2; WOX3; WOX4; YABBY1; ASYMMETRIC LEAVES 2 (AS2); MAINTENANCE OF MERISTEMS; FASCIATA1; FASCIATA2; TORNADO2; CUP-SHAPED COTYLEDON 2 (CUC2); CUC3; TEBICHI; PHABULOSA (PHB, ATHB14); KANADI 2 (KAN2); and GROWTH-REGULATING FACTOR (GRF) 1–4, 6–9		**↑**	**↑**	
AS2-like proteins 1 and 38; WOX9; and DEK1		**↑**		
WOX8 and WOX13				**↑**
YABBY4			**↑**	
YABBY2; YABBY5; and ASYMMETRIC LEAVES 1 (PHANTASTICA)		**↑**	**↑**	**↑**
REVOLUTA (REV); ERECTA (ER); ER-like kinase 1 (ERL1); TONSOKU; SEMI-ROLLED LEAF 2 (SRL2); and GRF8		**↑**	**↑**	**↑**

In accordance with pitcher initiation on the tendril apical meristem, early pitcher differed from leaves in the activation of processes associated with the initiation and organization of embryonic SAM, and determination of organ abaxial–adaxial polarity (Table 1 and Supplementary Table 11).

Initiation of the pitcher primordium was accompanied by transcriptional activation of genes associated with the redox state, abiotic stress, respiration, and signaling as well as TFs and R-genes (Figure 5 and Supplementary Figures 3–5). Early pitcher was similar to young pitcher but both differed from late pitcher in the expression pattern of genes regulating the redox state and induction of signaling, respiration, and TF activity (especially ethylene-responsive factors) (Supplementary Figure 5 and Supplementary Table 11). The upregulated TFs were: ERFs (ERF1A, RAP2-1, ERF012, ERF061, and WRINKLED 1), ANAC081, ANAC022, ANAC042, MYB2, MYB73, MYB88, ATAF2, ZAT5, ZAT9, HSF24, and WRKY 70, etc. (leaves vs. early pitcher); ERFs (AIL1, AIL5, CRF2, CRF4, ESR2, TINY 2, WIN1, and ERF003), heat shock factors (HsfA2, A3, A7, B2, and B4), ANAC083, MYB88, bHLH98, etc. (early pitcher vs. leaves); ERFs (TINY 2, ERF023, ERF115, SHINE 2, WIN1, and ERF003), bZIP36, MYB88, and ZAT9, etc. (mature pitcher vs. leaves) (Supplementary Table 11).

Furthermore, initiated and developing pitchers showed upregulation of stress-response factors encoding pathogenesis-related proteins (PR-1C, Fra a 1.03, PRHA, STH-2, STH-21, PTI5, PRHA, SNI1, and thaumatin-like), LRR receptor-like serine/threonine protein kinases, digestive enzymes (peroxidases, glucanases, alfa- and beta-amylases, phospholipases, chitinases, and aspartic proteinases nepenthesin-1 and -2), ABC transporters, dehydration-responsive elements, heat shock proteins, and jasmonate signaling-related molecules (Supplementary Tables 2, 6, 11).

Pitchers at different developmental stages also showed upregulation (>2–4 times) of genes related to stomatal and guard cell development and functioning, including those involved in water and CO_2_ transport across cell membranes, such as aquaporins (NIP5-1, PIP1-2, PIP2-2, PIP2-7, NIP1-2, and TIP1-2 in late pitcher; PIP1-1 and SIP1-2 in early pitcher and YP; PIP1-3 and PIP1-4 in early and young pitchers; and PIP2-8, SIP1-1, TIP1-1, TIP1-3, and TIP4-1 in pitchers of all stages) and aquaporin level regulators (E3 ubiquitin-protein ligase RMA1H1 in late pitcher), and reversible hydration of CO_2_ (carbonic anhydrase in leaves, early pitcher, and late pitcher), as well as genes encoding formate dehydrogenase and ribulose 1,5-bisphosphate carboxylase (in late pitcher) (Supplementary Tables 2, 6, 11).

Although some genes associated with stomatal patterning were upregulated in leaves (such as those encoding calcium sensing receptor, guard cell S-type anion channel SLAC1, and TFs MYB88, bHLH97, and WRKY70), many more of such genes were induced in pitchers, including microtubule-associated protein 1, ABA receptor PYL9, alkaline ceramidase, CO_2_-response secreted protease, respiratory burst oxidase homolog protein F, EPIDERMAL PATTERNING FACTOR-like protein 3, ER, ERL1, STOMATAL DENSITY AND DISTRIBUTION 1, and TFs bHLH93, bHLH1, bHLH98, MYB61, MYB66, MYB88, and others (Supplementary Table 11).

Pitchers were also characterized by activation of the terpenoid pathway. Many relevant genes were upregulated in the primordium, including those encoding enzymes involved in terpenoid biosynthesis, such as isopentenyl phosphate kinase, (E,E)-alpha-farnesene synthase, terpene synthase 8, GDSL lipase, tabersonine-19-hydroxy-*O*-acetyltransferase, and sesquiterpene synthase STPS (Supplementary Table 11). In MPs with developed glandular tissues, the upregulated transcripts encoded TFs (WRKY 72A and EXPRESSION OF TERPENOIDS 1) and enzymes involved in biosynthesis of germacrene D, germacrene-derived sesquiterpene lactones, triterpene saponins and phytosterols, and monoterpenoid indole alkaloids (Supplementary Table 11).

Among the TFs upregulated in early pitcher vs. leaves by more than two times there were multiple homologs of many flowering-related MADS-domain TFs (Table 1 and Supplementary Tables 10, 11). We performed phylogenetic analysis of the found 45 proteins in comparison with the known MADS-TFs of a model plant *A. thaliana* and a representative of Caryophyllales, *B. vulgaris*; in addition, each of the 45 proteins was separately analyzed with the most similar sequences available from the NCBI GenBank. As a result, the *NveMADS1-45* genes were classified as homologs of *AGL24*, *AGL42*, *SVP*, *SOC1*, *AP1*/*FUL*, *SEP1*/*SEP2*, *SEP3*, *SEP4*, *AG*, *STK*, *AP3*, *AGL6*, *AGL62*, *AGL65*, *AGL104*, *AGL80*, and *AGL82* (Supplementary Table 12 and Supplementary Figure 6).

To verify the presence of the MADS-box transcripts in non-flowering *N.* × *ventrata* tissues (developing pitcher), we searched available transcriptome data on *N.* × *ventrata* late pitcher (24 h after opening) (Wan Zakaria et al., 2016) (NCBI TSA: *Nepenthes ventricosa* × *Nepenthes alata*, transcriptome shotgun assembly; GFAD00000000.1) and male inflorescence of another relative species *N. khasiana* (Scharmann et al., 2019). As a result, we found homologs of *AGL24*, *AGL42*, *SVP*, *SOC1*, *FUL*, *AP1*, *SEP1*, *SEP2*, *SEP3*, *SEP4*, *AG*, *STK*, *AP3*, *AGL6*, *AGL14*, *AGL16*, *AGL19*, *AGL37*, *AGL61*, *AGL66*, *AGL80*, and *AGL104* genes in the *N.* × *ventrata* pitcher transcriptome, which confirms the reliability of the data obtained in this study. The *N. khasiana* male inflorescence transcriptome contained homologs of *SOC1*, *SVP*, *AP1*, *SEP*, *JOINTLESS*, *AG*, B-class genes, *AGL29*, *AGL42*, *AGL61*, *AGL62*, *AGL65*, *AGL80*, *AGL82*, *AGL104*, and *MADS6* (Supplementary Table 13).

The identified *N.* × *ventrata* MADS-box genes are known to be involved in plant reproductive development and are expressed in *Arabidopsis* reproductive organs (except for *SVP*) (Smaczniak et al., 2012; Theißen et al., 2016). *AGL24*, *AGL42*, *SVP*, and *SOC1* are also expressed in *Arabidopsis* roots (Parenicová et al., 2003). In addition, *AGL24*, *AGL42*, *SVP*, *SOC1*, *SEP1*, *SEP4*, *AGL6*, *AGL62*, *AGL65*, and *AGL80* are expressed in the vegetative parts of the plant; among them, *AGL24*, *AGL42*, *SVP*, and *SOC1* are associated with flowering initiation (Parenicová et al., 2003; Dorca-Fornell et al., 2011; Hu et al., 2014; Valentim et al., 2015). Thus, the presence of the listed MADS-box genes (like *AP1*, *SEP3*, *AG*, *AGL11*, and *AP3*) in supposedly vegetative *N.* × *ventrata* pitchers and leaves is unusual and deserves special attention.

According to GeneMANIA prediction, *N.* × *ventrata* homologs of type I proteins (AGL65 and AGL104) can participate in gametophyte development, whereas homologs of type II proteins–in the regulation of shoot system development (FUL, AGL6, AGL42, SOC1, and SVP), positive (FUL, AGL6, AGL42, and SOC1) and negative (SVP) regulation of reproductive development, regulation of floral and post-embryonic organ development (AGL11, AGL42, AG, SEP3, and AGL6), and meristem identity and stem cell development and differentiation (FUL, AGL24, AP1, SOC1, and SVP) (Supplementary Tables 14,15).

**FIGURE 6:**
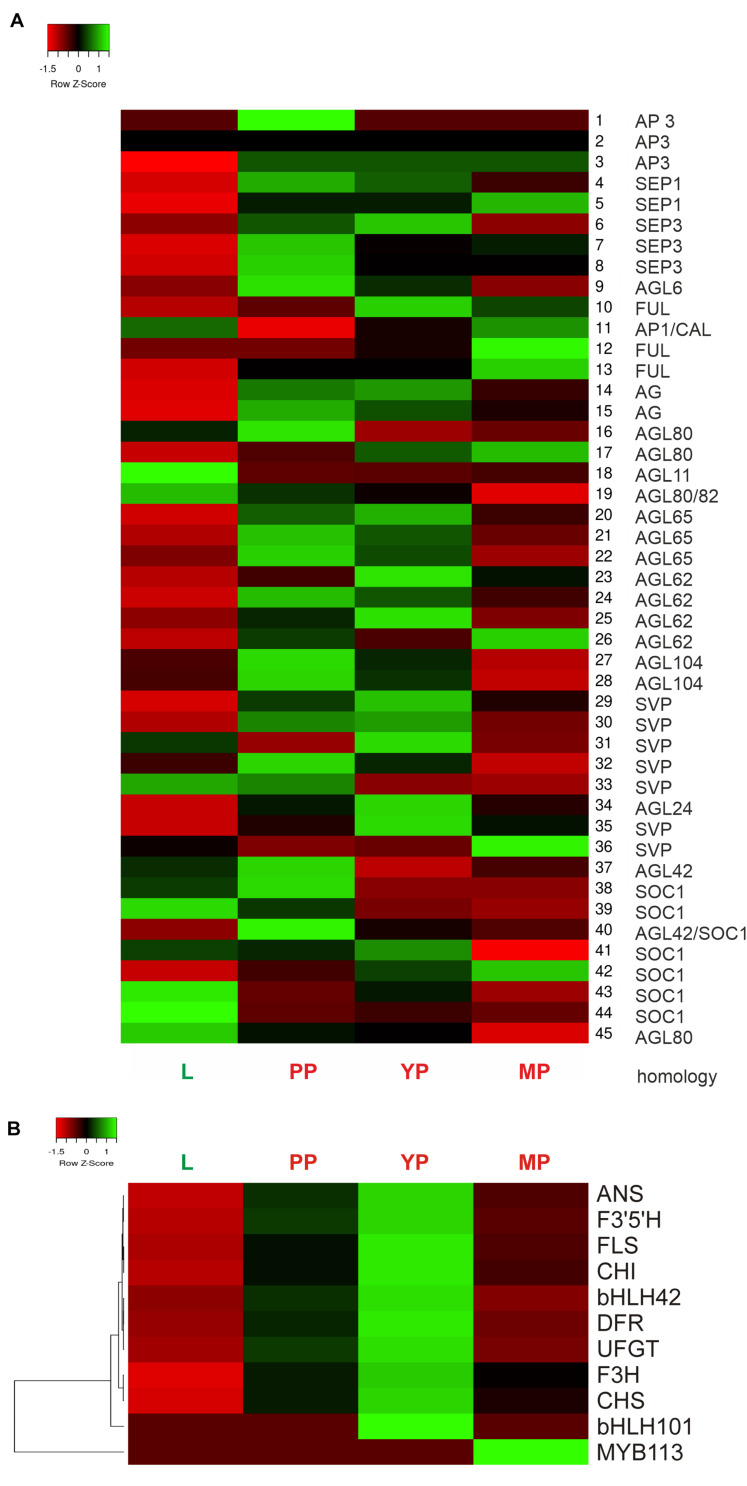
Heatmap of relative expression of the MADS-box genes *NveMADS1–45* **(A)** and genes involved in anthocyanin biosynthesis **(B)**. L vs. PP, and PP, YP and MP vs. leaves.

Heatmap shows activation of most MADS-box genes during pitcher initiation and development (Figure 6A). In early and young pitchers vs. leaves/late pitcher, the *NveMADS11* (AP1/FUL clade), *NveMADS19* and *NveMADS45* (AGL80), *NveMADS31* (SVP), *NveMADS36* (AGL24), and *NveMADS39*, *43*, and *44* (SOC1) genes were downregulated, whereas the remaining genes were upregulated (Figure 6A). In early pitcher vs. leaves, transcript isoforms homologous to *SOC1* (*NveMADS39*, *43*, *44* / *NveMADS37*, *38*, *40*, *41*, *42*), *SVP* (*NveMADS29-30, 32*, *33* / *NveMADS31*, *36*), and *AP1*/*FUL* (*NveMADS10, 12, 13* / *NveMADS11*) were, respectively, up-/downregulated (Figure 6A).

Consistent with the red color acquired by developing pitchers, we observed activation of anthocyanin biosynthesis in early and young pitchers, which is evidenced by the upregulation of many relevant enzymes and TFs (Table 1 and Supplementary Tables 10,11). Compared to leaves, all pitcher stages showed activation of most of the analyzed genes for anthocyanin biosynthesis with a maximum transcription level in young pitcher; one exception was *MYB113* whose expression was constant in leaves, early pitcher, and young pitcher, but increased sharply in late pitcher (Figure 6B). Furthermore, late pitcher showed a two-fold upregulation of the gene encoding 4,5-DOPA-extradiol dioxygenase (Supplementary Table 6), one of the main enzymes involved in the biosynthesis of betalains–a class of red and yellow pigments (Timoneda et al., 2019).

Regarding other important pigments, carotenoids, it should be mentioned that very few genes of the carotenoid pathway, associated with fruit-specific sequestration of pigment in chromoplasts and abscisic acid synthesis, rather than photosynthesis and photoprotection, were found upregulated: chromoplast-specific carotenoid-associated protein CHRC (in leaves and late pitcher vs. early pitcher), 9-*cis-*epoxycarotenoid dioxygenase NCED1 (in leaves vs. late pitcher and in early pitcher vs. leaves), and carotenoid-cleavage dioxygenase NCED4 (in late pitcher vs. leaves and early pitcher) (Supplementary Table 11).

### Validation of RNA-Seq Data by Real-Time Quantitative PCR

Thirteen DEGs were selected for RT-qPCR validation of the transcriptomic data with specific primers (Supplementary Table 16): 10 genes of the anthocyanin biosynthetic pathway (regulatory *MYB113* and *bHLH001* and structural *CHS1*, *CHS2, CHI*, *F3H*, *F3’5’H*, *DFR*, *ANS*, and *UFGT*) and three TF genes associated with flowering time and floral organ identity (*MYB17* and MADS-box genes *AG* and *SEP3*). The results showed significant upregulation of all analyzed genes in early pitcher compared to leaves, with the exception of *MYB113* activated in late pitcher (Supplementary Figure 7), which was consistent with the transcriptome data (Figure 6).

## Discussion

The *Nepenthes* pitcher, derived from the tip of the leaf tendril, is thought to emerge from a spontaneously occurred epiascidiate leaf folded into a tubular structure (outer adaxial side inward) with fused margins, which provided selective advantage through improved storage of water and nutrients (Givnish, 2015). To survive in nitrogen-deficient conditions, *Nepenthes* (like other known carnivorous species with similar trap structures) turned the tubular leaf into a trap-pitcher with prey attraction and digestion abilities (Givnish, 2015).

### Stress-Response Proteins Trigger Trap Initiation

Comparative genomic and transcriptomic studies of true leaves and leaf traps in different carnivorous species, including *Nepenthes*, have shown that signaling pathways involved in prey catching and digestion are similar to those providing protection against pathogens in non-predatory plants (Renner and Specht, 2013; Fukushima et al., 2017). For example, jasmonates associated with response to stress, microbial pathogens, and pests in non-carnivorous plants, in carnivores can perceive signals from captured prey and participate in the development of digestive cavities loaded with hydrolytic enzymes (Pavlovic̀ and Mithöfer, 2019), including pathogenesis-related proteins associated with stress response, which acquired digestive properties (Pavlovic̀ and Mithöfer, 2019). These data suggest that carnivorous plants evolved using pest defense mechanisms (Fukushima et al., 2017; Pavlovic̀ and Mithöfer, 2019). The results of this study revealed that genes related to stress response and pathogen/pest attacks were upregulated in the early pitcher transcriptome (Supplementary Tables 6, 11, Figure 5, and Supplementary Figures 3–5), which is consistent with the hypothesis of defense-to-carnivory pitcher evolution.

### Pitcher Primordium Organization

In response to defense-related signals, the central vein of the leaf lengthens, producing a tendril with an apical meristem at the tip, which forms the pitcher in the process directed by phyllotaxis signals that promote cell division activity and prevent premature cell differentiation. We found that transcription of the *WOX* genes was upregulated in pitchers compared to leaves (Table 1, Supplementary Table 11, and Figure 4), which may be related to lateral organ formation (*WOX1* and *WOX3*), embryo patterning (*WOX8* and *WOX9*), floral transition (*WOX13*), and determination of vascular stem cell niche (*WOX4*) (van der Graaff et al., 2009; Zhou et al., 2015).

Consistent with cell division patterns in adaxial tissues shown in carnivorous *Sarracenia purpurea* (Fukushima et al., 2015), in *N.* × *ventrata* early pitcher the genes regulating the abaxial–adaxial polarity and initiation/organization of embryonic SAM were activated (Table 1, Supplementary Table 11, and Figure 4), including abaxial–adaxial polarity genes shown to be upregulated in pitcher-bearing shoots of carnivorous *Cephalotus* (Fukushima et al., 2017), as well as *AS1* and *REV* genes activated at the site of *N. khasiana* trap initiation and formation (Dkhar and Pareek, 2019). *KAN* and *PHB* genes upregulated in the *N.* × *ventrata* pitcher may determine its initial asymmetric growth and *YABBY* genes may specify the fate of abaxial cells, thus contributing to the proper abaxial/adaxial organization of the trap as shown in *Arabidopsis* (Eshed et al., 2004). Also similar to *Arabidopsis* (Inagaki et al., 2006; Rast and Simon, 2012; Shpak, 2013; Amanda et al., 2016), the longitudinal growth of *N.* × *ventrata* trap primordium may depend on the mutual activity of the upregulated *ER*, *ERL1*, and *ERL2* genes, whereas cell division and differentiation (including epidermal cells) may rely on *TEBICHI* and *DEK1*, and symmetry–on *AS2*. *GRF* genes are known to be involved in the control of cell expansion in the leaf (Dkhar and Pareek, 2014) and, hence, may be important for *N.* × *ventrata* trap outgrowth at the tip of the leaf.

Folding of the tubular trap may require *SRL2*, which regulate leaf rolling through abaxial cell differentiation (Liu et al., 2016), as well as *bHLH30* involved in upward leaf curling (An et al., 2014) and *FIL* and *YABBY2* related to the twisted leaf phenotype (Bowman, 2000).

Overall, the activation and cooperative activity of these genes in *N.* × *ventrata* early pitcher may contribute to the epiascidiate leaf folding.

### Prey Capture

The pitcher of *N. alata* (an *N.* × *ventrata* parent) catches the prey with the help of anisotropic slippery, highly wet peristome (with water and/or nectar films), waxy inner walls, and release of attractants and sugar-rich nectar (Bohn and Federle, 2004; Bauer et al., 2008). The captured prey is then digested in the lower zone fluid enriched with hydrolytic enzymes (Bauer et al., 2008). Consistent with these data, in *N.* × *ventrata* pitcher transcriptomes we observed activation of genes involved in sucrose, wax/cutin, and alkaloid biosynthesis, as well as those encoding proteases and oligosaccharide hydrolases (Supplementary Table 11 and Figure 5).

To attract insects with well-developed CO_2_ receptors, closed *Nepenthes* pitchers accumulate CO_2_ emitted after opening, which decreases pitcher photosynthetic capacity, while promoting its growth and respiration and increasing carbohydrate synthesis, cuticular wax density, and humidity levels (Baby et al., 2017). Moreover, CO_2_ dissolved in the digestive fluid maintains the optimum pH for the activity of hydrolytic enzymes and nutrient absorbance (Baby et al., 2017). Because of the high CO_2_ concentrations, “modified stomata” evolved on the inner side wax layers of *Nepenthes* pitchers have altered morphology (Baby et al., 2017). The transcriptome data obtained in this study confirmed the role of CO_2_ in the development and functioning of the *N.* × *ventrata* pitcher, as evidenced by the upregulation of genes related to reversible CO_2_ hydration and fixation, CO_2_ and water transport during transpiration, and stomatal and guard cell development and function (Supplementary Table 11).

Traps produced by *Nepenthes* plants have low or no photosynthetic activity and, consequently, lower content of chlorophylls and carotenoids but higher content of anthocyanins (Pavlovič and Saganová, 2015). Accordingly, our comparative transcriptome analysis revealed downregulation of photosynthesis-related genes and upregulation of anthocyanin pathway genes in *N.* × *ventrata* pitchers at all stages compared to leaves (Supplementary Table 11 and Figures 5, 6B). Another recent study of *Nepenthes* pitchers (Goh et al., 2020) also showed the downregulation of genes associated with photosynthesis and increased biosynthesis of secondary metabolites in a pitcher depleted of fluid proteins.

### Anthocyanin Pathway in Prey Attraction

Carnivorous plants, including *Nepenthes* spp., use insects both as pollinators and as food, participating in pollinator-prey conflict (Jürgens et al., 2012). *Nepenthes* pitchers, which do not or weakly reflect UV radiation visible to insects (Schaefer and Ruxton, 2008), acquire the same red color in pitchers and flower tepals by increasing anthocyanin production (Scharmann et al., 2019).

Red coloration could have initially developed in carnivorous plants as an adaptive trait, since anthocyanin accumulation is often associated with stress responses or nutritional deficiency in plants (Schaefer and Ruxton, 2008). At the same time, it increased prey capture efficiency of the traps by providing attractive visual signals. Capture rates are positively correlated with levels of red pigmentation, probably because insects can detect differences in red light intensity compared to the green background (Schaefer and Ruxton, 2008).

The anthocyanin pathway is activated by the MBW complex of TFs, including R2R3-MYB, bHLH, and WD40, which regulate sequential steps in the synthesis of naringenin chalcone, naringenin, and dihydrokaempferol catalyzed by chalcone synthase, chalcone isomerase, and flavanone 3-hydroxylase, respectively (Naing and Kim, 2018). The pathway is then divided into three branches, which ultimately produce three types of anthocyanins (derivatives of cyanidin, delphinidin, and pelargonidin of different colors) as a result of three sequential reactions catalyzed by dihydroflavonol-4-reductase (DFR), anthocyanidin synthase (ANS), and UDP-glucosoflavonoid-3-*O*-glucosyltransferase (UFGT), respectively (Naing and Kim, 2018).

**FIGURE 7:**
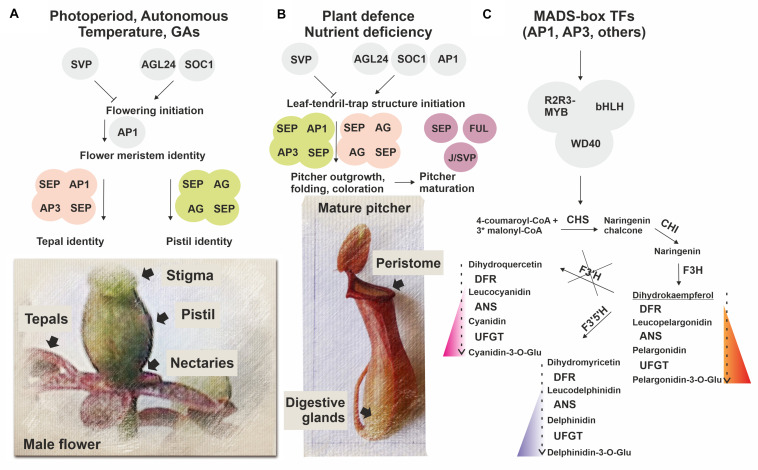
Simplified scheme illustrating a possible role of MADS-domain TFs in *N. × ventrata* flower **(A)** and pitcher (leaf-tendril-trap) **(B)** initiation and specification, including anthocyanin pigmentation **(C)**. Homologs of MADS-domain TFs SVP, AGL24, SOC1, and AP1 involved in flowering initiation possibly determine the time of pitcher initiation, whereas homologs of MADS-domain TFs SEP1, 3, AP1, FUL, AP3, AG, and J/SVP involved in the control of floral organ identity and fruit development may also be implicated in conferring pitcher identity signatures. At the same time, MADS-domain TFs may regulate activation of the anthocyanin pathway in developing flowers and pitchers.

We found that in *N.* × *ventrata* red-burgundy pitchers, most of the anthocyanin pathway genes identified in *Arabidopsis* were expressed, except for *F3’H*, whose transcripts were not detected (Supplementary Tables 6, 11, Figures 5, 6B, and Supplementary Figure 6). *F3’H* encodes flavonoid 3*’*-hydroxylase catalyzing conversion of dihydrokaempferol to dihydroquercetin and its absence may suggest that *N.* × *ventrata* lacks the branch of cyanidin synthesis. Products of the other two branches are blue delphinidins and orange–red pelargonidins (Tanaka and Ohmiya, 2008) and, considering *N.* × *ventrata* pitcher color, we suggest that biosynthesis of pelargonidin may be the most pronounced branch of the anthocyanin pathway in *N.* × *ventrata* pitchers (Figure 7C).

In some families of core Caryophyllales, flowers and fruits produce betalains: red-violet betacyanins and yellow betaxanthins, which are structurally and biochemically unrelated to anthocyanins and exclude their synthesis (Timoneda et al., 2019). Nepenthaceae are noncore Caryophyllales that produce anthocyanins (Timoneda et al., 2019); nevertheless, the gene encoding a main enzyme of the betalain pathway, 4,5-DOPA-extradiol-dioxygenase (DODA), was found to be activated in *N.* × *ventrata*, late pitcher (Supplementary Table 6). DODA catalyzes the conversion of 3,4-dihydroxy-L-phenyalanine (L-DOPA) to 4,5-seco-DOPA, which spontaneously forms betalamic acid, the precursor of all betalain compounds. Spontaneous conjugation of betalamic acid with cyclo-DOPA produced by cyclization of oxidized L-DOPA generates red-violet betacyanins (Polturak and Aharoni, 2018). Along with DODA activation, transcription of anthocyanin biosynthesis genes was decreased in late pitcher compared to young pitcher (Figure 6B), which confirms mutual exclusion of anthocyanins and betalains (Timoneda et al., 2019) and indicate a possibility for betalain biosynthesis at later developmental stages of noncore Caryophyllales.

### MADS-Domain TFs as Possible Regulators of Pitcher Origin and Development

As part of a long-lasting discussion about the origin of the carnivorous syndrome in plants, Charles Darwin, already in the 19th century, pointed out the similarities between traps and reproductive parts of the flower (stamens and pistils), but since that time, there has been no experimental evidence to support this possibility (Pennisi, 2002). In this study, the MADS-domain TFs (Table 1 and Supplementary Tables 2, 6, 11, 12), which play decisive roles in plant reproductive development (Smaczniak et al., 2012; Theißen et al., 2016) (Supplementary Tables 14, 15), were revealed as the most intriguing set of transcripts upregulated in *N.* × *ventrata* pitchers. In higher plants, MADS-domain TFs regulate flowering initiation (*AGL24*, *AGL42*, *SOC1*, and *AP1*) and repression (*SVP*) (Dorca-Fornell et al., 2011; Hu et al., 2014; Valentim et al., 2015). We found that *AGL24*, two of the four *AP1*/*FUL* homologous genes, and six of the eight *SOC1*/*AGL42* homologous genes were upregulated, whereas three of seven *SVP* homologous genes were downregulated in early pitcher vs. leaf (Figure 6A). We also observed activation of genes from other gene families (*FY*, *FCA*, *FLK*, *FD*, and *CONSTANS-like*) known to control flowering time through regulation of MADS-box gene transcription (Table 1 and Supplementary Tables 6, 11). In *N.* × *ventrata* vegetative SAM, *AGL24*, *AGL42*, and *SOC1* encode proteins which may induce the expression of inflorescence meristem identity gene *LEAFY* (*LFY*) that in turn may activate floral meristem identity gene *AP1* as shown for *Arabidopsis* (Smaczniak et al., 2012). We found all these genes, with the exception of *LFY*, both in the leaves and pitchers (Table 1 and Supplementary Table 12), suggesting that the expression of these genes is independent of the flowering process and that the single leaf-tendril-trap unit may represent a novel structure (adapted for plant nutrition), as has been shown for grapevine tendrils adapted for plant climbing (Calonje et al., 2004). Transcripts of all four genes homologous to *AP1*/*FUL* were found in all types of transcriptomes (Supplementary Table 12), which supports the idea that the leaf, tendril, and trap represent a single structure. In this case, *LFY* could be transcribed in the meristem prior to the initiation of leaf-tendril-trap differentiation.

*Nepenthes* × *ventrata* pitchers were also characterized by the upregulation of MADS-box genes *AG*, *AP3*, *SEP1*, *SEP3*, *SEP4*, and *AGL6* shown to be responsible for floral organ identity (Theißen et al., 2016), as well as genes involved in the development of fruit (*FUL*, and *J*/*SVP*), pollen (*AGL65*, and *AGL104*), and male gametophytes (*AGL80*, and *AGL62*) (Verelst et al., 2007; Nakano et al., 2015; Figueiredo et al., 2016; Toh et al., 2018; Batista et al., 2019) (Table 1, Supplementary Tables 12, 14, 15, and Figure 6A).

According to the ABCDE and Quartet models, floral organs are defined by various combinations of MADS-domain TFs (A, B, C, D, or E-class) assembled into quaternary complexes (Smaczniak et al., 2012). A + E (AP1 + SEP) specify the identity of flower meristem and sepals, A + B + E (AP1 + AP3/PISTILLATA (PI) + SEP)–petals, B + C + E (AP3/PI + AG+SEP)–stamens, C + E (AG + SEP)–carpels (forming pistil), and C + E + D (AG + SEP + STK)–ovules (Theißen et al., 2016). In *N.* × *ventrata* early pitcher and young pitcher, all ABCDE genes, except suppressed *STK* and absent *PI*, were upregulated compared to the leaf; the leaf transcriptome contained all these genes, except *AGL6* (Supplementary Table 12).

Duplication, diversification, and neo-functionalization of the MADS-box genes are thought to underlie the origin of flower organs through leaf modification (Melzer et al., 2010; Theißen et al., 2016). As a result, highly conserved MADS-domain TFs in various combinations represent a mechanism controlling flower meristem differentiation (Theißen et al., 2016). The overwhelming variety of existing flower forms indicates the ongoing evolution of the flower MADS-box set through selective changes in protein-protein interactions, downstream target genes, and regulatory patterns (Bartlett, 2017).

Besides traps, dioecious *Nepenthes* species form female and male flowers on separate plants and, therefore, must have a complete set of highly conserved MADS-box genes associated with flowering (Subramanyam and Narayana, 1971). Indeed, the *N. khasiana* male inflorescence transcriptome (Scharmann et al., 2019) contained the similar set of the MADS-box genes as *N.* × *ventrata* leaves and pitchers (Supplementary Tables 2, 12).

It is worth noting the mimicry of pitchers as flowers in the patterns of scent emission (Di Giusto et al., 2010) and the similarity of the pitcher with a petal in color and stomatal patterning and that of the pistil with a tubular structure, extrafloral nectaries in the peristome, and lower glands at the base inside the trap, which produce digestive fluid (Gaume and Forterre, 2007; Bauer et al., 2008). In many plant species, the surface of the carpel stigma is often uneven, tuberous, and wet because of the wax cover and sugar-rich sticky exudate, which contribute to more efficient pollen adhesion (Lau et al., 2017). The peristome also has an anisotropic surface coated with a film of water and/or attractive and slippery sugar-rich nectar for prey capture (Bohn and Federle, 2004).

It is postulated that the carpels, as well as other floral organs, emerged as a result of leaf modifications, which is confirmed by the inter-conversion between leaves and flower organs when the A, B, and C classes or E-class of MADS-box genes are overexpressed or inactivated (Scutt et al., 2006).

We speculate that *N.* × *ventrata* trap development may pass through several transitional steps: (i) initiation of a leaf-tendril-trap structure due to the cooperative activity of flowering time-controlling genes *AGL24* (*NveMADS34*), *AGL42*/*SOC1* (*NveMADS37–44*), *SVP* (for example, isoforms *NveMADS32*, *33*), and *AP1* (*NveMADS11*); (ii) outgrowth, folding, and coloration of the pitcher structure [upregulation of floral organ identity-related genes *AP1* (*NveMADS11*), *AG* (*NveMADS14*, *15*), *SEP*s (*NveMADS4, 6–8*), and *AP3* (*NveMADS1–3*)]; (iii) maturation of the tubular structure acquiring differentiated pistil-like attributes, and upregulation of *SEP*s (*NveMADS5*), *FUL* (*NveMADS10*, *12*, *13*) and *J*/*SVP* (for example, isoforms *NveMADS31*, *36*) potentially involved in pitcher ripening. The functions of type I flower-specific MADS-box genes commonly involved in pollen, and male gametophyte development are unclear; they are less studied than type II genes and may have some unknown roles in the development of both flowers and pitchers.

Besides flower specification, flowering-related MADS-box genes are involved in plant responses to various abiotic stresses and wounding (e.g., from insect feeding) (Castelán-Muñoz et al., 2019), which is consistent with carnivory origin through plant defense mechanisms (Pavlovic̀ and Mithöfer, 2019). Moreover, some studies indicate that MADS-box TFs may positively affect biosynthesis of anthocyanins (Jaakola et al., 2010; Zhao et al., 2019) used by traps to attract insects. The presence of highly conserved MADS-box genes associated with flowering and defense in all angiosperms may be the reason why a pitfall trap originated independently six times in diverse plant lineages.

Thus, non-carnivorous *Nepenthes* predecessor plants could use the MADS-box gene set specifying floral meristems and organs for adaptation to stressful conditions and nutrient deficiency. As a result, they develop a new structure, leaf-tendril-trap, based on the vegetative leaf, using the existing pathways for the formation of floral organs (tepal and pistil) (Figure 7). A recently shown WGD event in the last common ancestor of Droseraceae carnivorous plants (Palfalvi et al., 2020) led to the MADS-box genes duplication, and it can be speculated that leaves have acquired the ability to express such MADS-box paralogous genes, which, among other mechanisms, resulted in the leaf-tendril-trap development.

It has been shown that MADS-box genes involved in the initiation of flowering and floral organogenesis can also play an important role in the development of leaves (Burko et al., 2013; Gregis et al., 2013) and roots (Gan et al., 2005; Alvarez-Buylla et al., 2019). Therefore, the leaf-tendril-trap structure can be considered a specialized modified organ with its own unique regulatory pathway that evolved through co-option of genes from the networks controlling SAM identity and organization, leaf and root development, and flower morphogenesis.

The results obtained may clarify the genetic patterns of pitcher trap initiation and development and help answer the question of the origin of the plant carnivory syndrome. For example, given that a protocol for *Nepenthes mirabilis in vitro* regeneration and *Agrobacterium*-mediated transformation has been developed (Miguel et al., 2020), it is possible to obtain transgenic *Nepenthes* plants in which individual MADS-box genes are either overexpressed or silenced through various mechanisms such as CRISPR/Cas9-based genome editing. It would also be useful to compare the flower- and trap-specific paralogs of the MADS-box genes, including their regulatory regions. Evaluation of the morphology, genomics, and proteomics of traps from such transgenic plants could shed light on the trap-specific functions of the analyzed MADS-box genes and their roles in trap evolution. Also, modern methods for analysis of protein-protein interactions and transcription factor target genes should make possible to compare the functional activity of MADS-box genes in the trap with that of MADS-box genes known to be involved in flowering initiation and flower development.

## Data Availability Statement

The original contributions presented in the study are publicly available. This data can be found here: NCBI, accession number: PRJNA487526 (https://www.ncbi.nlm.nih.gov/bioproject/PRJNA487526).

## Author Contributions

NR and EK: conceptualization. MF, EK, and EG: plant material. NR, AB, and AM: methodology. AB: software. MF and MS: validation. AB and AS: formal analysis. NR: data curation. AS: Writing–original draft preparation. AS, EK, and NR: writing–review and editing. NR: supervision. All authors contributed to the article and approved the submitted version.

## Conflict of Interest

The authors declare that the research was conducted in the absence of any commercial or financial relationships that could be construed as a potential conflict of interest.

## Footnotes

1 https://github.com/najoshi/sickle

2 http://transdecoder.github.io

3 https://www.ncbi.nlm.nih.gov/

4 http://www.geneontology.org/

5 http://www.genome.jp/kegg/

6 http://planttfdb.cbi.pku.edu.cn/

7 http://mapman.gabipd.org/web/guest/mapcave

8 https://genemania.org/

9 http://trinotate.github.io

10 https://www.uniprot.org/

11 https://github.com/kevinblighe/EnhancedVolcano

12 http://bioinformatics.psb.ugent.be/webtools/Venn/

13 https://blast.ncbi.nlm.nih.gov/

14 https://www.graphpad.com/scientific-software/prism/

## Supplementary Material

Supplementary Figure 1 Gene Ontology (GO) BP terms most enriched in leaf **(a)** early pitcher, **(b)** DEGs (leaves vs. early pitcher), and in leaf **(c)**, and mature pitcher **(d)**, DEGs (leaves vs. mature pitcher). GO MF terms most enriched in leaf **(e)** early pitcher, **(f)** DEGs (leaves vs. early pitcher), in leaf **(g)**, and mature pitcher **(h)** DEGs (leaves vs. mature pitcher).

Supplementary Figure 2 *Nepenthes* transcriptomes in Kyoto Encyclopedia of Genes and Genomes (KEGG) PATHWAY **(a,c)**, and KEGG BRITE **(b)** terms.

Supplementary Figure 3 MapMan scheme of differentially regulated transcripts associated with secondary metabolism pathways: leaves vs. young pitcher **(a)**, mature pitcher vs. leaves **(b)**, mature pitcher vs. early pitcher **(c)**, and mature pitcher vs. young pitcher **(d)**.

Supplementary Figure 4 MapMan scheme of differentially regulated transcripts associated with photosynthetic pathways: mature pitcher vs. leaves **(a)**, mature pitcher vs. early pitcher **(b)**, and mature pitcher vs. young pitcher **(c)**.

Supplementary Figure 5 MapMan scheme of differentially regulated transcripts associated with stress response pathways: leaves vs. young pitcher **(a)**, mature pitcher vs. leaves **(b)**, mature pitcher vs. early pitcher **(c)**, and early pitcher vs. young pitcher **(d)**.

Supplementary Figure 6 Phylogenetic tree constructed based on the sequences of MADS-domain proteins from *Nepenthes* × *ventrata* (NveMADS1-45), *Arabidopsis thaliana*, and *Beta vulgaris* (Bve): AGAMOUS/AGL11 clade **(A)**; APETALA1/FRUITFULL and APETALA3/PISTILLATA **(B)**; SEPALLATA/AGL6 **(C)**; SVP/AGL24 **(D)**; and Type I **(E)**.

Supplementary Figure 7 Distance trees constructed for each NveMADS1-45 protein sequence using the Fast Minimum Evolution method (NCBI). Green corresponds to eudicots, yellow – to the query protein.

Supplementary Figure 8 Relative expression of genes related to flowering (*AGAMOUS*, *SEPALLATA 3*, and *MYB17*) and anthocyanin biosynthesis (*MYB113*, *bHLH001*, *CHS1*, *CHS2*, *CHI*, *F3H*, *F3’5’H*, *DFR*, *ANS*, and *UFGT*) determined by real-time quantitative PCR (RT-qPCR).

Supplementary Table 1 Statistics of *Nepenthes* × *ventrata* transcriptome assembly.

Supplementary Table 2 Assembled *Nepenthes* × *ventrata* transcripts with read numbers normalized among transcriptomes.

Supplementary Table 3 Species distribution of the top BLAST hits for assembled *Nepenthes* × *ventrata* transcripts.

Supplementary Table 4 Hierarchical GO annotation of the assembled *Nepenthes* × *ventrata* transcripts.

Supplementary Table 5 Annotation of the assembled *Nepenthes* × *ventrata* transcripts in GO terms.

Supplementary Table 6 Differentially expressed transcripts (upregulation ≥ 2 times) in the assembled *Nepenthes* × *ventrata* transcriptomes.

Supplementary Table 7 Gene Ontology (GO) enrichment and depletion in the assembled *Nepenthes* × *ventrata* transcriptomes.

Supplementary Table 8 Kyoto Encyclopedia of Genes and Genomes (KEGG) annotation of the assembled *Nepenthes* × *ventrata* transcripts.

Supplementary Table 9 Kyoto Encyclopedia of Genes and Genomes enrichment in the assembled *Nepenthes* × *ventrata* transcriptomes.

Supplementary Table 10 Plant Transcription Factor Database (PlantTFDB) annotation of the assembled *Nepenthes* × *ventrata* transcripts.

Supplementary Table 11 Differentially expressed unique genes (upregulation ≥ 4 times) in the assembled *Nepenthes* × *ventrata* transcriptomes.

Supplementary Table 12 MADS-box related transcripts in the *Nepenthes* × *ventrata* transcriptomes.

Supplementary Table 13 MADS-related transcripts in the assembled transcriptomes of the *Nepenthes* × *ventrata* mature pitcher and *Nepenthes khasiana* inflorescence.

Supplementary Table 14 GeneMANIA predicted functions and interactions of putative *Nepenthes* × *ventrata* type I MADS-domain proteins.

Supplementary Table 15 GeneMANIA predicted functions and interactions of putative *Nepenthes* × *ventrata* type II MADS-domain proteins.

Supplementary Table 16 Real-time quantitative PCR (RT-qPCR) primers for *Nepenthes* × *ventrata* unigene expression analysis.
