# Supplementary material for: Nepenthes × ventrata Transcriptome Profiling Reveals a Similarity Between the Evolutionary Origins of Carnivorous Traps and Floral Organs
Source: Front Plant Sci. 2021 May 28;12:643137. doi: 10.3389/fpls.2021.643137 (PMC8194089; doi:10.3389/fpls.2021.643137)
Supplement: Supplementary Figure 1 — Gene Ontology (GO) BP terms most enriched in leaf (a) early pitcher, (b) DEGs (leaves vs. early pitcher), and in leaf (c), and mature pitcher (d), DEGs (leaves vs. mature pitcher). GO MF terms most enriched in leaf (e) early pitcher, (f) DEGs (leaves vs. early pitcher), in leaf (g), and mature pitcher (h) DEGs (leaves vs. mature pitcher). [file Data_Sheet_1.PDF]

Nepenthes × ventrata transcriptome profiling reveals a similarity between the evolutionary origins of carnivorous traps and flowers

Anna V. Shchennikova*, Alexey V. Beletsky, Mikhail A. Filyushin, Maria A. Slugina, Eugeny V. Gruzdev, Andrey V. Mardanov, Elena Z. Kochieva, Nikolai V. Ravin*

Institute of Bioengineering, Research Center of Biotechnology of the Russian Academy of Sciences, Moscow 119071, Russia

*Correspondence and requests for materials should be addressed to A.S. (email: shchennikova@yandex.ru) or N.R. (email: nravin@biengi.ac.ru)

Supplementary Figures:

S1 – GO BP terms most enriched in leaf (a) and early pitcher (b) DEGs (leaves vs. early pitcher), and in leaf (c) and mature pitcher (d) DEGs (leaves vs. mature pitcher). GO MF terms most enriched in leaf (e) and early pitcher (f) DEGs (leaves vs. early pitcher), and in leaf (g) and mature pitcher (h) DEGs (leaves vs. mature pitcher).

The first principal component (axis X) corresponds to the number of hits (%; number of DETs in this category/number of transcripts in this category). The second component (Y axis) characterizes the differences in DET implication into different biological processes. Point diameter corresponds to the number of DETs, point colour – to the level of statistical significance.

S2 - *Nepenthes* transcriptomes in KEGG PATHWAY (a, c), and KEGG BRITE (b) terms.

S3 – MapMan scheme of differentially regulated transcripts associated with secondary metabolism pathways: leaves vs. young pitcher (a), mature pitcher vs. leaves (b), mature pitcher vs. early pitcher (c), and mature pitcher vs. young pitcher (d).

S4 – MapMan scheme of differentially regulated transcripts associated with photosynthetic pathways: mature pitcher vs. leaves (a), mature pitcher vs. early pitcher (b), and mature pitcher vs. young pitcher (c).

S5 – MapMan scheme of differentially regulated transcripts associated with stress response pathways: leaves vs. young pitcher (a), mature pitcher vs. leaves (b), mature pitcher vs. early pitcher (c), and early pitcher vs. young pitcher (d).

S6 – Phylogenetic tree constructed based on the sequences of MADS-domain proteins from *Nepenthes* × *ventrata* (NveMADS1-45), *Arabidopsis thaliana*, and *Beta vulgaris* (Bve): AGAMOUS/AGL11 clade (A); APETALA1/FRUITFULL and APETALA3/PISTILLATA (B); SEPALLATA/AGL6 (C); SVP/AGL24 (D); Type I (E). Analysis was performed using the Maximum Likelihood method based on the JTT matrix-based model in MEGA7.0.

S7 – Distance trees constructed for each NveMADS1-45 protein sequence of using the Fast Minimum Evolution method (NCBI). Green corresponds to eudicots, yellow – to query protein.

S8 – Relative expression of genes related to flowering (*AGAMOUS*, *SEPALLATA 3*, and *MYB17*) and anthocyanin biosynthesis (*MYB113*, *bHLH001*, *CHS1*, *CHS2*, *CHI*, *F3H*, *F3'5'H*, *DFR*, *ANS*, and *UFGT*) determined by RT-qPCR

Figure S1

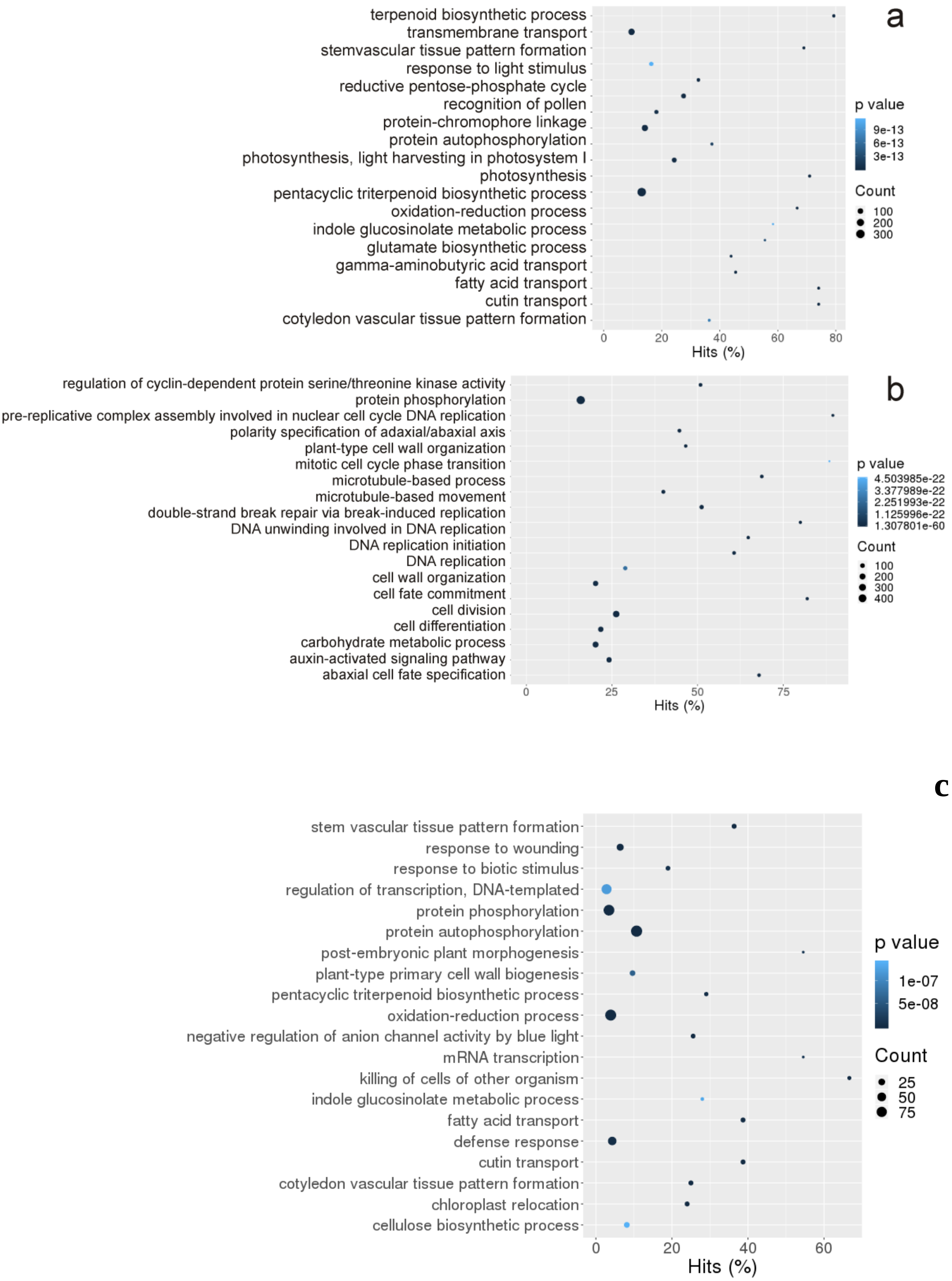
a
terpenoid biosynthetic process
transmembrane transport
stemvascular tissue pattern formation
response to light stimulus
reductive pentose-phosphate cycle
recognition of pollen
protein-chromophore linkage
protein autophosphorylation
photosynthesis, light harvesting in photosystem I
photosynthesis
pentacyclic triterpenoid biosynthetic process
oxidation-reduction process
indole glucosinolate metabolic process
glutamate biosynthetic process
gamma-aminobutyric acid transport
fatty acid transport
cutin transport
cotyledon vascular tissue pattern formation
0
20
40
60
80
Hits (%)
p value
9e-13
6e-13
3e-13
Count
100
200
300
b
regulation of cyclin-dependent protein serine/threonine kinase activity
protein phosphorylation
pre-replicative complex assembly involved in nuclear cell cycle DNA replication
polarity specification of adaxial/abaxial axis
plant-type cell wall organization
mitotic cell cycle phase transition
microtubule-based process
microtubule-based movement
double-strand break repair via break-induced replication
DNA unwinding involved in DNA replication
DNA replication initiation
DNA replication
cell wall organization
cell fate commitment
cell division
cell differentiation
carbohydrate metabolic process
auxin-activated signaling pathway
abaxial cell fate specification
0
25
50
75
Hits (%)
p value
4.503985e-22
3.377989e-22
2.251993e-22
1.125996e-22
1.307801e-60
Count
100
200
300
400
c
stem vascular tissue pattern formation
response to wounding
response to biotic stimulus
regulation of transcription, DNA-templated
protein phosphorylation
protein autophosphorylation
post-embryonic plant morphogenesis
plant-type primary cell wall biogenesis
pentacyclic triterpenoid biosynthetic process
oxidation-reduction process
negative regulation of anion channel activity by blue light
mRNA transcription
killing of cells of other organism
indole glucosinolate metabolic process
fatty acid transport
defense response
cutin transport
cotyledon vascular tissue pattern formation
chloroplast relocation
cellulose biosynthetic process
0
20
40
60
Hits (%)
p value
1e-07
5e-08
Count
25
50
75

d

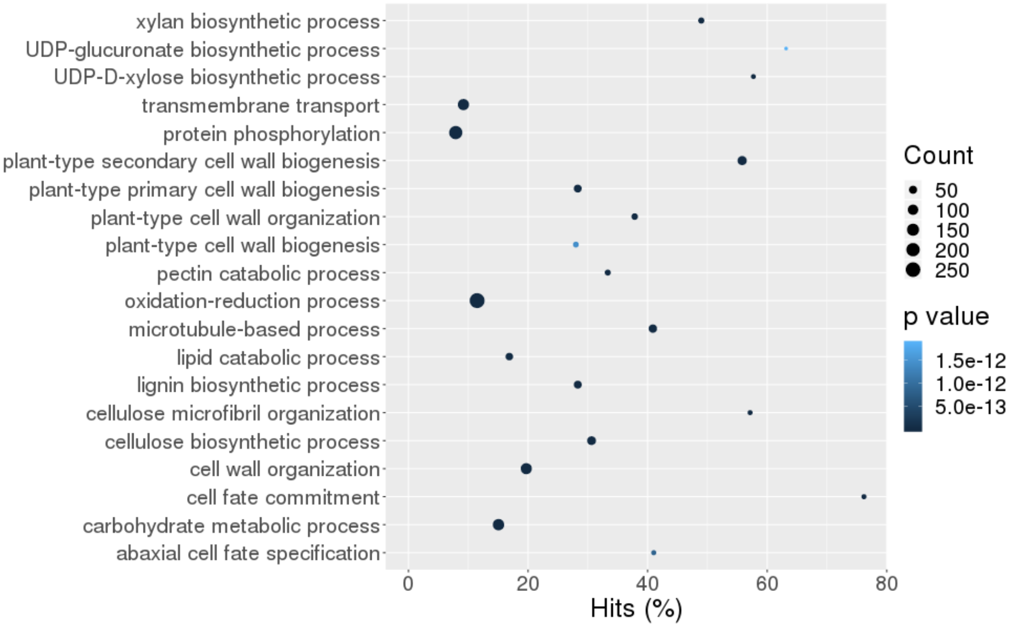
xylan biosynthetic process
UDP-glucuronate biosynthetic process
UDP-D-xylose biosynthetic process
transmembrane transport
protein phosphorylation
plant-type secondary cell wall biogenesis
plant-type primary cell wall biogenesis
plant-type cell wall organization
plant-type cell wall biogenesis
pectin catabolic process
oxidation-reduction process
microtubule-based process
lipid catabolic process
lignin biosynthetic process
cellulose microfibril organization
cellulose biosynthetic process
cell wall organization
cell fate commitment
carbohydrate metabolic process
abaxial cell fate specification
0
20
40
60
80
Hits (%)
Count
50
100
150
200
250
p value
1.5e-12
1.0e-12
5.0e-13

e

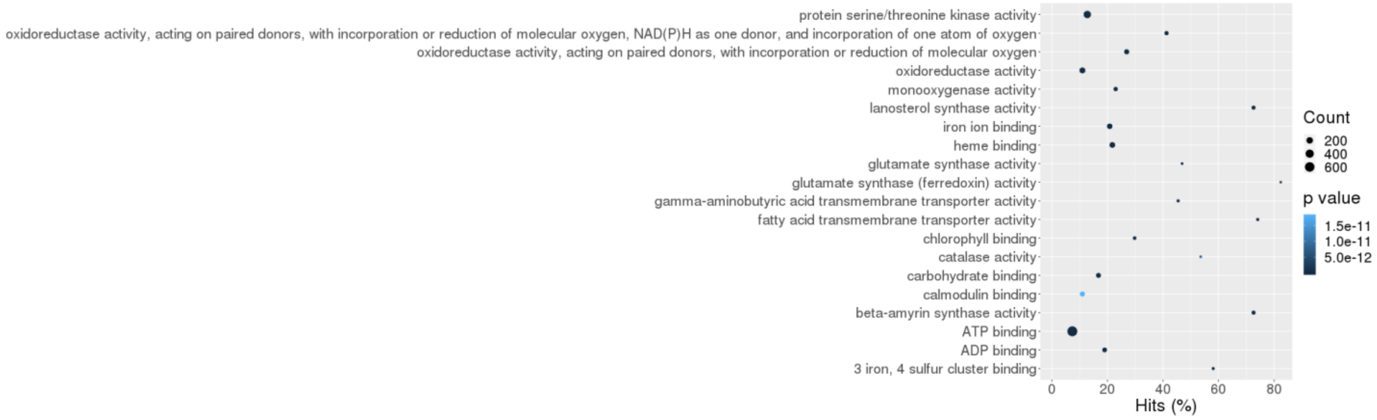
protein serine/threonine kinase activity
oxidoreductase activity, acting on paired donors, with incorporation or reduction of molecular oxygen, NAD(P)H as one donor, and incorporation of one atom of oxygen
oxidoreductase activity, acting on paired donors, with incorporation or reduction of molecular oxygen
oxidoreductase activity
monooxygenase activity
lanosterol synthase activity
iron ion binding
heme binding
glutamate synthase activity
glutamate synthase (ferredoxin) activity
gamma-aminobutyric acid transmembrane transporter activity
fatty acid transmembrane transporter activity
chlorophyll binding
catalase activity
carbohydrate binding
calmodulin binding
beta-amyrin synthase activity
ATP binding
ADP binding
3 iron, 4 sulfur cluster binding
0
20
40
60
80
Hits (%)
Count
200
400
600
p value
1.5e-11
1.0e-11
5.0e-12

f

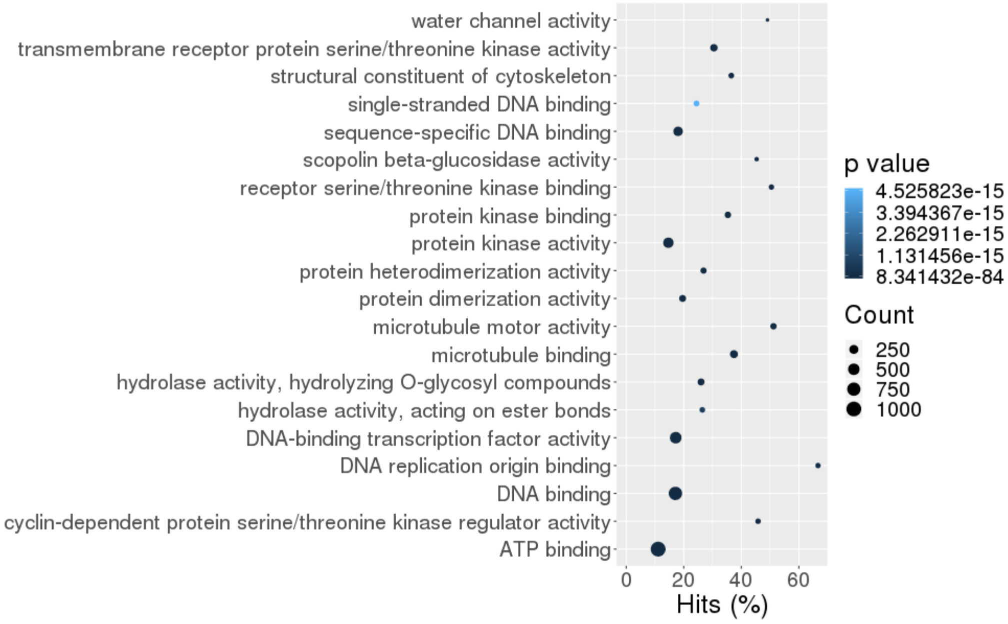
water channel activity
transmembrane receptor protein serine/threonine kinase activity
structural constituent of cytoskeleton
single-stranded DNA binding
sequence-specific DNA binding
scopolin beta-glucosidase activity
receptor serine/threonine kinase binding
protein kinase binding
protein kinase activity
protein heterodimerization activity
protein dimerization activity
microtubule motor activity
microtubule binding
hydrolase activity, hydrolyzing O-glycosyl compounds
hydrolase activity, acting on ester bonds
DNA-binding transcription factor activity
DNA replication origin binding
DNA binding
cyclin-dependent protein serine/threonine kinase regulator activity
ATP binding
0
20
40
60
Hits (%)
p value
4.525823e-15
3.394367e-15
2.262911e-15
1.131456e-15
8.341432e-84
Count
250
500
750
1000

g

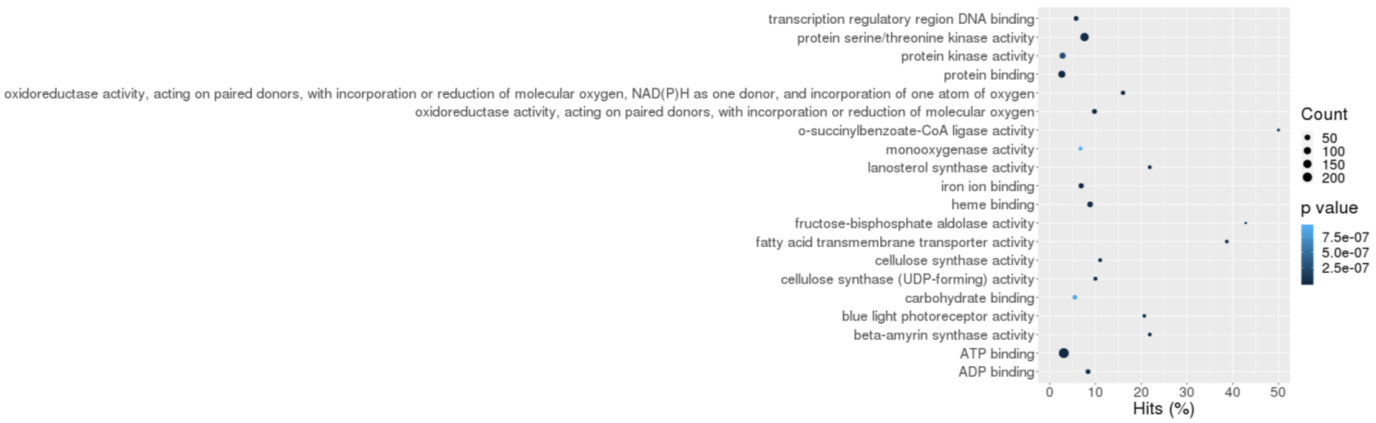
transcription regulatory region DNA binding
protein serine/threonine kinase activity
protein kinase activity
protein binding
oxidoreductase activity, acting on paired donors, with incorporation or reduction of molecular oxygen, NAD(P)H as one donor, and incorporation of one atom of oxygen
oxidoreductase activity, acting on paired donors, with incorporation or reduction of molecular oxygen
o-succinylbenzoate-CoA ligase activity
monooxygenase activity
lanosterol synthase activity
iron ion binding
heme binding
fructose-bisphosphate aldolase activity
fatty acid transmembrane transporter activity
cellulose synthase activity
cellulose synthase (UDP-forming) activity
carbohydrate binding
blue light photoreceptor activity
beta-amyrin synthase activity
ATP binding
ADP binding
0
10
20
30
40
50
Hits (%)
Count
50
100
150
200
p value
7.5e-07
5.0e-07
2.5e-07

h

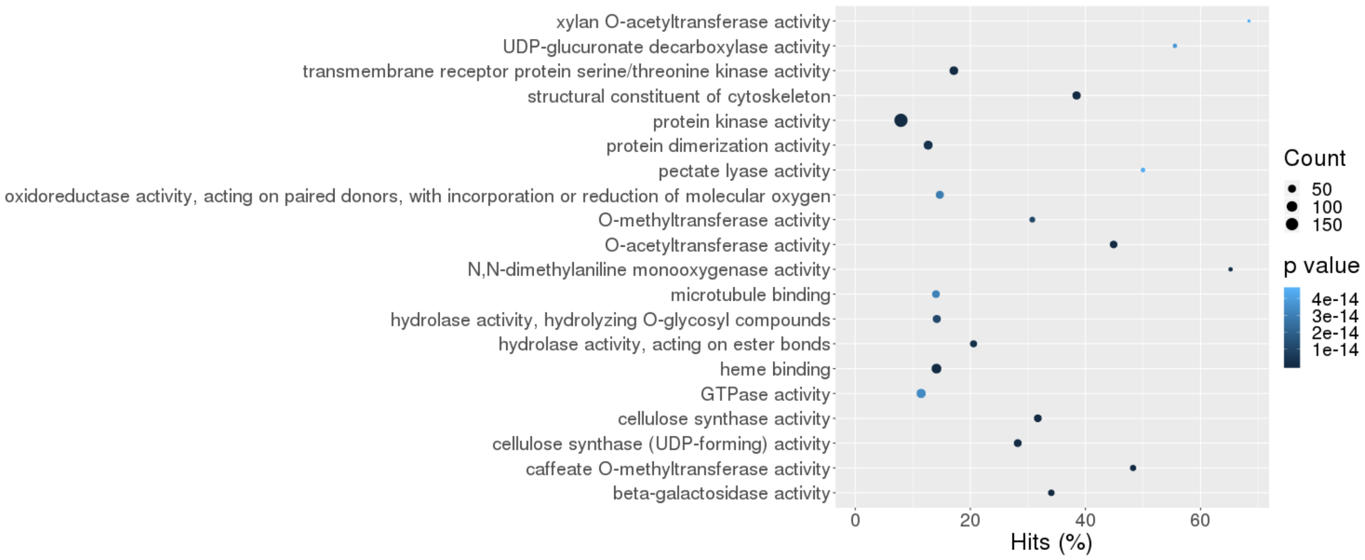
xylan O-acetyltransferase activity
UDP-glucuronate decarboxylase activity
transmembrane receptor protein serine/threonine kinase activity
structural constituent of cytoskeleton
protein kinase activity
protein dimerization activity
pectate lyase activity
oxidoreductase activity, acting on paired donors, with incorporation or reduction of molecular oxygen
O-methyltransferase activity
O-acetyltransferase activity
N,N-dimethylaniline monooxygenase activity
microtubule binding
hydrolase activity, hydrolyzing O-glycosyl compounds
hydrolase activity, acting on ester bonds
heme binding
GTPase activity
cellulose synthase activity
cellulose synthase (UDP-forming) activity
caffeate O-methyltransferase activity
beta-galactosidase activity
0
20
40
60
Hits (%)
Count
50
100
150
p value
4e-14
3e-14
2e-14
1e-14

Figure S2

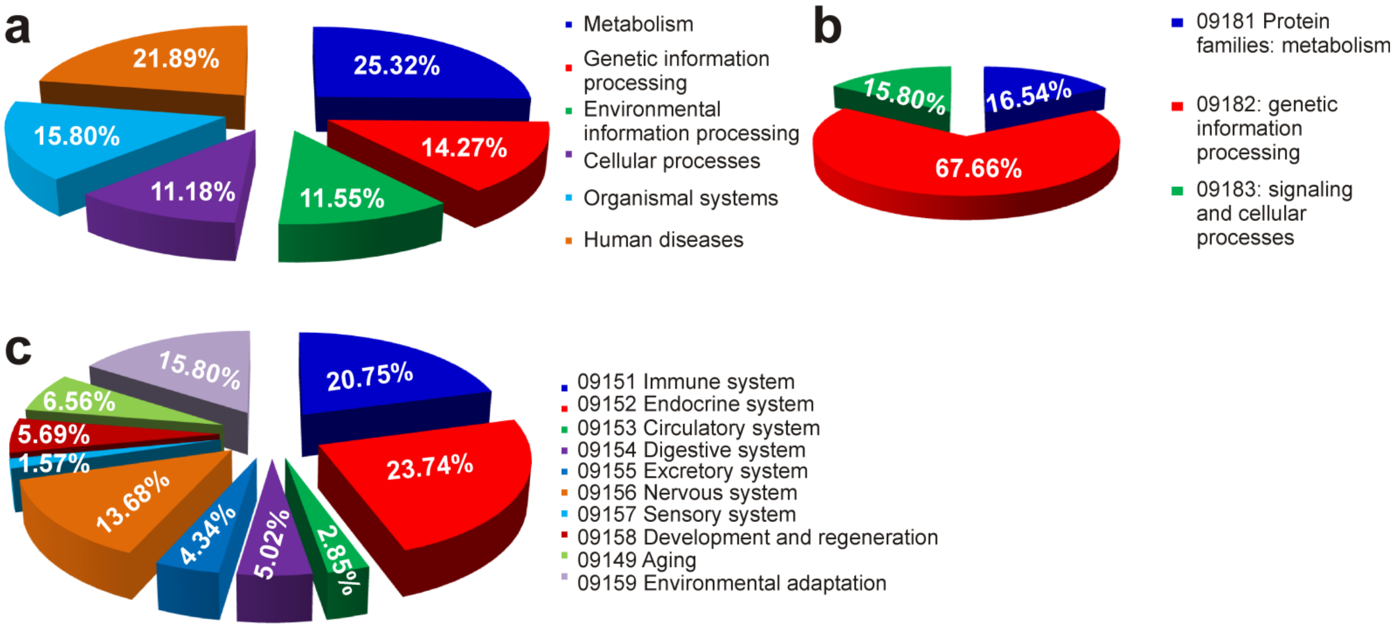
a
21.89%
25.32%
15.80%
14.27%
11.18%
11.55%
Metabolism
Genetic information processing
Environmental information processing
Cellular processes
Organismal systems
Human diseases
b
15.80%
16.54%
67.66%
09181 Protein families: metabolism
09182: genetic information processing
09183: signaling and cellular processes
c
15.80%
20.75%
6.56%
5.69%
1.57%
23.74%
13.68%
4.34%
5.02%
2.85%
09151 Immune system
09152 Endocrine system
09153 Circulatory system
09154 Digestive system
09155 Excretory system
09156 Nervous system
09157 Sensory system
09158 Development and regeneration
09149 Aging
09159 Environmental adaptation

Figure S3

a

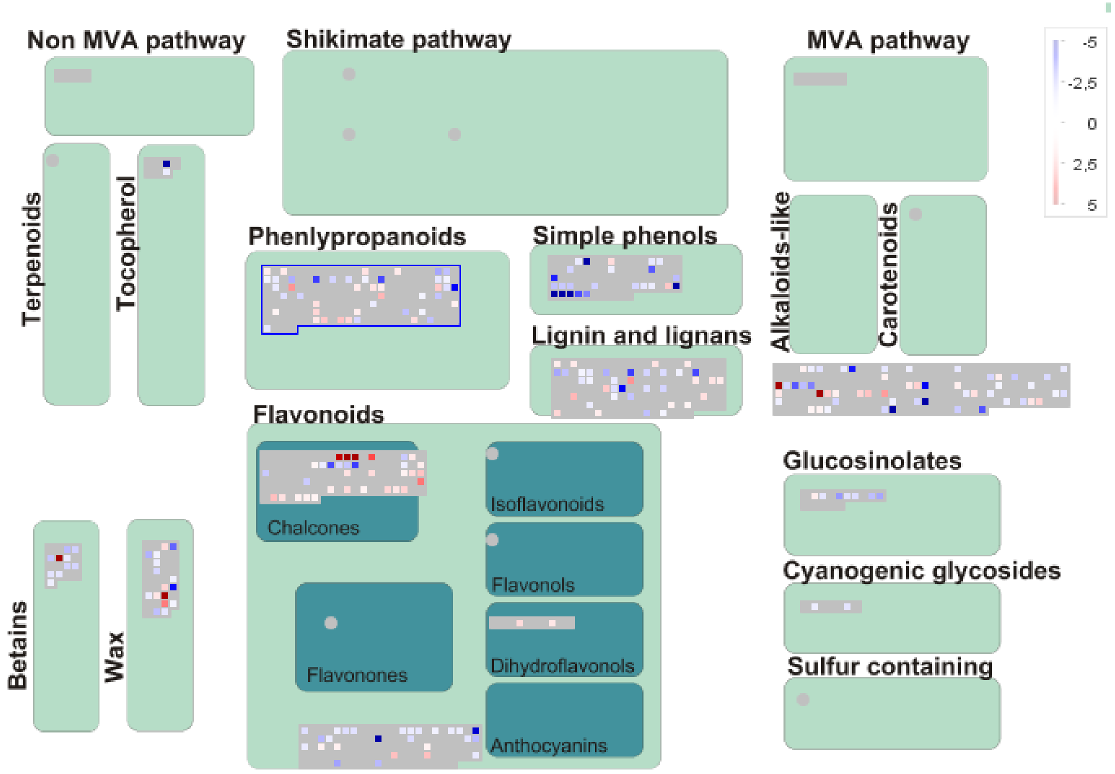

Non MVA pathway
Shikimate pathway
MVA pathway
-5
-2,5
0
2,5
5
Terpenoids
Tocopherol
Phenlypropanoids
Simple phenols
Lignin and lignans
Alkaloids-like
Carotenoids
Flavonoids
Chalcones
Isoflavonoids
Flavonols
Flavonones
Dihydroflavonols
Anthocyanins
Glucosinolates
Cyanogenic glycosides
Sulfur containing
Betains
Wax

b

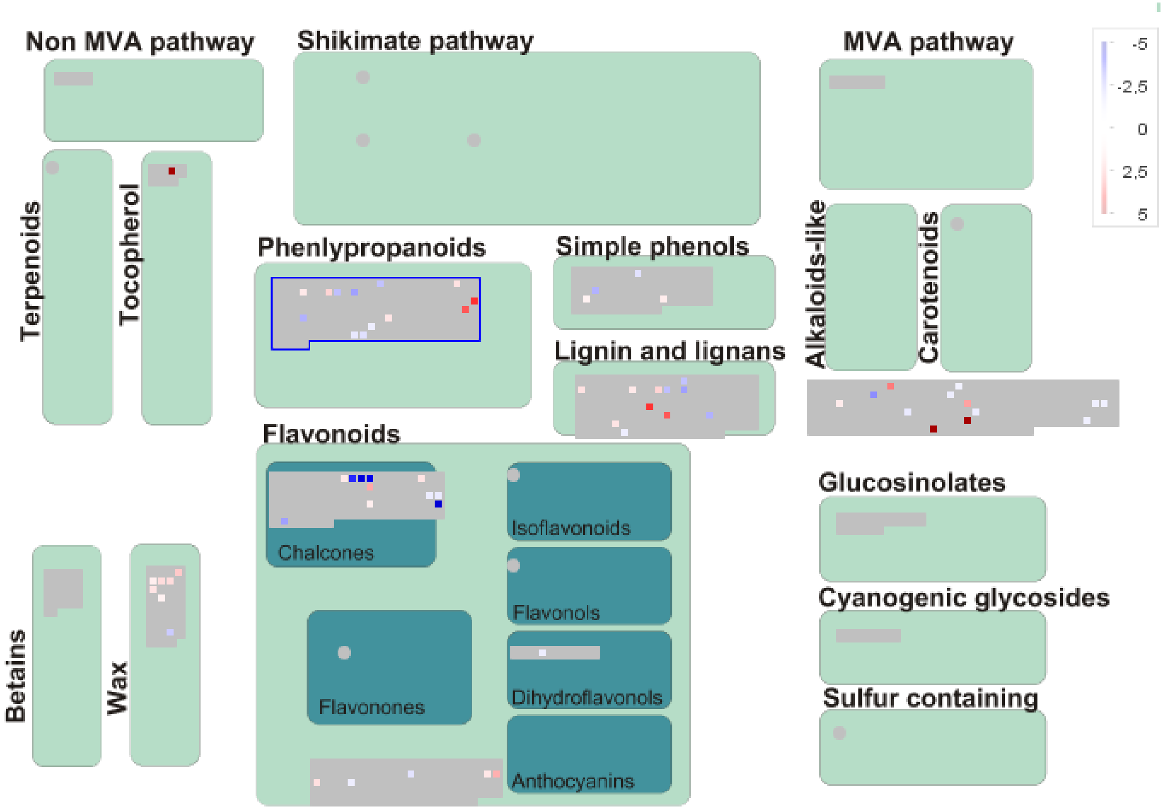

Non MVA pathway
Shikimate pathway
MVA pathway
-5
-2,5
0
2,5
5
Terpenoids
Tocopherol
Phenlypropanoids
Simple phenols
Lignin and lignans
Alkaloids-like
Carotenoids
Flavonoids
Chalcones
Isoflavonoids
Flavonols
Flavonones
Dihydroflavonols
Anthocyanins
Glucosinolates
Cyanogenic glycosides
Sulfur containing
Betains
Wax

c

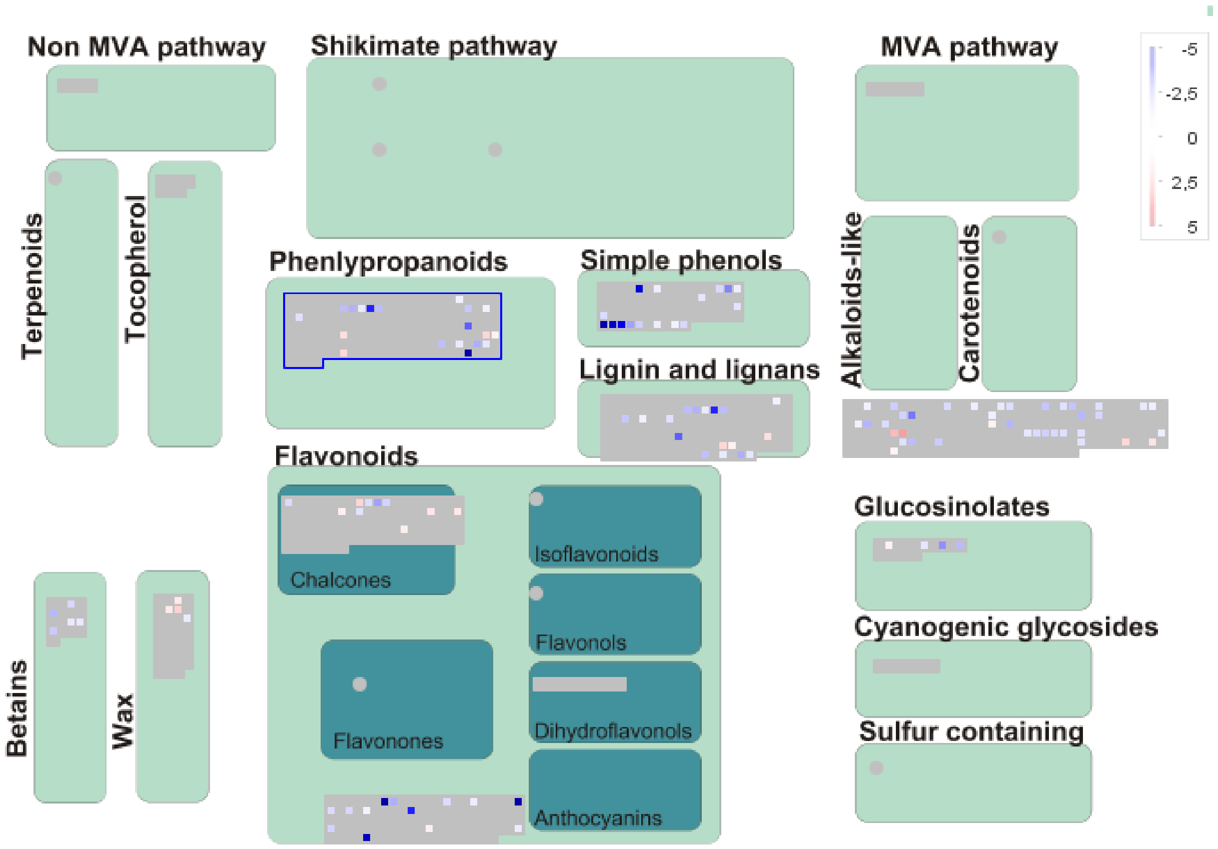
Non MVA pathway
Shikimate pathway
MVA pathway
-5
-2,5
0
2,5
5
Terpenoids
Tocopherol
Phenlypropanoids
Simple phenols
Lignin and lignans
Alkaloids-like
Carotenoids
Flavonoids
Chalcones
Isoflavonoids
Flavonols
Flavonones
Dihydroflavonols
Anthocyanins
Glucosinolates
Cyanogenic glycosides
Sulfur containing
Betains
Wax

d

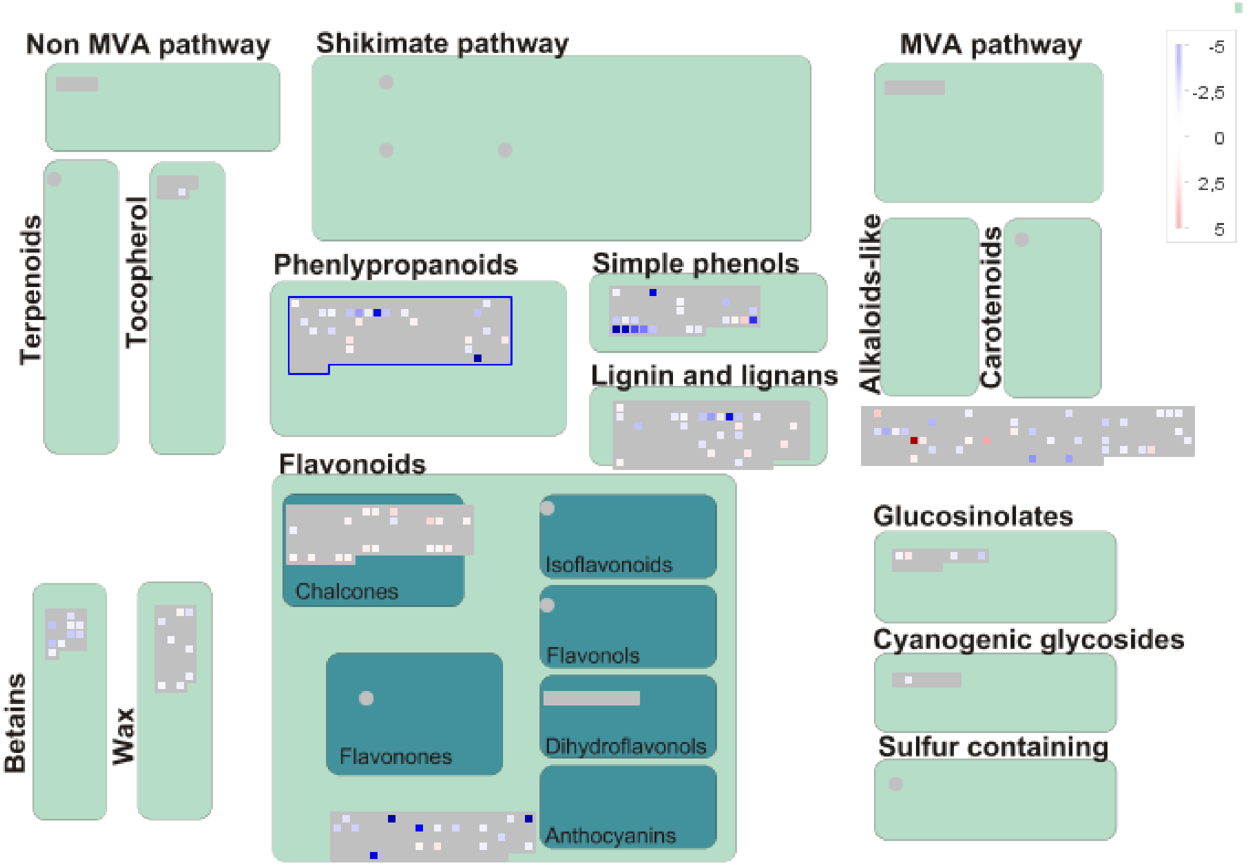
Non MVA pathway
Shikimate pathway
MVA pathway
-5
-2,5
0
2,5
5
Terpenoids
Tocopherol
Phenlypropanoids
Simple phenols
Lignin and lignans
Alkaloids-like
Carotenoids
Flavonoids
Chalcones
Isoflavonoids
Flavonols
Flavonones
Dihydroflavonols
Anthocyanins
Glucosinolates
Cyanogenic glycosides
Sulfur containing
Betains
Wax

Figure S4

a

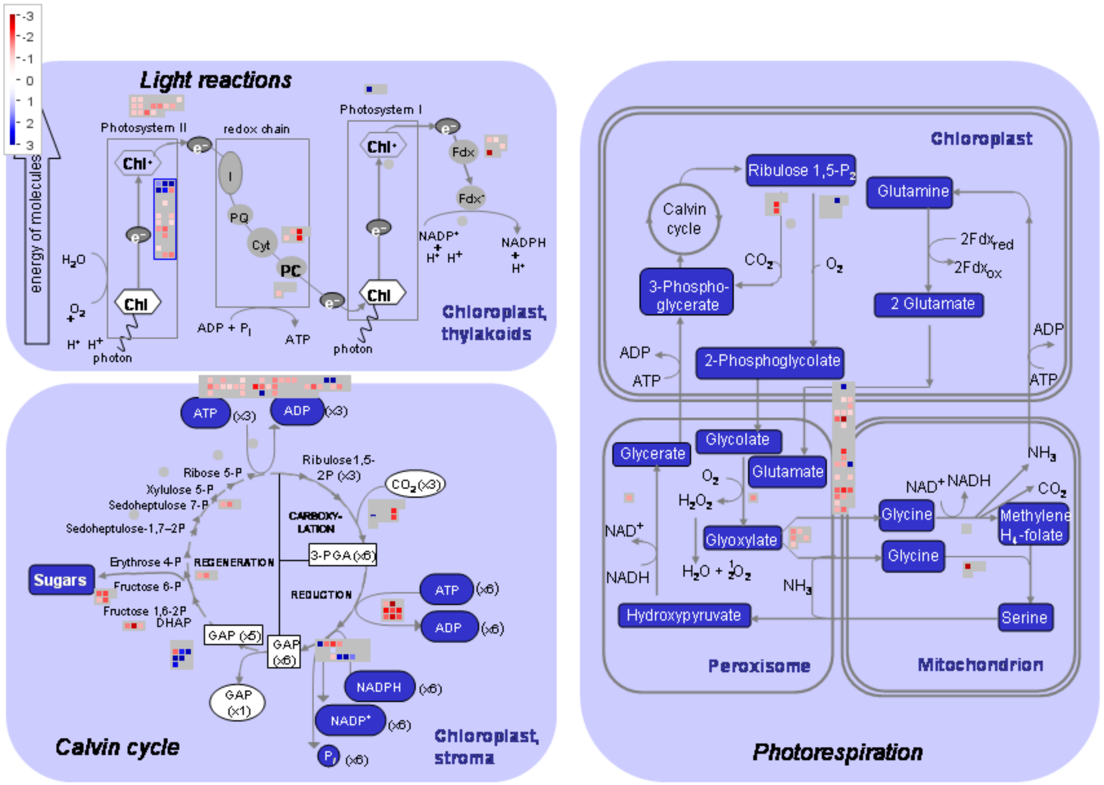

-3
-2
-1
0
1
2
3
energy of molecules
Light reactions
Photosystem II
redox chain
Photosystem I
Chl⁺
Chl
e⁻
PQ
Cyt
PC
Fdx
Fdx⁻
H_2O
O_2
$NADP^+$
NADPH
$ADP + P_i$
ATP
photon
Chloroplast, thylakoids
ATP (x3)
ADP (x3)
Ribose 5-P
Xylulose 5-P
Sedoheptulose 7-P
Sedoheptulose-1,7-2P
Erythrose 4-P
Fructose 6-P
Fructose 1,6-2P
DHAP
Sugars
Ribulose1,5-2P (x3)
CO_2 (x3)
CARBOXY-LATION
3-PGA (x6)
REGENERATION
REDUCTION
ATP (x6)
ADP (x6)
GAP (x5)
GAP (x6)
GAP (x1)
NADPH (x6)
$NADP^+$ (x6)
P_i (x6)
Calvin cycle
Chloroplast, stroma
Chloroplast
Ribulose 1,5-P_2
Glutamine
Calvin cycle
CO_2
O_2
2Fdx$_{red}$
2Fdx$_{ox}$
3-Phospho-glycerate
2 Glutamate
ADP
ATP
2-Phosphoglycolate
Glycerate
Glycolate
Glutamate
H_2O_2
NAD^+
NADH
Glyoxylate
$H_2O + \frac{1}{2}O_2$
NH_3
Glycine
Methylene H_4-folate
Serine
Hydroxypyruvate
Peroxisome
Mitochondrion
Photorespiration

b

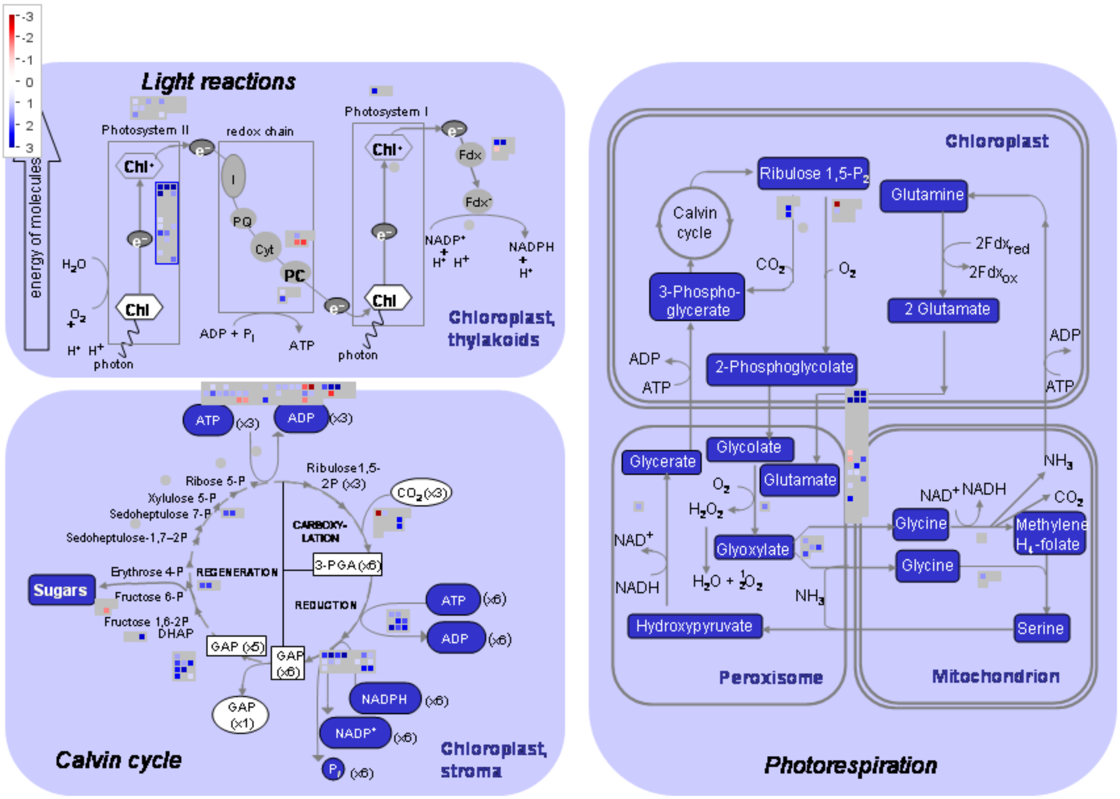

-3
-2
-1
0
1
2
3
energy of molecules
Light reactions
Photosystem II
redox chain
Photosystem I
Chl⁺
Chl
e⁻
PQ
Cyt
PC
Fdx
Fdx⁻
H_2O
O_2
$NADP^+$
NADPH
$ADP + P_i$
ATP
photon
Chloroplast, thylakoids
ATP (x3)
ADP (x3)
Ribose 5-P
Xylulose 5-P
Sedoheptulose 7-P
Sedoheptulose-1,7-2P
Erythrose 4-P
Fructose 6-P
Fructose 1,6-2P
DHAP
Sugars
Ribulose1,5-2P (x3)
CO_2 (x3)
CARBOXY-LATION
3-PGA (x6)
REGENERATION
REDUCTION
ATP (x6)
ADP (x6)
GAP (x5)
GAP (x6)
GAP (x1)
NADPH (x6)
$NADP^+$ (x6)
P_i (x6)
Calvin cycle
Chloroplast, stroma
Chloroplast
Ribulose 1,5-P_2
Glutamine
Calvin cycle
CO_2
O_2
2Fdx$_{red}$
2Fdx$_{ox}$
3-Phospho-glycerate
2 Glutamate
ADP
ATP
2-Phosphoglycolate
Glycerate
Glycolate
Glutamate
H_2O_2
NAD^+
NADH
Glyoxylate
$H_2O + \frac{1}{2}O_2$
NH_3
Glycine
Methylene H_4-folate
Serine
Hydroxypyruvate
Peroxisome
Mitochondrion
Photorespiration

c

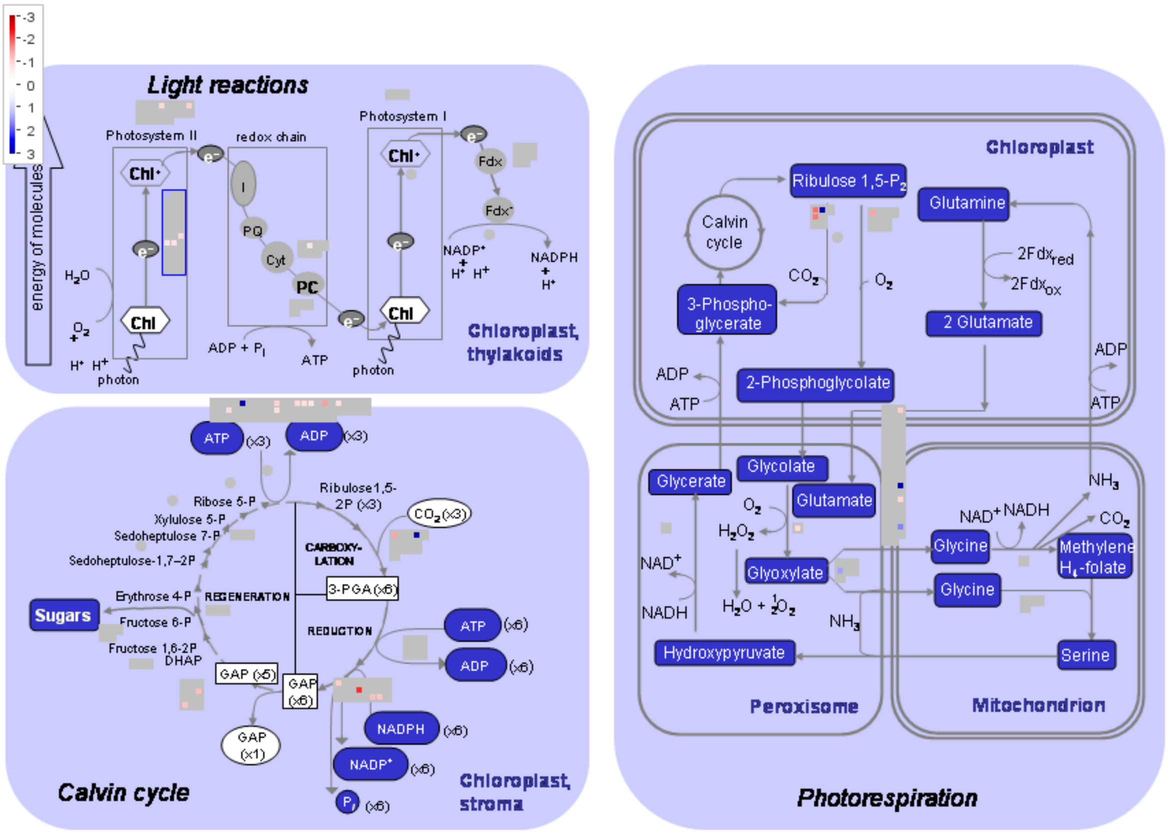
-3
-2
-1
0
1
2
3
energy of molecules
Light reactions
Photosystem II
redox chain
Photosystem I
Chl*
Chl
e⁻
I
PQ
Cyt
PC
Fdx
NADP⁺
NADPH
ADP + Pi
ATP
photon
Chloroplast, thylakoids
ATP
ADP
(x3)
Ribose 5-P
Xylulose 5-P
Sedoheptulose 7-P
Sedoheptulose-1,7-2P
Ribulose1,5-2P (x3)
CO2 (x3)
CARBOXY-LATION
REGENERATION
3-PGA (x6)
Erythrose 4-P
Fructose 6-P
Fructose 1,6-2P
DHAP
Sugars
REDUCTION
GAP (x5)
GAP (x6)
GAP (x1)
ATP (x6)
ADP (x6)
NADPH (x6)
NADP⁺ (x6)
Pi (x6)
Calvin cycle
Chloroplast, stroma
Chloroplast
Calvin cycle
Ribulose 1,5-P2
Glutamine
CO2
O2
2Fdxred
2Fdxox
3-Phospho-glycerate
2 Glutamate
ADP
ATP
2-Phosphoglycolate
Glycolate
Glycerate
Glutamate
O2
H2O2
NAD⁺
NADH
Glyoxylate
H2O + ½O2
NH3
Glycine
Methylene H4-folate
Glycine
Serine
Hydroxypyruvate
NAD⁺ NADH
CO2
Peroxisome
Mitochondrion
Photorespiration

Figure S5

a

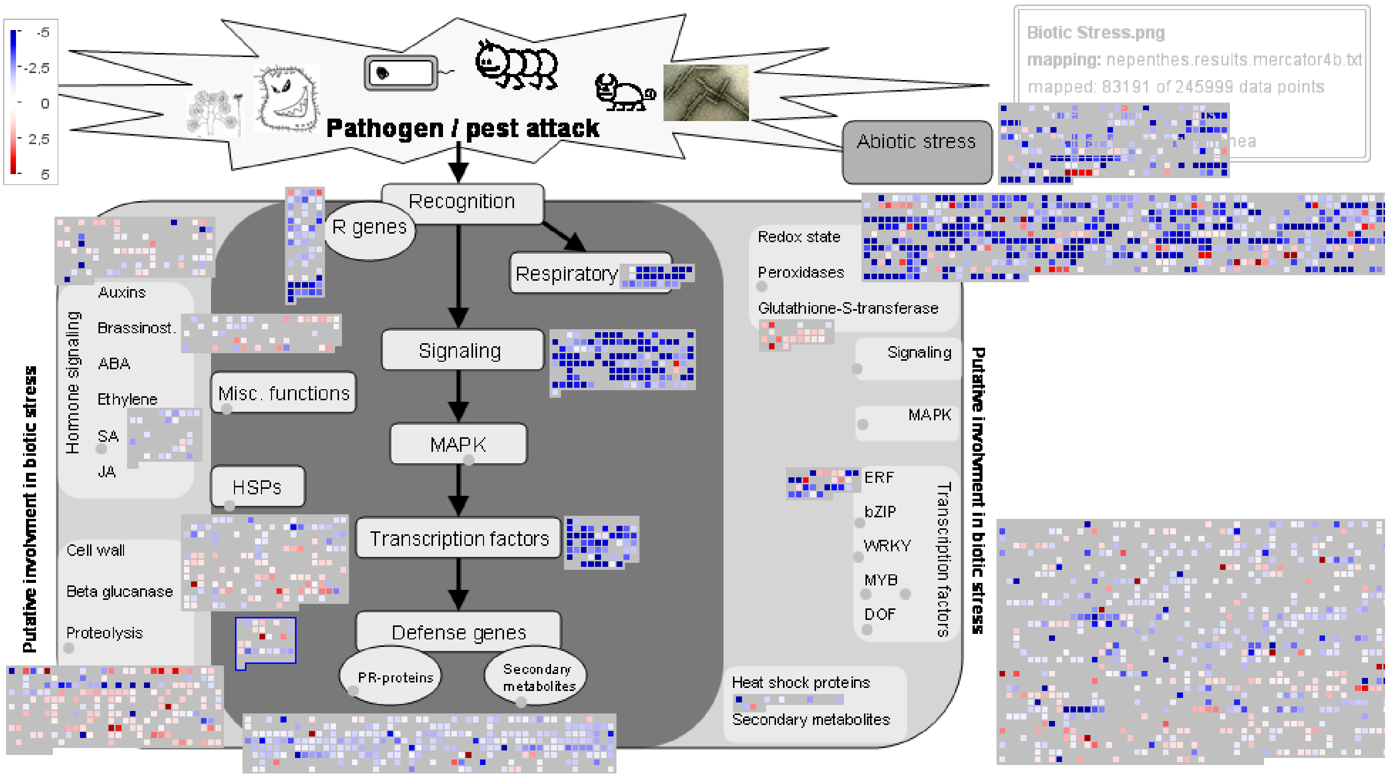
-5
-2,5
0
2,5
5
Pathogen / pest attack
Biotic Stress.png
mapping: nepenthes.results.mercator4b.txt
mapped: 83191 of 245999 data points
Abiotic stress
Recognition
R genes
Respiratory
Signaling
MAPK
Transcription factors
Defense genes
PR-proteins
Secondary metabolites
Misc. functions
HSPs
Hormone signaling
Auxins
Brassinost.
ABA
Ethylene
SA
JA
Cell wall
Beta glucanase
Proteolysis
Putative involvment in biotic stress
Redox state
Peroxidases
Glutathione-S-transferase
Signaling
MAPK
ERF
bZIP
WRKY
MYB
DOF
Transcription factors
Heat shock proteins
Secondary metabolites
Putative involvment in biotic stress

b

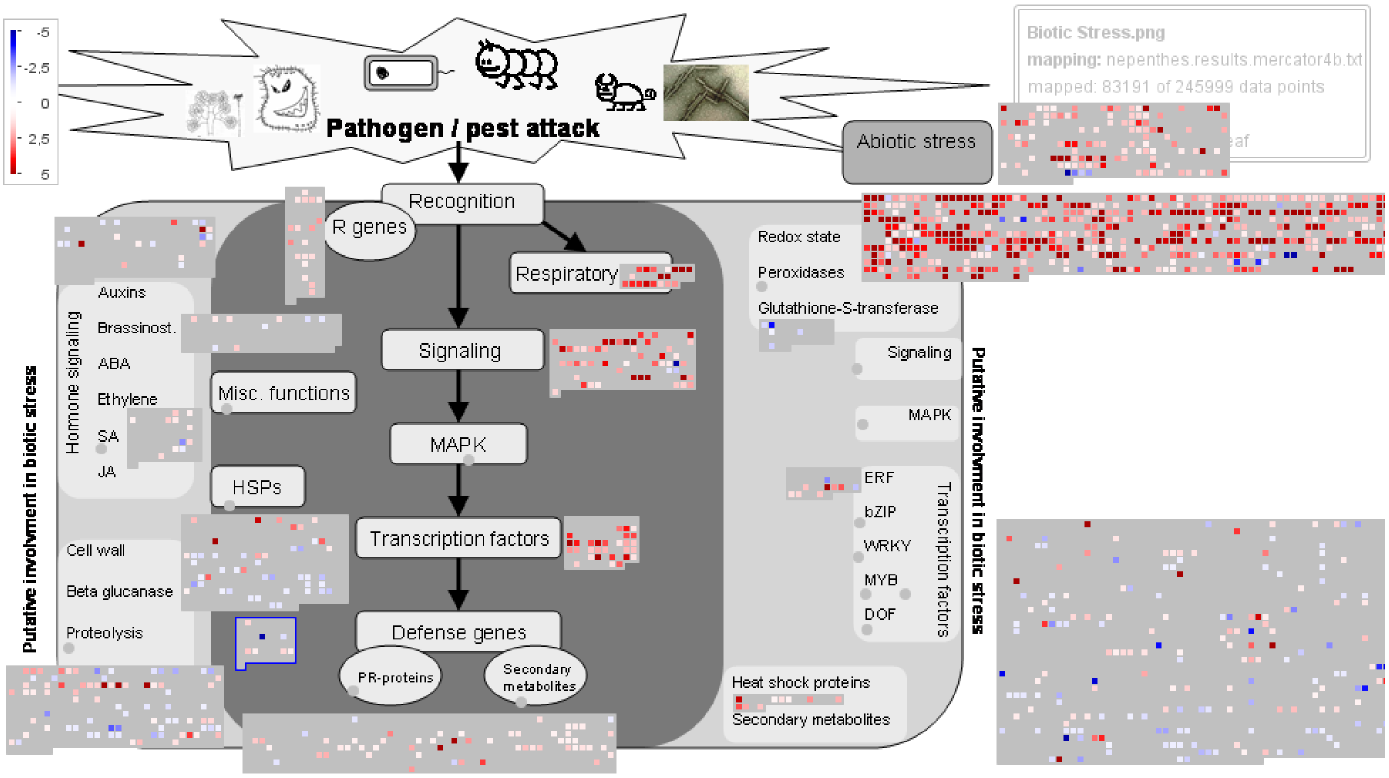
-5
-2,5
0
2,5
5
Pathogen / pest attack
Biotic Stress.png
mapping: nepenthes.results.mercator4b.txt
mapped: 83191 of 245999 data points
Abiotic stress
Recognition
R genes
Respiratory
Signaling
MAPK
Transcription factors
Defense genes
PR-proteins
Secondary metabolites
Misc. functions
HSPs
Hormone signaling
Auxins
Brassinost.
ABA
Ethylene
SA
JA
Cell wall
Beta glucanase
Proteolysis
Putative involvment in biotic stress
Redox state
Peroxidases
Glutathione-S-transferase
Signaling
MAPK
ERF
bZIP
WRKY
MYB
DOF
Transcription factors
Heat shock proteins
Secondary metabolites
Putative involvment in biotic stress

c

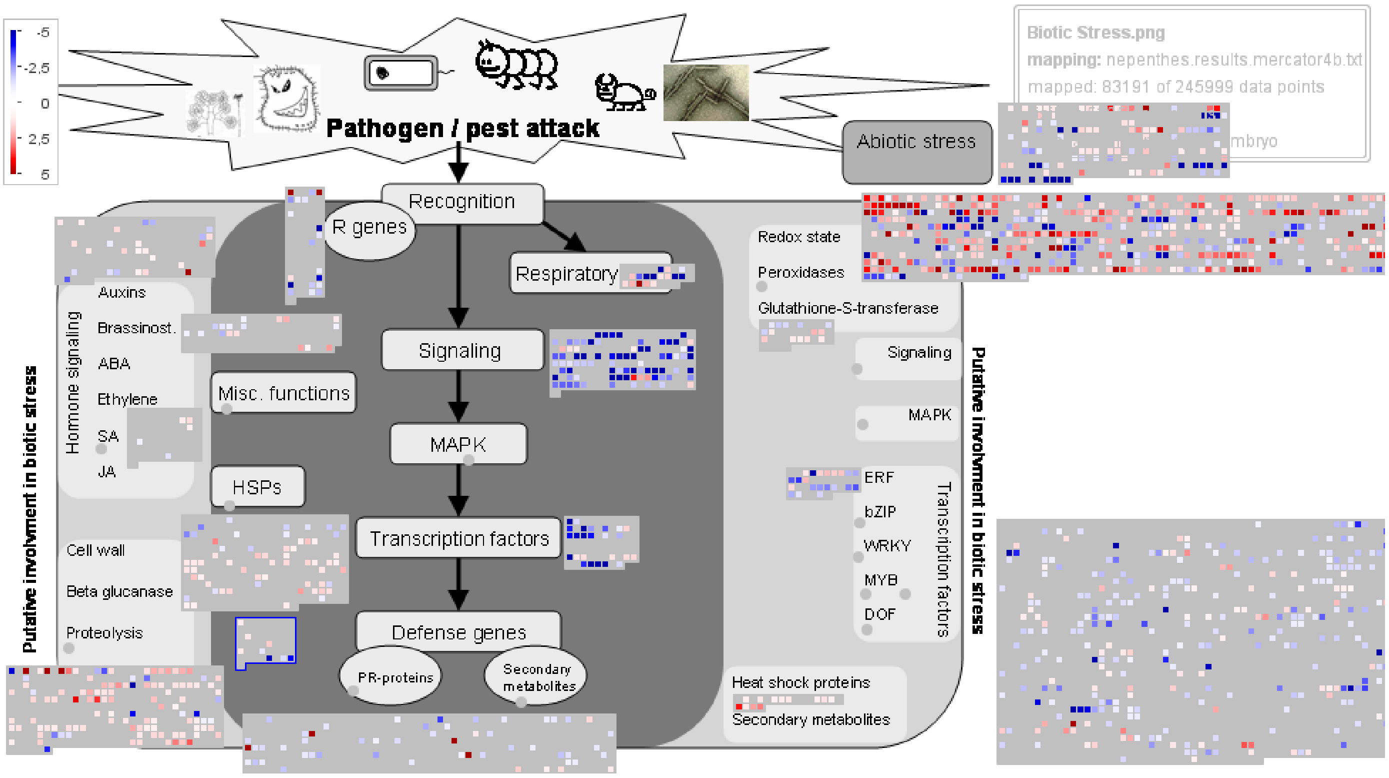
Pathogen / pest attack
Abiotic stress
Biotic Stress.png
mapping: nepenthes.results.mercator4b.txt
mapped: 83191 of 245999 data points
Recognition
R genes
Respiratory
Signaling
MAPK
Transcription factors
Defense genes
PR-proteins
Secondary metabolites
Misc. functions
HSPs
Hormone signaling
Auxins
Brassinost.
ABA
Ethylene
SA
JA
Cell wall
Beta glucanase
Proteolysis
Putative involvment in biotic stress
Redox state
Peroxidases
Glutathione-S-transferase
Signaling
MAPK
ERF
bZIP
WRKY
MYB
DOF
Transcription factors
Heat shock proteins
Secondary metabolites
-5
-2,5
0
2,5
5

d

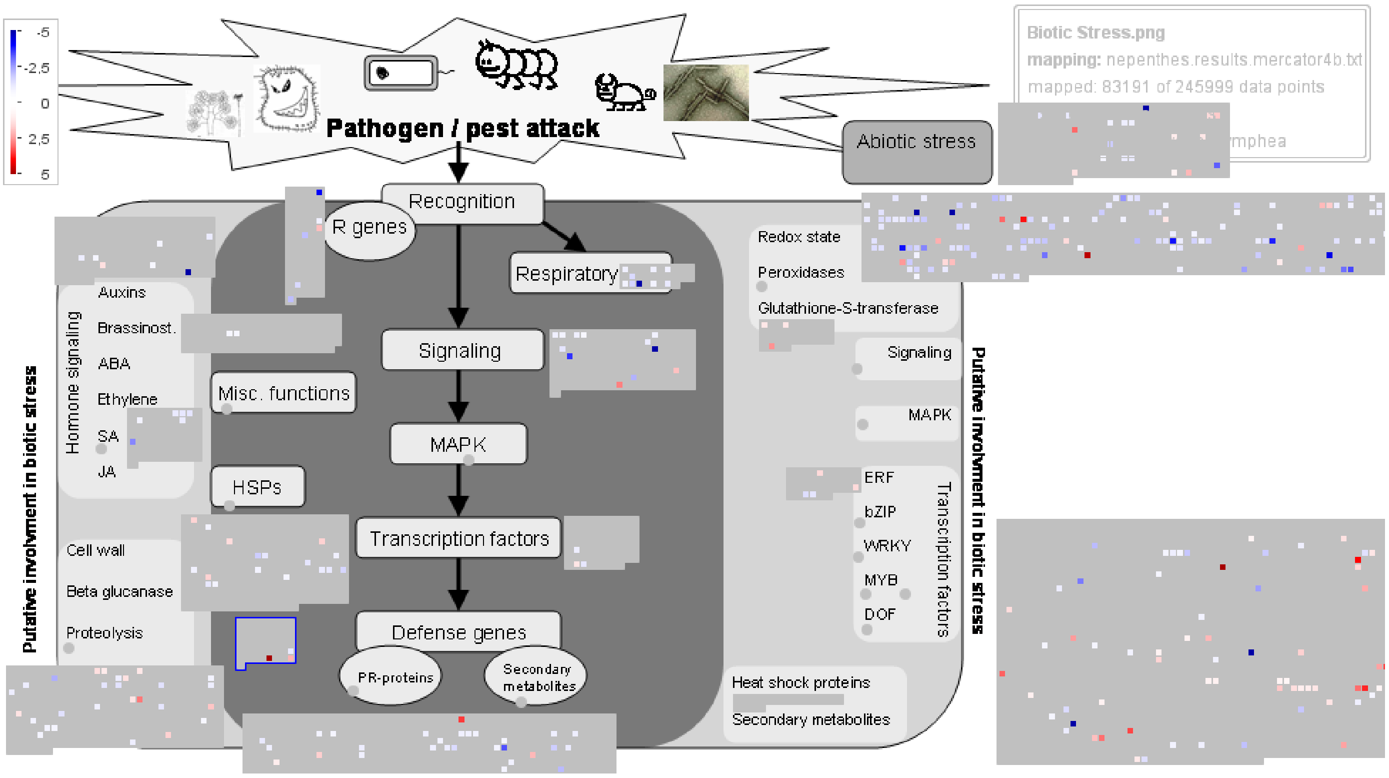
Pathogen / pest attack
Abiotic stress
Biotic Stress.png
mapping: nepenthes.results.mercator4b.txt
mapped: 83191 of 245999 data points
Recognition
R genes
Respiratory
Signaling
MAPK
Transcription factors
Defense genes
PR-proteins
Secondary metabolites
Misc. functions
HSPs
Hormone signaling
Auxins
Brassinost.
ABA
Ethylene
SA
JA
Cell wall
Beta glucanase
Proteolysis
Putative involvment in biotic stress
Redox state
Peroxidases
Glutathione-S-transferase
Signaling
MAPK
ERF
bZIP
WRKY
MYB
DOF
Transcription factors
Heat shock proteins
Secondary metabolites
-5
-2,5
0
2,5
5

Figure S6

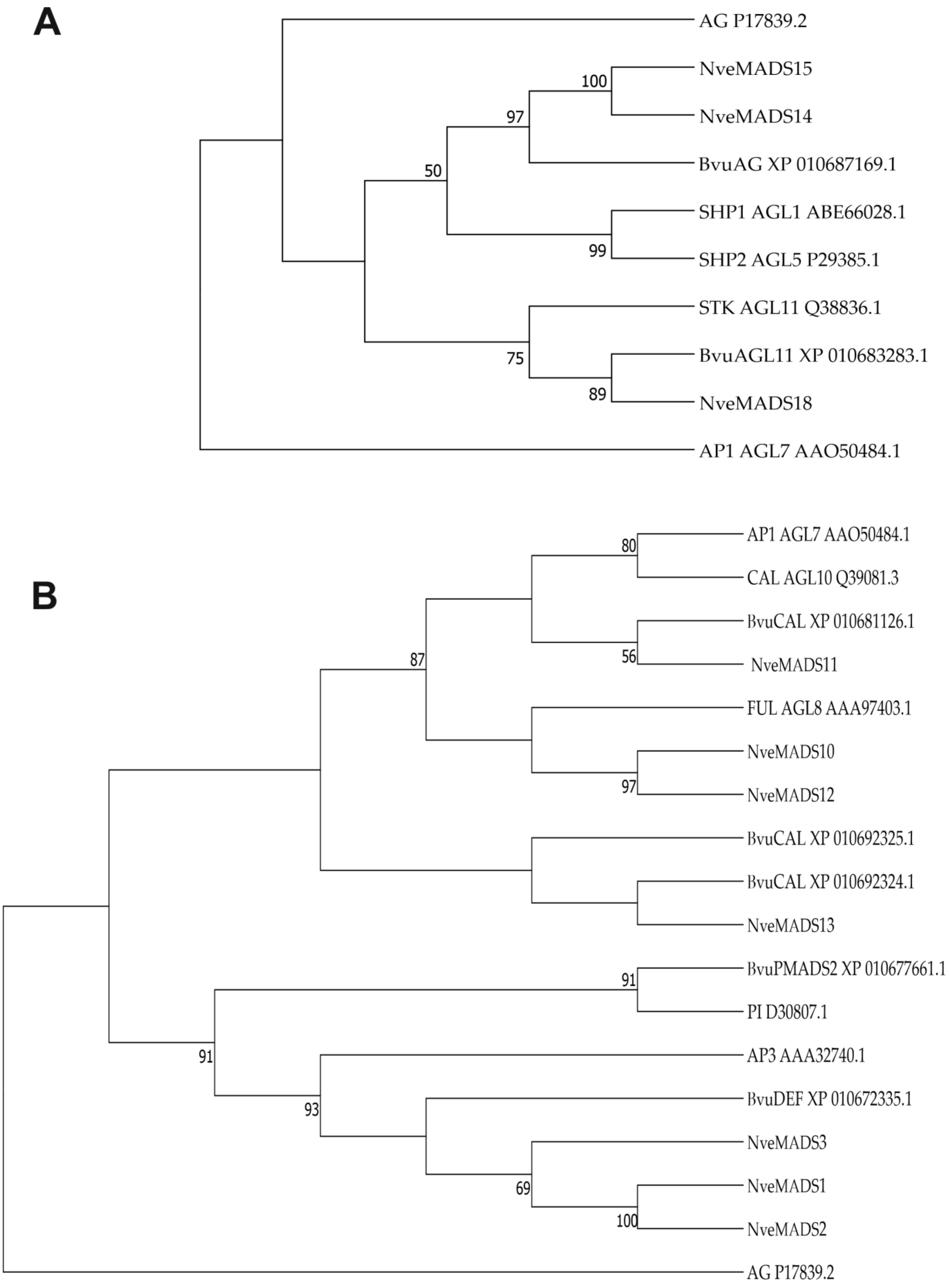
A
AG P17839.2
NveMADS15
NveMADS14
BvuAG XP 010687169.1
SHP1 AGL1 ABE66028.1
SHP2 AGL5 P29385.1
STK AGL11 Q38836.1
BvuAGL11 XP 010683283.1
NveMADS18
AP1 AGL7 AAO50484.1
100
97
50
99
75
89
B
AP1 AGL7 AAO50484.1
CAL AGL10 Q39081.3
BvuCAL XP 010681126.1
NveMADS11
FUL AGL8 AAA97403.1
NveMADS10
NveMADS12
BvuCAL XP 010692325.1
BvuCAL XP 010692324.1
NveMADS13
BvuPMADS2 XP 010677661.1
PI D30807.1
AP3 AAA32740.1
BvuDEF XP 010672335.1
NveMADS3
NveMADS1
NveMADS2
AG P17839.2
80
56
87
97
91
91
93
69
100

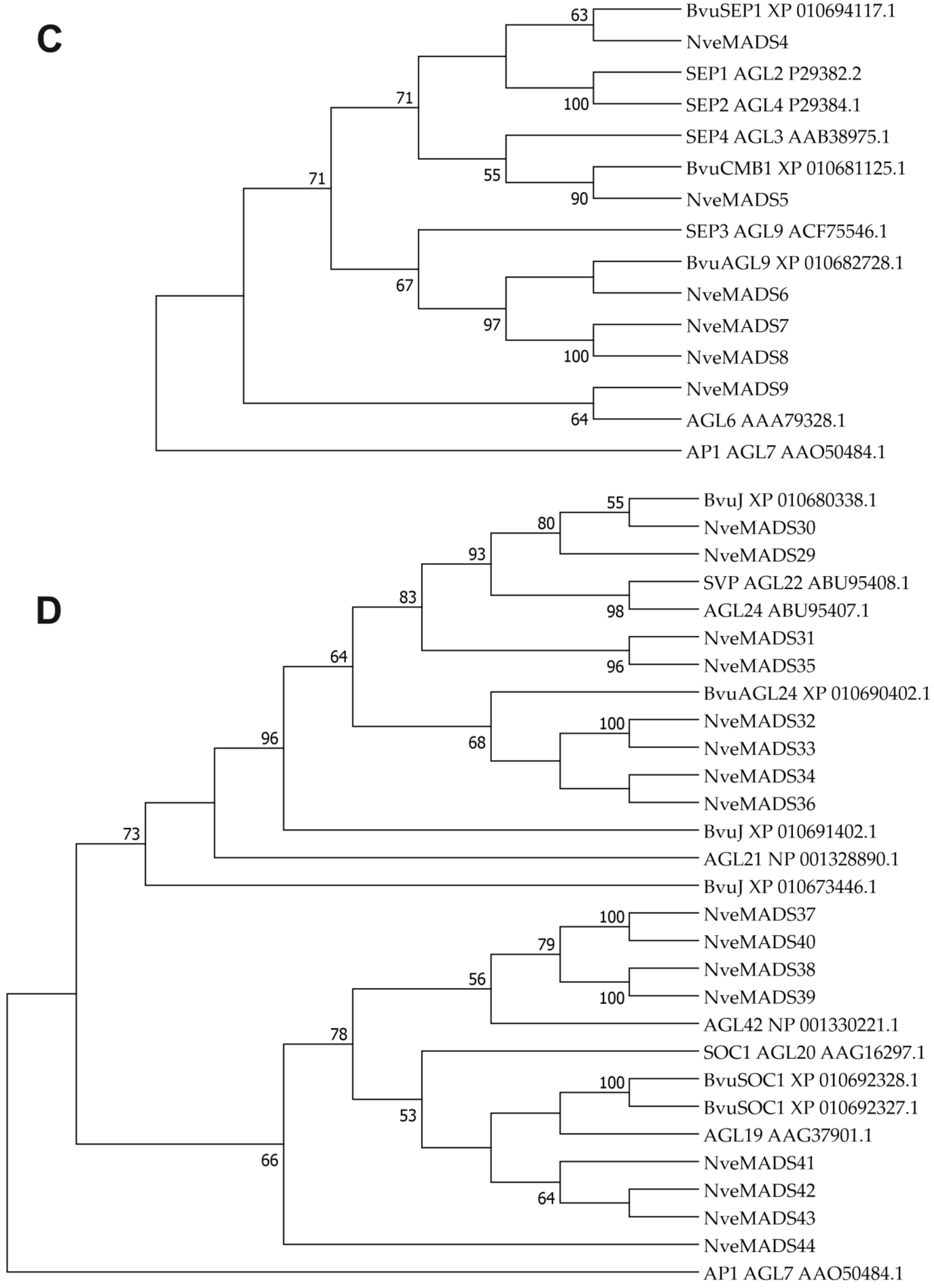
C
BvuSEP1 XP 010694117.1
NveMADS4
SEP1 AGL2 P29382.2
SEP2 AGL4 P29384.1
SEP4 AGL3 AAB38975.1
BvuCMB1 XP 010681125.1
NveMADS5
SEP3 AGL9 ACF75546.1
BvuAGL9 XP 010682728.1
NveMADS6
NveMADS7
NveMADS8
NveMADS9
AGL6 AAA79328.1
AP1 AGL7 AAO50484.1
D
BvuJ XP 010680338.1
NveMADS30
NveMADS29
SVP AGL22 ABU95408.1
AGL24 ABU95407.1
NveMADS31
NveMADS35
BvuAGL24 XP 010690402.1
NveMADS32
NveMADS33
NveMADS34
NveMADS36
BvuJ XP 010691402.1
AGL21 NP 001328890.1
BvuJ XP 010673446.1
NveMADS37
NveMADS40
NveMADS38
NveMADS39
AGL42 NP 001330221.1
SOC1 AGL20 AAG16297.1
BvuSOC1 XP 010692328.1
BvuSOC1 XP 010692327.1
AGL19 AAG37901.1
NveMADS41
NveMADS42
NveMADS43
NveMADS44
AP1 AGL7 AAO50484.1

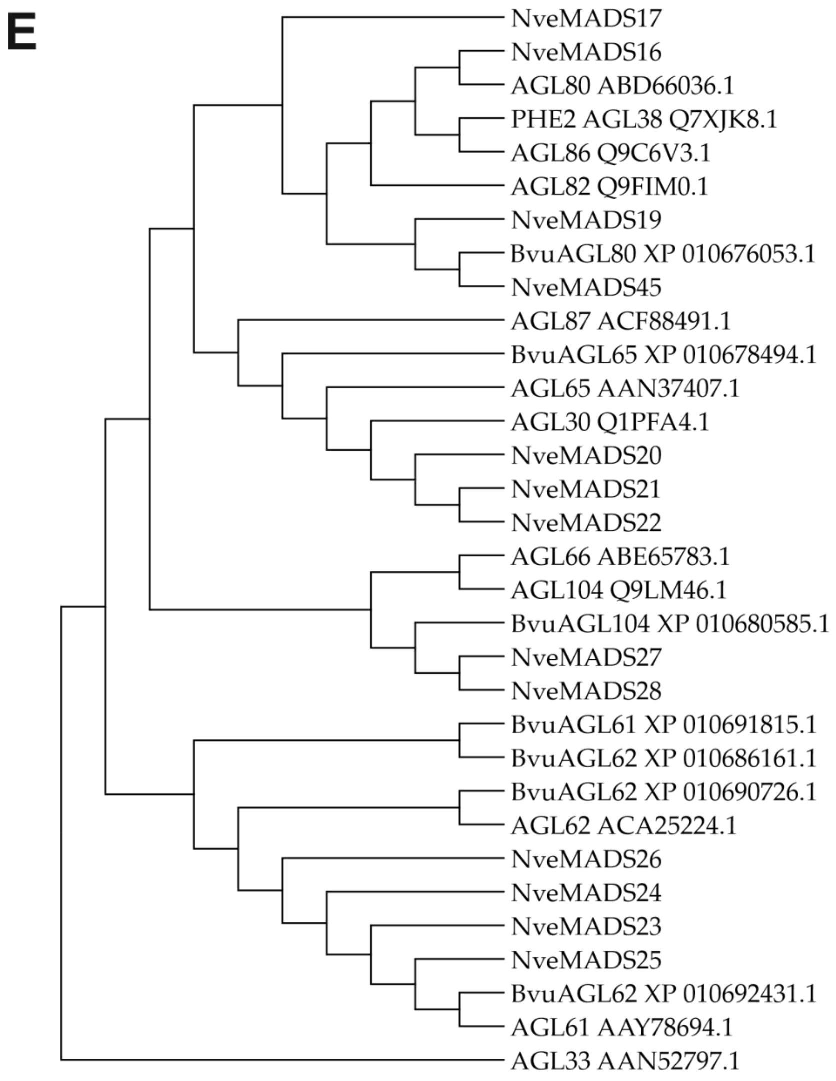
E
NveMADS17
NveMADS16
AGL80 ABD66036.1
PHE2 AGL38 Q7XJK8.1
AGL86 Q9C6V3.1
AGL82 Q9FIM0.1
NveMADS19
BvuAGL80 XP 010676053.1
NveMADS45
AGL87 ACF88491.1
BvuAGL65 XP 010678494.1
AGL65 AAN37407.1
AGL30 Q1PFA4.1
NveMADS20
NveMADS21
NveMADS22
AGL66 ABE65783.1
AGL104 Q9LM46.1
BvuAGL104 XP 010680585.1
NveMADS27
NveMADS28
BvuAGL61 XP 010691815.1
BvuAGL62 XP 010686161.1
BvuAGL62 XP 010690726.1
AGL62 ACA25224.1
NveMADS26
NveMADS24
NveMADS23
NveMADS25
BvuAGL62 XP 010692431.1
AGL61 AAY78694.1
AGL33 AAN52797.1

Figure S7

NveMADS1

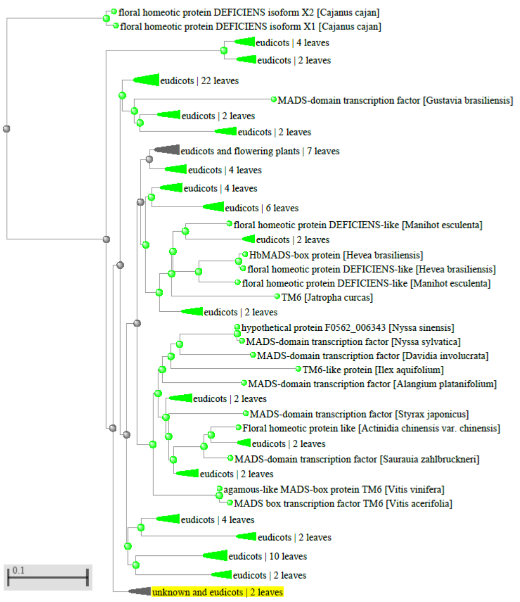

NveMADS2

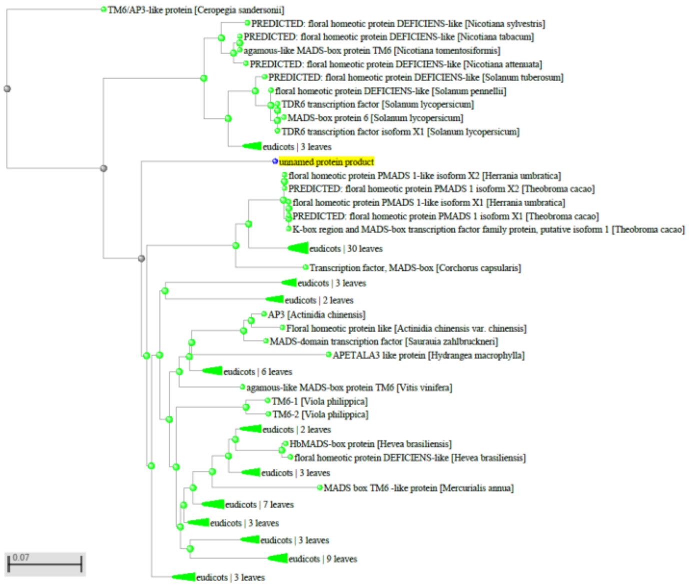

NveMADS3

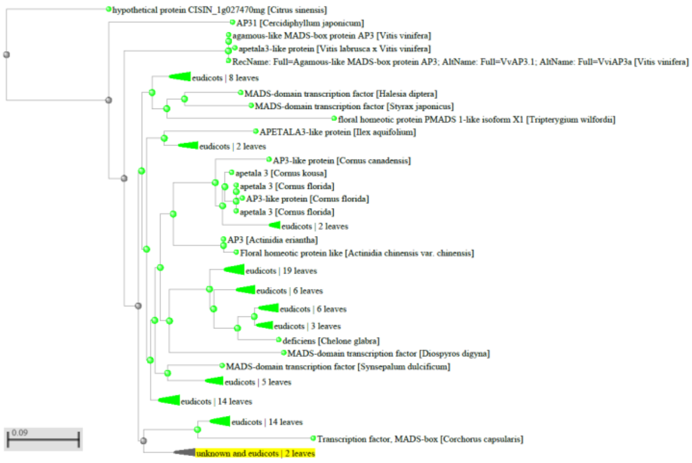

NveMADS4

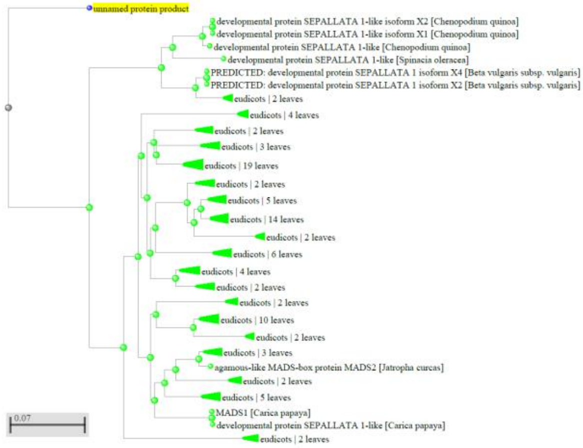

NveMADS5

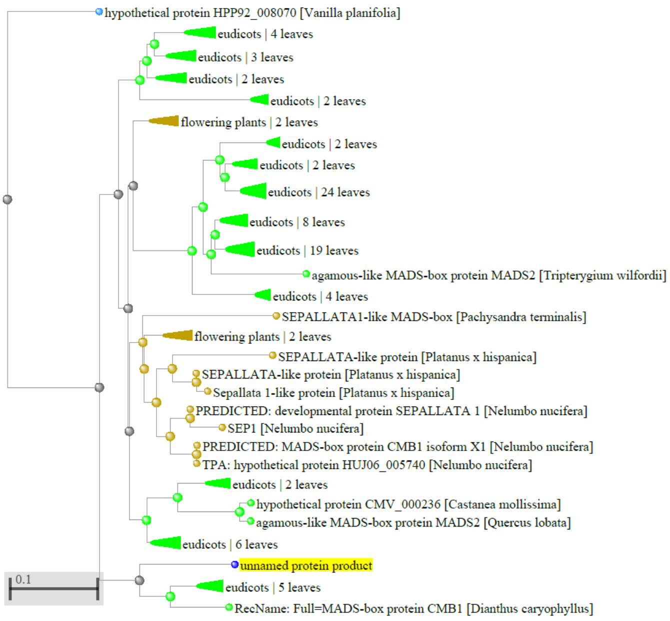

NveMADS6

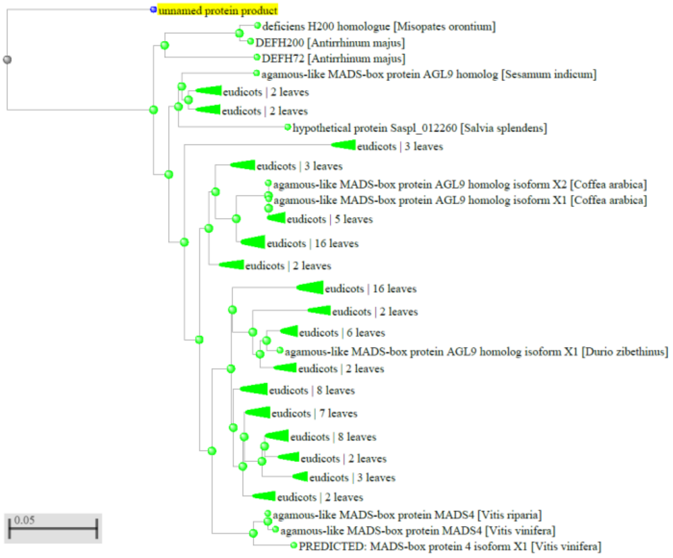

NveMADS7

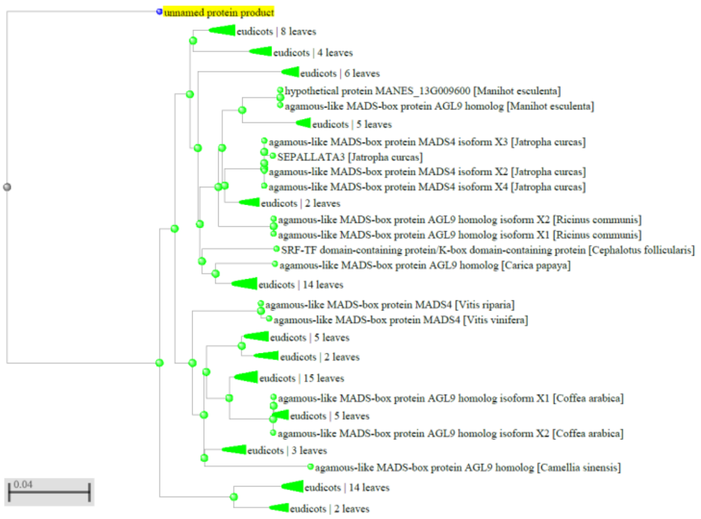

NveMADS8

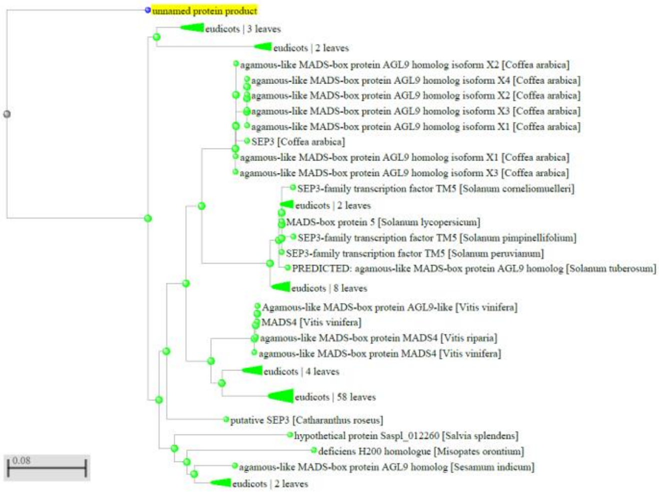

NveMADS9

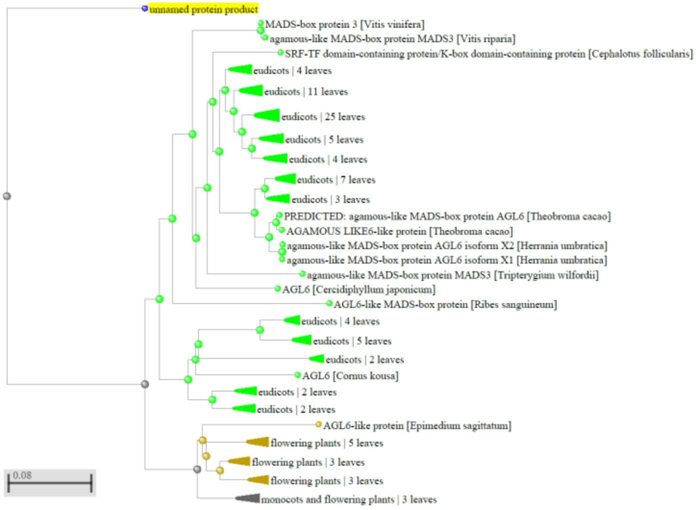

NveMADS10

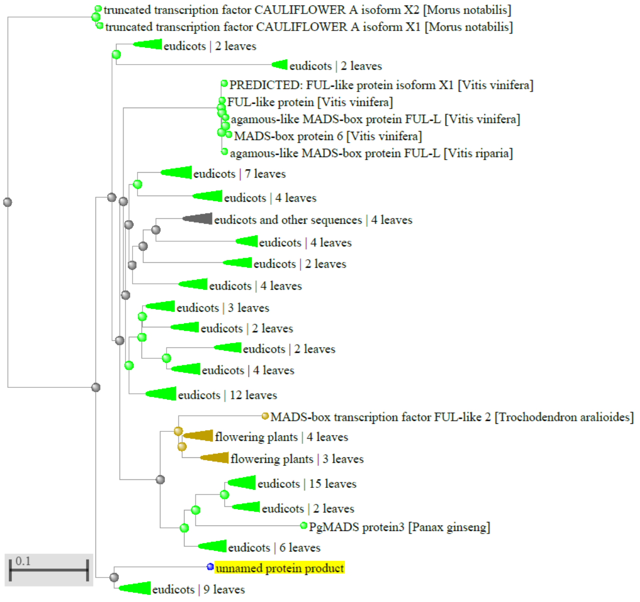

NveMADS11

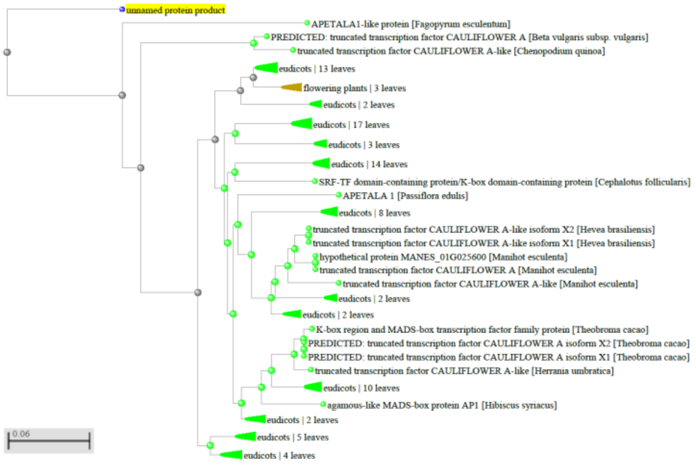

NveMADS12

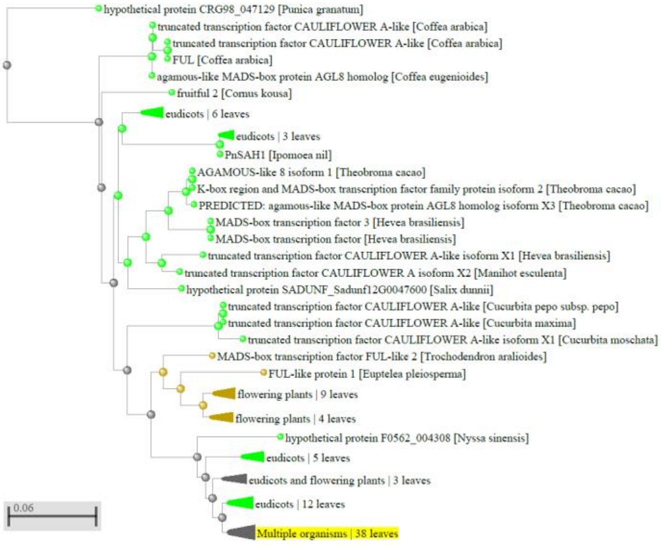

NveMADS13

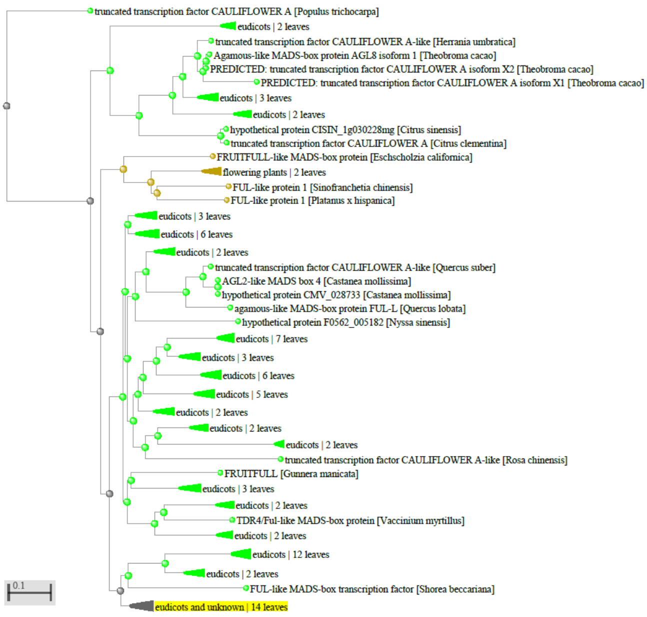

NveMADS14

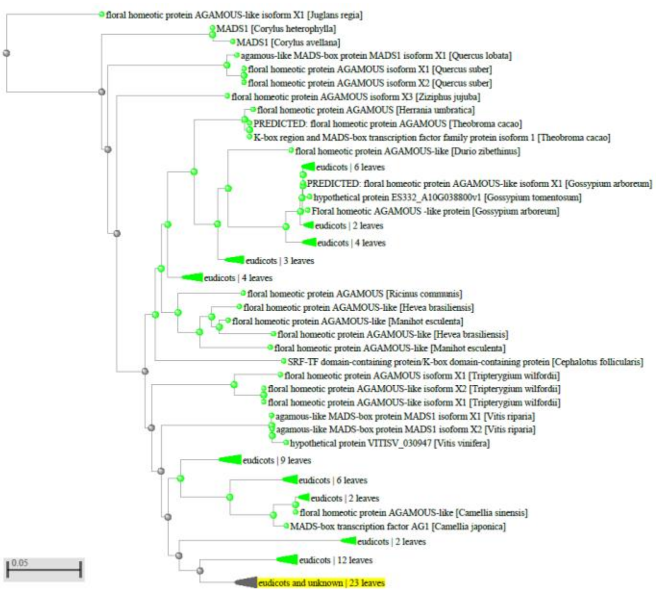

NveMADS15

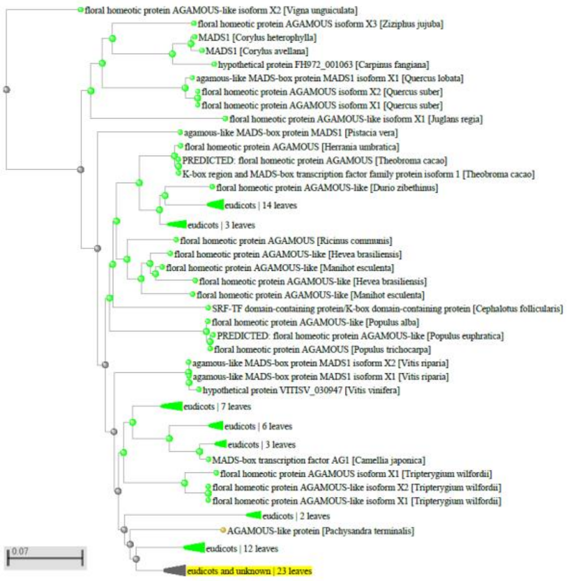

NveMADS16

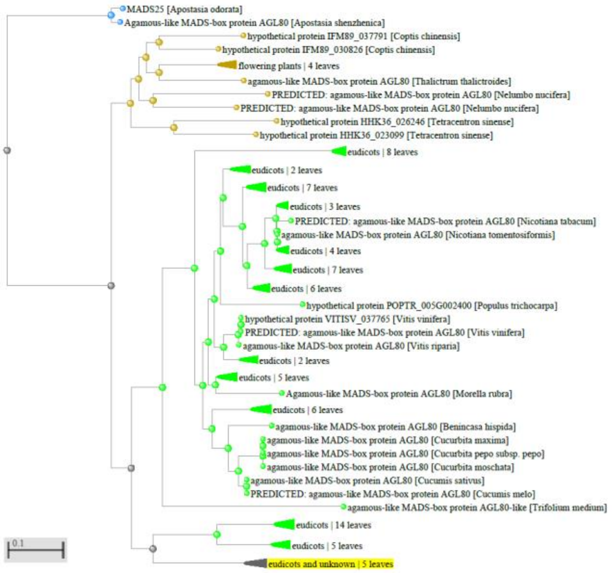

NveMADS17

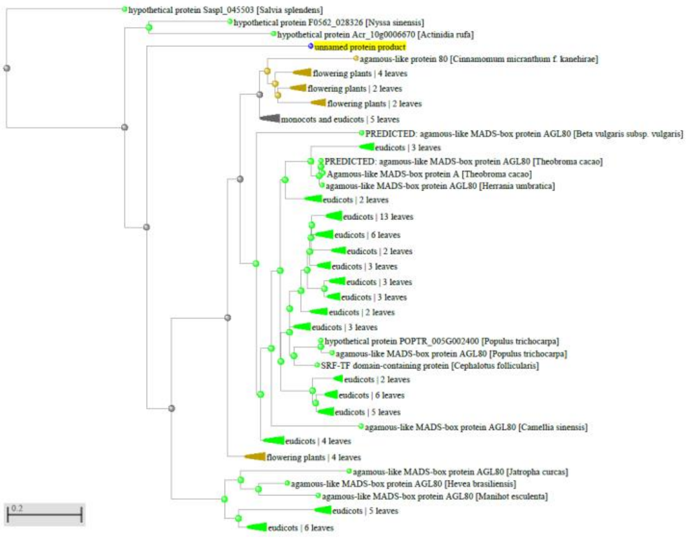

NveMADS18

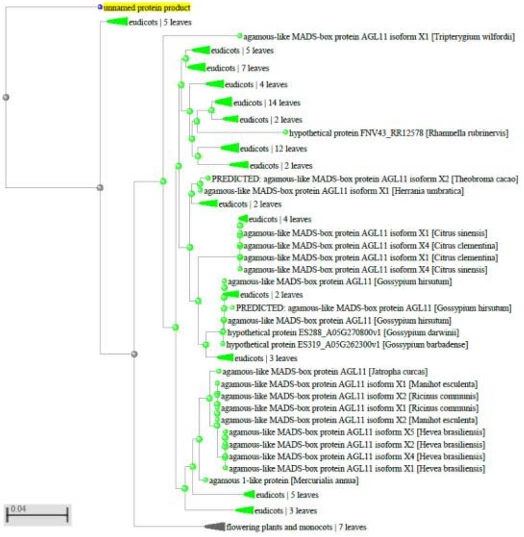

NveMADS19

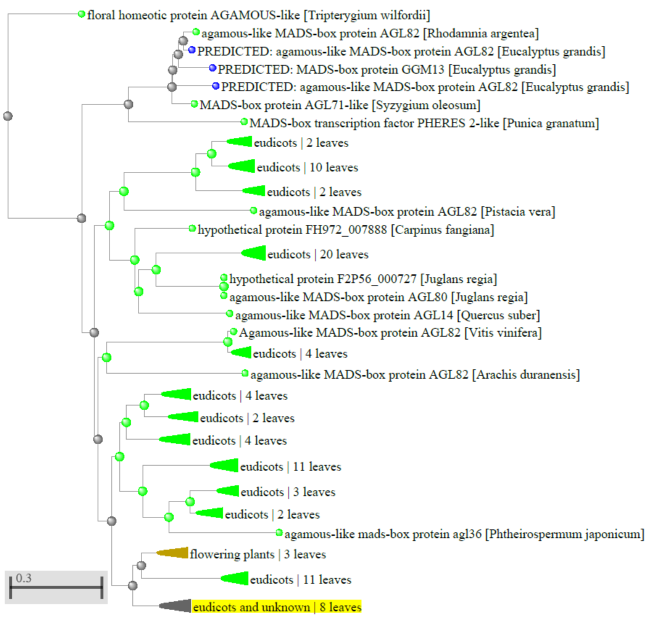

NveMADS20

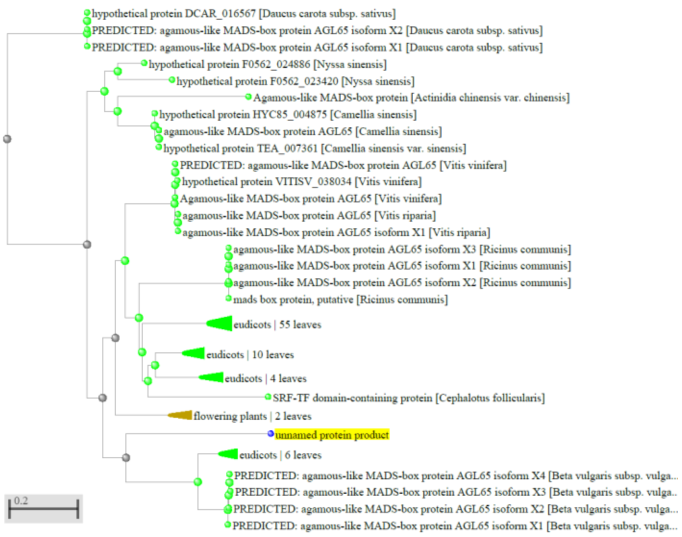

NveMADS21

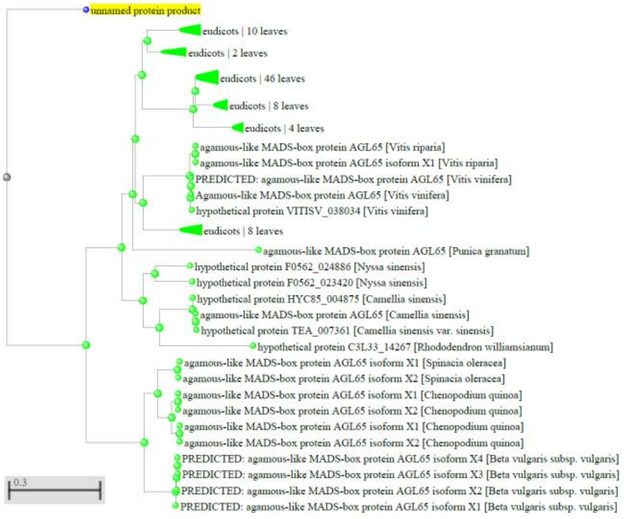

NveMADS22

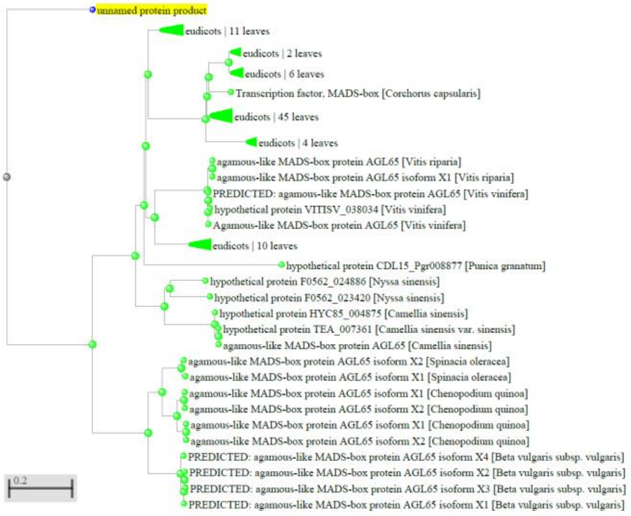

NveMADS23

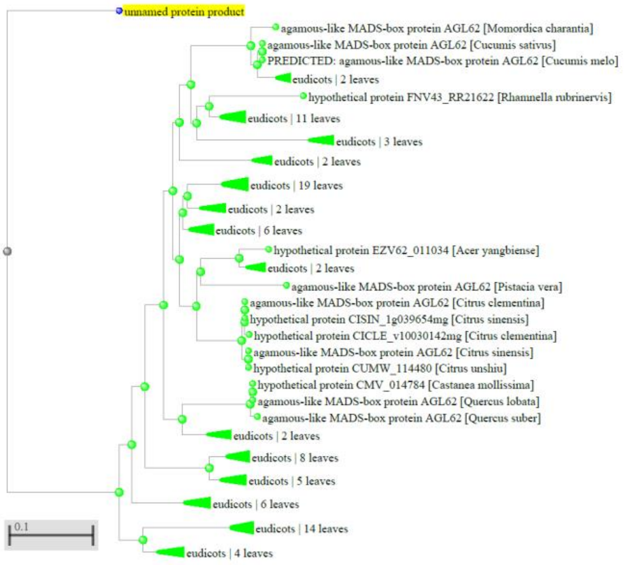

NveMADS24

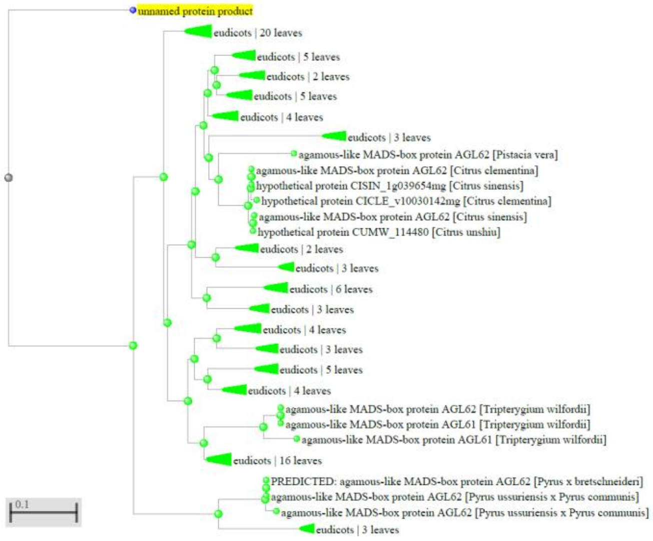

NveMADS25

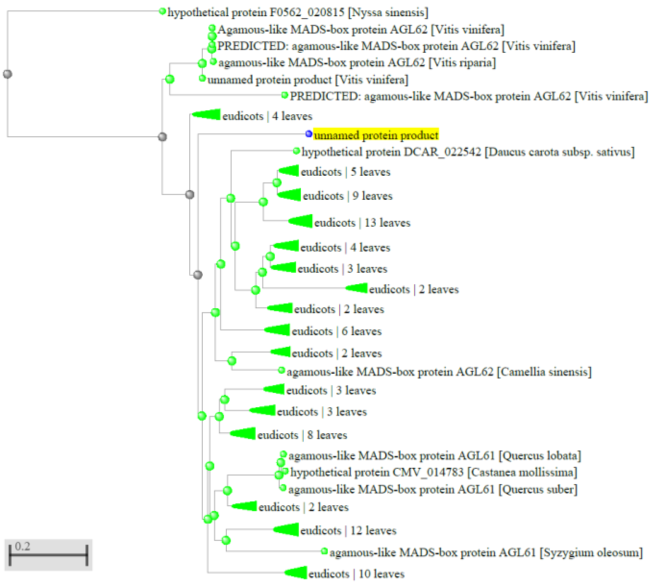

NveMADS26

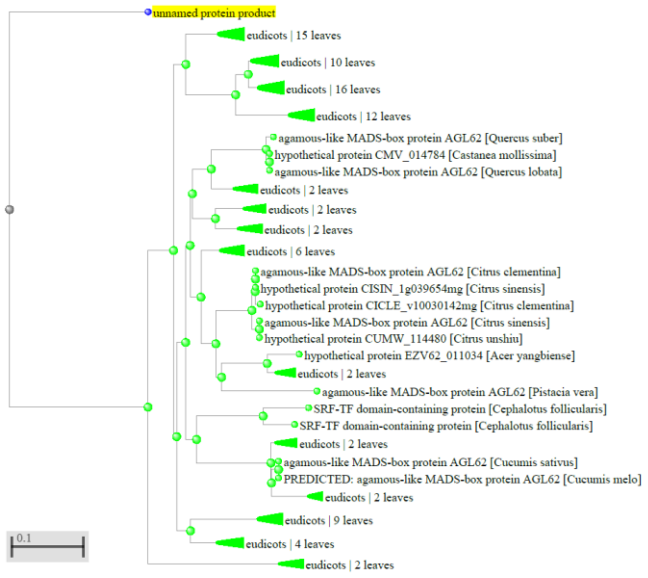

NveMADS27

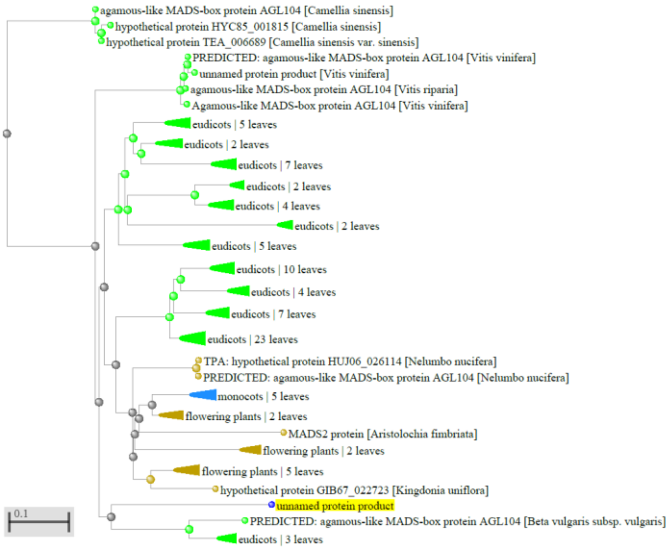

NveMADS28

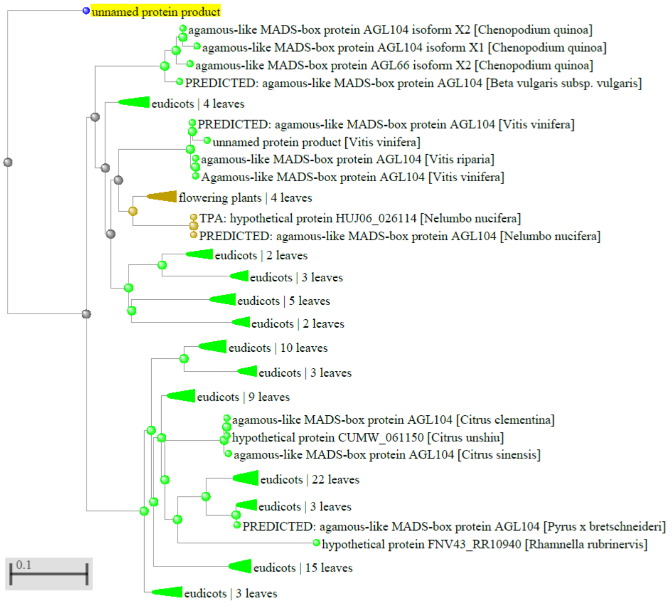

NveMADS29

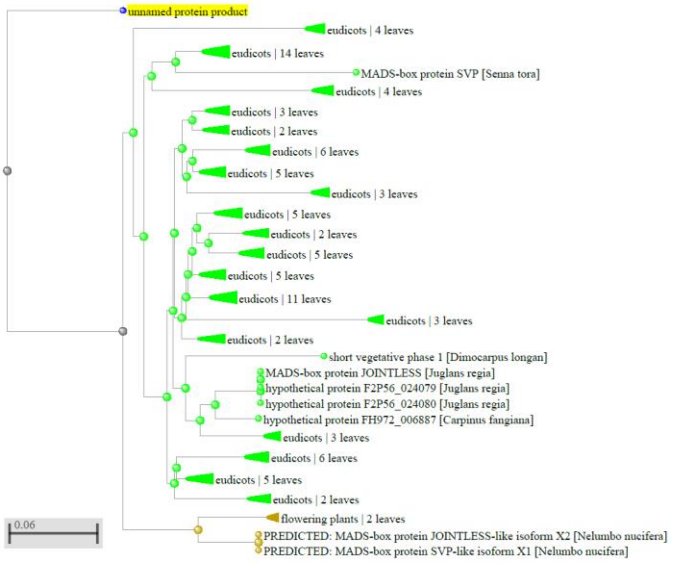

NveMADS30

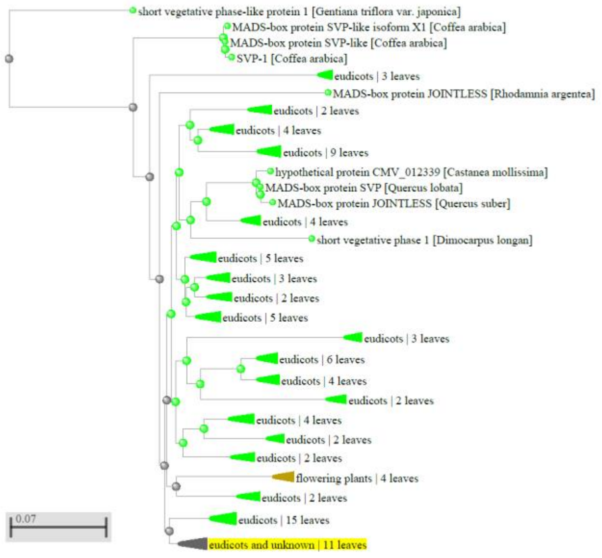

NveMADS31

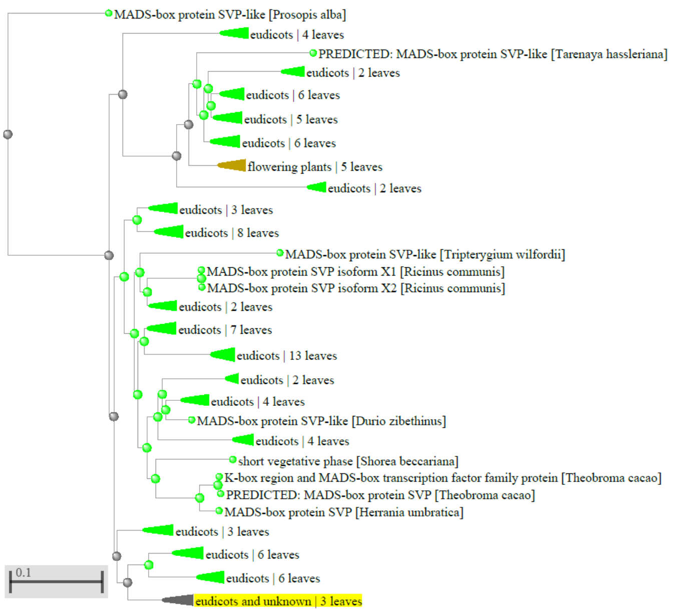

NveMADS32

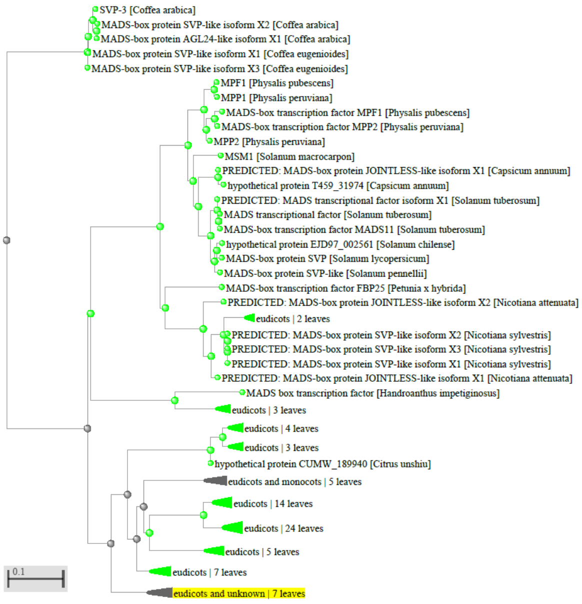

NveMADS33

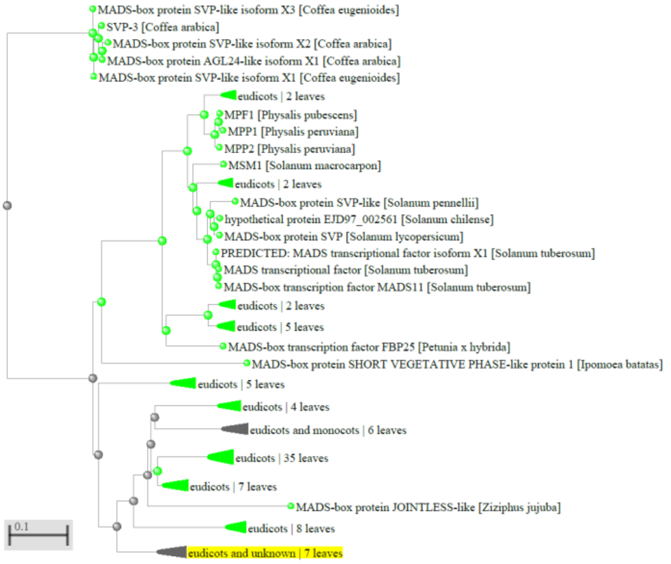

NveMADS34

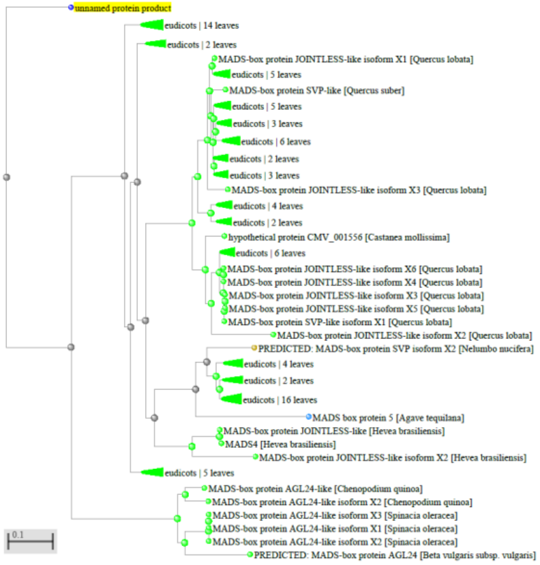

NveMADS35

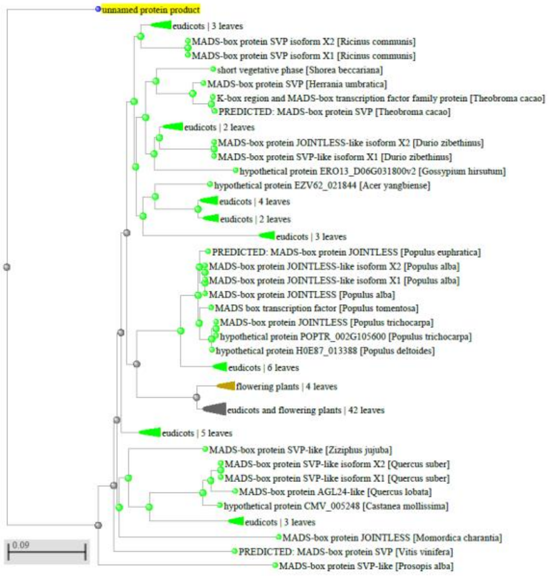

NveMADS36

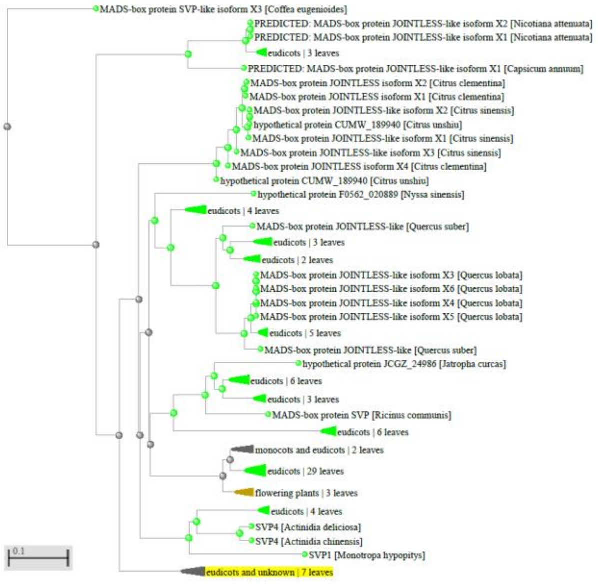

NveMADS37

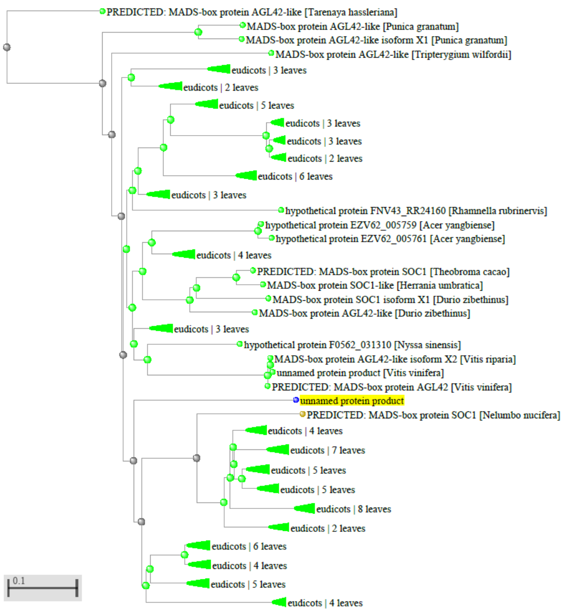

NveMADS38

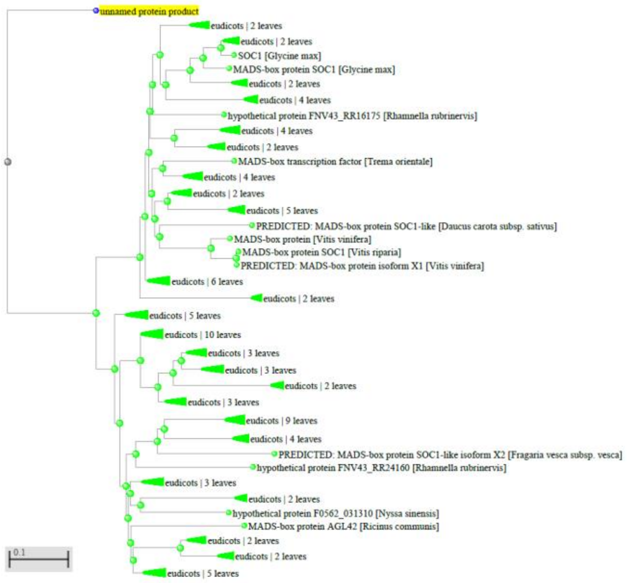

NveMADS39

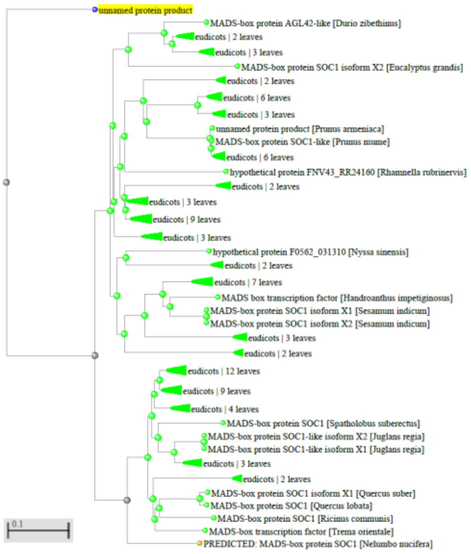

NveMADS40

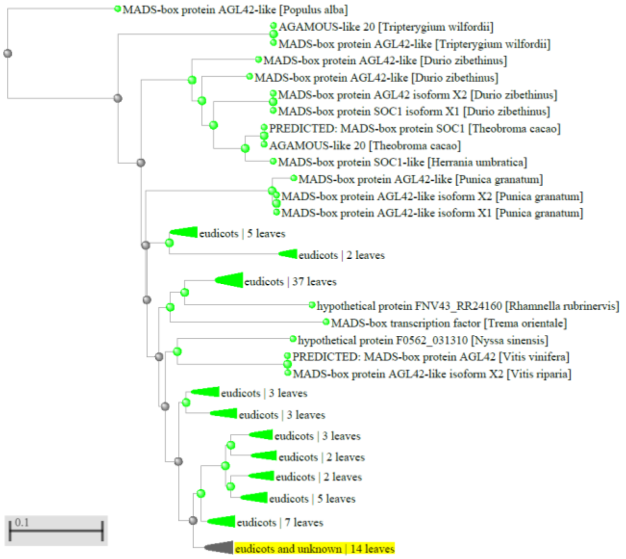

NveMADS41

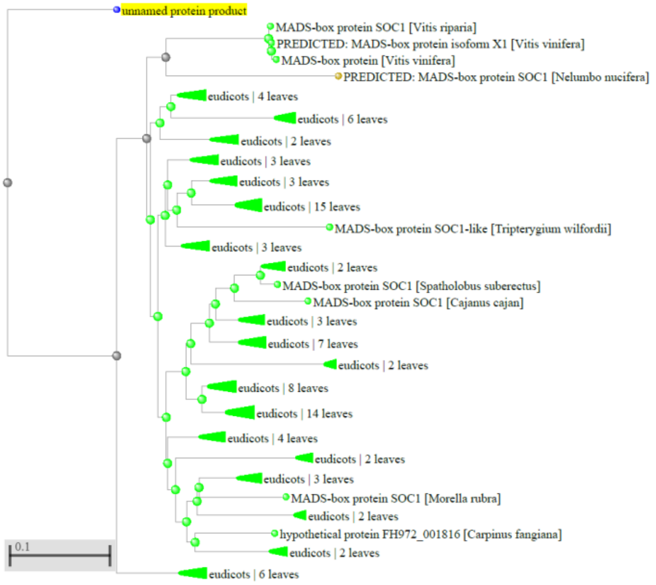

NveMADS42

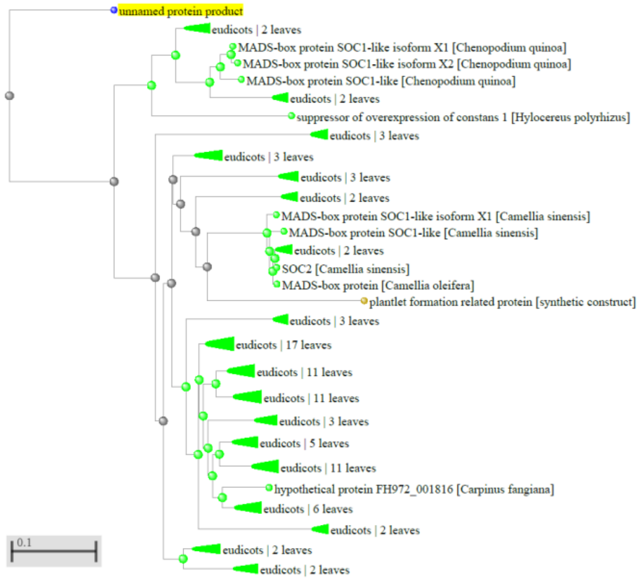

NveMADS43

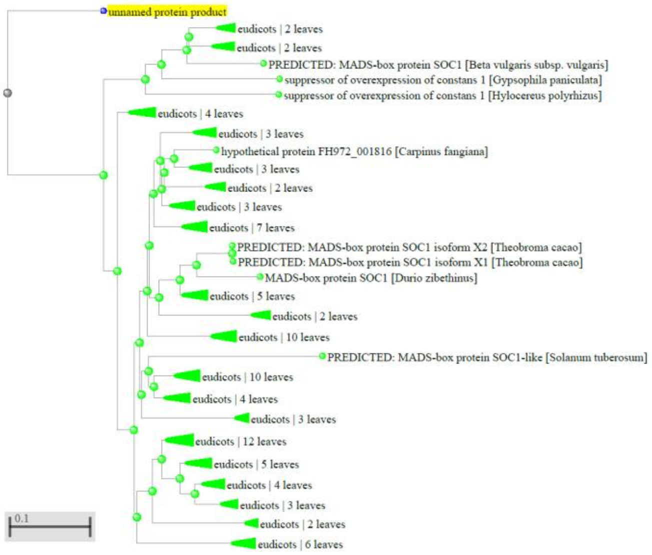

NveMADS44

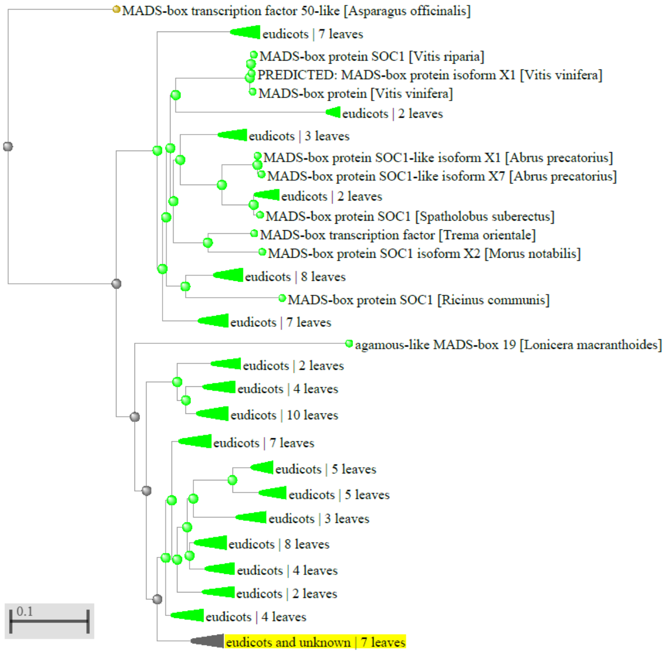

NveMADS45

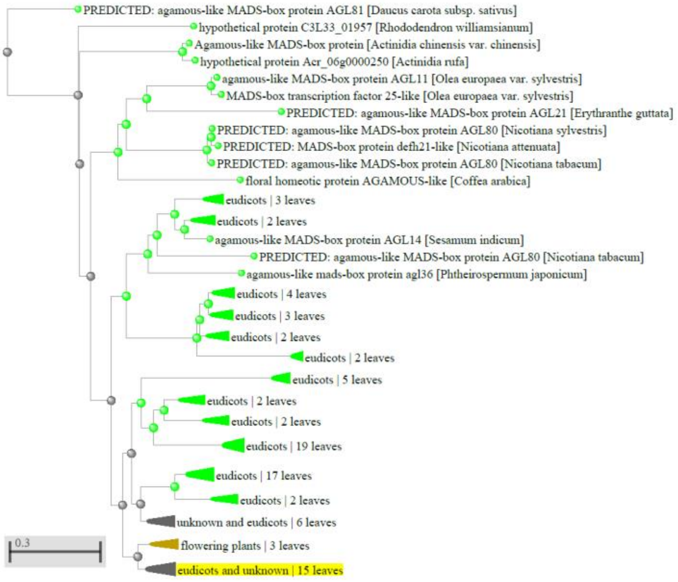

Figure S8

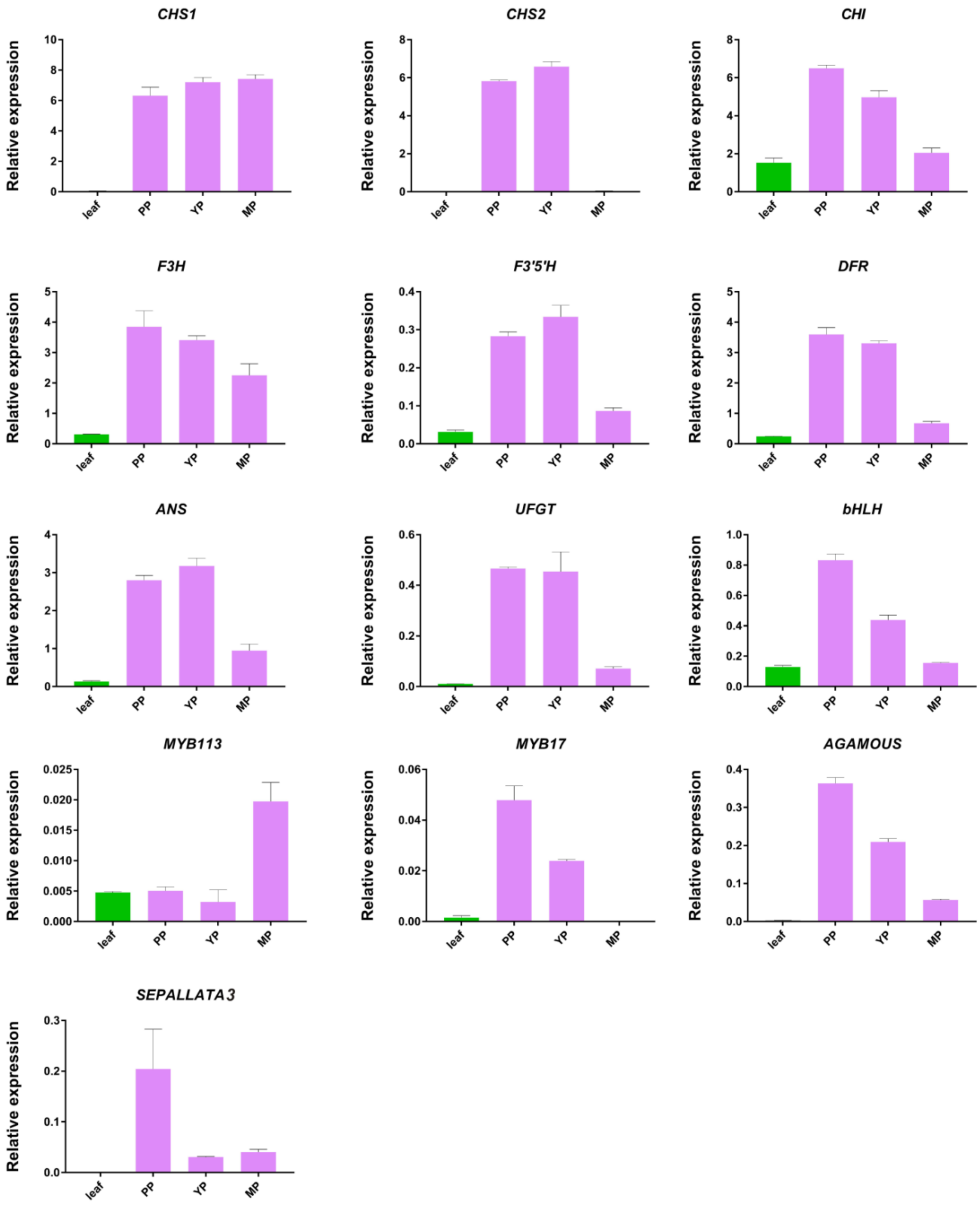
CHS1
CHS2
CHI
F3H
F3'5'H
DFR
ANS
UFGT
bHLH
MYB113
MYB17
AGAMOUS
SEPALLATA3
Relative expression
leaf
PP
YP
MP
